# The Long-Term Benefits of Increased Aspirin Use by At-Risk Americans Aged 50 and Older

**DOI:** 10.1371/journal.pone.0166103

**Published:** 2016-11-30

**Authors:** David B. Agus, Étienne Gaudette, Dana P. Goldman, Andrew Messali

**Affiliations:** 1 Lawrence J. Ellison Institute for Transformative Medicine, University of Southern California Keck School of Medicine and Viterbi School of Engineering, Beverly Hills, California, United States of America; 2 Schaeffer Center for Health Policy and Economics, University of Southern California Price School and School of Pharmacy, Los Angeles, California, United States of America; 3 Analysis Group, Inc. Boston, Massachusetts, United States of America; Morehouse School of Medicine, UNITED STATES

## Abstract

**Background:**

The usefulness of aspirin to defend against cardiovascular disease in both primary and secondary settings is well recognized by the medical profession. Multiple studies also have found that daily aspirin significantly reduces cancer incidence and mortality. Despite these proven health benefits, aspirin use remains low among populations targeted by cardiovascular prevention guidelines. This article seeks to determine the long-term economic and population-health impact of broader use of aspirin by older Americans at higher risk for cardiovascular disease.

**Methods and Findings:**

We employ the Future Elderly Model, a dynamic microsimulation that follows Americans aged 50 and older, to project their lifetime health and spending under the status quo and in various scenarios of expanded aspirin use. The model is based primarily on data from the Health and Retirement Study, a large, representative, national survey that has been ongoing for more than two decades. Outcomes are chosen to provide a broad perspective of the individual and societal impacts of the interventions and include: heart disease, stroke, cancer, life expectancy, quality-adjusted life expectancy, disability-free life expectancy, and medical costs. Eligibility for increased aspirin use in simulations is based on the 2011–2012 questionnaire on preventive aspirin use of the National Health and Nutrition Examination Survey. These data reveal a large unmet need for daily aspirin, with over 40% of men and 10% of women aged 50 to 79 presenting high cardiovascular risk but not taking aspirin. We estimate that increased use by high-risk older Americans would improve national life expectancy at age 50 by 0.28 years (95% CI 0.08–0.50) and would add 900,000 people (95% CI 300,000–1,400,000) to the American population by 2036. After valuing the quality-adjusted life-years appropriately, Americans could expect $692 billion (95% CI 345–975) in net health benefits over that period.

**Conclusions:**

Expanded use of aspirin by older Americans with elevated risk of cardiovascular disease could generate substantial population health benefits over the next twenty years and do so very cost-effectively.

## 1. Introduction

Cardiovascular disease, including heart attack and stroke, is the leading cause of death in the United States and a significant driver of health-care spending [1, 2]. The usefulness of aspirin to prevent cardiovascular disease is well recognized. Since the early 2000s, the US Preventive Services Task Force and the American Heart Association (AHA) have recommended aspirin for primary and secondary prevention of cardiovascular diseases [3–8]. Despite these long-standing guidelines, aspirin use remains low. A survey on preventive low-dose aspirin use was conducted by the National Health and Nutrition Examination Survey in 2011–2012 [9]. Using these data, we estimate that less than half the men and women eligible for daily aspirin actually use the medication.

The role of aspirin in primary cancer prevention is less settled but becoming clearer. Growing evidence suggests that daily long-term aspirin use plays a significant preventive role for colorectal [5, 10] and other cancers [11].

Daily aspirin appears to have tremendous potential to hit the triple aim of better care, better health, and smarter spending. To quantify that potential, this article seeks to determine the long-term economic and population-health impact of full guideline adherence, though simulations using the Future Elderly Model (FEM), an established, dynamic microsimulation that follows Americans aged 50 and older.

## 2. Methods

### 2.1 The Future Elderly Model

We conduct simulations using the FEM, a dynamic microsimulation developed by Goldman et al. to forecast the implications of different medical-technology scenarios on long-term health and health-care spending [12]. FEM follows Americans aged 50 and older and projects their health and medical spending over time. Its unique feature is to follow the evolution of individual-level health trajectories rather than average or aggregate characteristics of a cohort. In the recent past, researchers have used FEM for a variety of purposes, including forecasting the changing health status of the elderly Medicare population in the decades 2010–2030 [13]; estimating the impact of the introduction of statin medication on the costs of obesity [14]; and estimating the value of medical interventions to reduce obesity prevalence [15], delay Alzheimer’s disease [16], and delay the biology of aging [17, 18].

FEM simulates the lives of older Americans using the biennial Health and Retirement Study of Americans aged 51 and over; the large-scale Medical Expenditure Panel Survey of the noninstitutionalized US population; and the nationally representative Medicare Current Beneficiary Survey. These sources are used to compute the health-transition models at the core of FEM, project health-care spending, and assess quality of life during the simulations. We describe the model and methods briefly here; complete technical information is available in S1 File online.

FEM has three core modules. The first, Health Transitions, calculates transition probabilities across various health states based on the individual’s current characteristics. Health transitions include chronic disease incidence, functional status, body mass index, and mortality. These transition probabilities are modeled using first-order Markov processes that depend on a battery of predictors, such as age, sex, education, race, and health conditions.

Health conditions are derived from Health and Retirement Study survey questions. We treat chronic conditions as absorbing—once individuals receive a diagnosis, they are henceforth considered to have that condition. This interpretation is consistent with the Health and Retirement Study questionnaire, which asks respondents whether they were ever diagnosed with a condition. The body mass index variable is based on the self-reported height and weight of Health and Retirement Study respondents, projected with estimates of a log-linear model. Functional status is measured by daily living limitations and residence in a nursing home. We consider individuals disabled if they reported at least one limitation or lived in a nursing home, and we allow for transitions in and out of functional states.

To evaluate quality of life, we predict quality-adjusted life-years using the EQ-5D, a quality-of-life index based on five health-related variables addressing mobility, daily activities, self-care, anxiety, depression, and pain. Using the Medical Expenditure Panel Survey data, we apply an ordinary least squares regression to fit derived EQ-5D quality-adjustment scores as a function of the chronic conditions and functional states included in FEM simulations.

Based on two complementary medical-spending data sources, the Policy Outcomes module predicts an individual’s health spending with regard to health status, demographics, nursing home status, and mortality. Our estimates are based on spending data from the 2007–2010 Medical Expenditure Panel Survey for individuals younger than 65 and the 2007–2010 Medicare Current Beneficiary Survey for individuals aged 65 and older. The estimates are based on pooled least squares regressions of spending on risk factors, self-reported conditions, and functional status, with spending inflated to current dollars.

Finally, the Replenishing Cohorts module predicts economic and health outcomes of new cohorts of 51-year-olds, using data from the Health and Retirement Study, the National Health and Nutrition Examination Survey (NHANES), the National Health Interview Survey, and the American Community Survey.

### 2.2 Simulations

We conduct two types of simulations. In *cohort simulations*, we follow a cohort of individuals aged 50–51 in 2009, and track their outcomes from 2010 until death. In these simulations, individuals composing the cohort ‘age’ each period. In *population simulations*, we model outcomes for all Americans aged 51 and older until 2050, taking into account those Americans who ‘age into’ the model (using our Replenishing Cohorts). In these simulations, we can observe a representative cross-section of the older US population in each period. Since the Health and Retirement Study is biennial, we simulate health and costs over two-year periods in both simulation types.

#### 2.2.1 Scenarios

We consider two main scenarios:

- The *Guideline Adherence* scenario provides the health benefits and side effects of aspirin to all individuals for whom aspirin is recommended by the US Preventive Services Task Force and AHA guidelines for primary and secondary prevention. We consider the aspirin-use guidelines that were effective for the 2011–2012 NHANES. With regard to primary prevention therapy, these guidelines specify a series of ten-year thresholds for coronary heart disease and stroke risk over which men and women are eligible [4]. With regard to secondary prevention therapy, this scenario considers individuals with prior stroke or cardiovascular disease as eligible, as specified by AHA and ACCF guidelines [8]. Comparisons between baseline FEM projections (Status Quo scenario) and this scenario illustrate the guidelines’ full potential and the benefits lost because of low aspirin use.
- The *Universal Eligibility* scenario provides the health effects of aspirin to all Americans aged 51 and older, while aiming to provide an *upper bound* of the impact of additional aspirin use for US elderly rather than a realistic assessment of the impact that would occur if everyone aged 51 and older used aspirin daily.

#### 2.2.2 Eligibility

**Fig 1:**
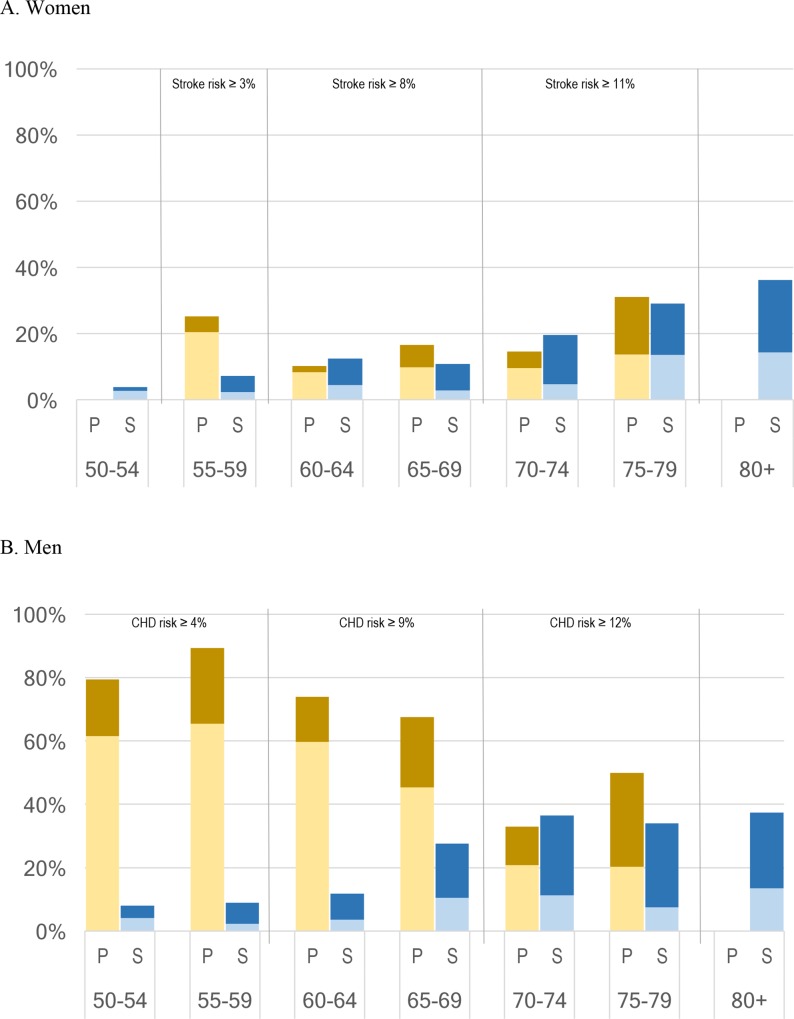
Eligibility for Daily Aspirin and Observed Use vs. Age, NHANES 2011–2012. Source: NHANES 2011–2012; author’s calculations. Light shading indicates that respondents are eligible for daily aspirin under 2009 USPSTF and 2011 AHA/ACCF guidelines and report using it; dark shading indicates that survey respondents are eligible for but report not using daily aspirin. P = eligibility for aspirin use in a primary prevention setting (2009 USPSTF guidelines; thresholds shown at the top of the figure); S = eligibility for aspirin in a secondary prevention setting (2011 AHA/AACCF guidelines). CHD refers to coronary heart disease. Eligibility for primary prevention daily use of aspirin is assigned to NHANES respondents based on USPSTF thresholds and established ten-year risk calculators for coronary heart disease and stroke. Eligibility for secondary prevention is assigned to respondents reporting that they had received a prior diagnosis of a stroke, heart disease, or both.

Since a subset of the population eligible for aspirin already takes it, we identify in FEM simulations individuals admissible for aspirin use under each scenario but *not* using aspirin. NHANES data contain detailed information about clinical biomarkers, disease risk factors, and aspirin use. In Fig 1, we compare the population eligible for aspirin in a primary or secondary prevention setting against reported aspirin use. Primary prevention eligibility, which corresponds to ten-year risks of coronary heart disease and stroke above the guideline thresholds, is shown in yellow; secondary prevention eligibility, which corresponds to prior stroke or heart disease, is shown in blue. The darker section shows the proportion of the eligible population that reported using daily aspirin.

These data reveal a large unmet need for daily aspirin. Most men aged 50–69 presented coronary heart disease risks above which aspirin use was recommended, but less than 20% were following the guidelines (pale yellow). Fewer women presented a risk of stroke above the thresholds recommended for aspirin use, but over 10% of women over 55 were eligible for aspirin but not taking it (see details in technical documentation provided online).

#### 2.2.3 Health impact and costs of aspirin

Table A in S2 File summarizes the most important findings from recent meta-analyses of health transitions and outcomes of eligible individuals to reflect the health impacts of aspirin in clinical trials. For eligible individuals, we decrease the probabilities of contracting heart disease, experiencing stroke, and mortality, and increase gastrointestinal bleeding risks. Table 1 shows how we incorporate differential effects by sex and context.

**Table 1:** Impact of Aspirin on the Probability of Selected Health Events.

	Point estimate	95% CI	Source
Heart disease			
Primary prevention context	0.90	0.85–0.96	Seshasai et al., 2012 [19]
Secondary prevention context	0.79	0.72–0.88	Berger et al., 2008 [20]
Stroke			
Men in a primary prevention context	1.11	0.96–1.33	Berger et al., 2006 [21]
Women in a primary prevention context	0.83	0.70–0.97	Berger et al., 2006 [21]
Both sexes in a secondary prevention context	0.75	0.65–0.87	Berger et al., 2008 [20]
Cancer	0.94*		Cuzick et al., 2014 [11]
All-cause mortality			
Primary prevention context	0.94	0.88–1.00	Seshasai et al., 2012 [19]
Secondary prevention context	0.87	0.76–1.00	Berger et al., 2008 [20]
Gastrointestinal bleeding	1.71	1.41–2.08	McQuaid and Laine, 2006 [22]

The table shows the distribution of factors by which health event probabilities are multiplied in FEM simulations for daily aspirin users. Table A in S2 File presents a more detailed overview of findings from recent meta-analyses. S3 File—Technical Documentation includes a discussion of each parameter and its implementation in the microsimulation.

*: While no comprehensive meta-analysis of the impact of aspirin on cancer exists yet, strong evidence suggests a causal reduction of the incidence of several cancers due to aspirin therapy. Table B in S2 File weights best and conservative estimates of risk ratios for several cancers’ incidence published in a recent review of existing clinical trials, cohort studies, and case-control studies (Cuzick *et al*., 2014) against the relative incidence of these cancers in the Health and Retirement Study. The factor shown in the table corresponds to the conservative estimates.

We also take into account the direct purchasing cost of aspirin. As of this writing, the estimated cost of daily low-dose aspirin would range from less than $5 to about $20 per person per year, depending on seller and brand. In our simulation, we opt for a measure of $7.29, based on a unit cost of $0.019 per 81 mg tablet—which corresponds to the 500-count package for Walgreens brand aspirin on drugstore.com (accessed Mar. 2, 2015). The direct cost of aspirin is minimal in comparison with its health impact and health-care spending consequences.

#### 2.2.4 Uncertainty

To account for documented uncertainty in aspirin’s health impact, we sampled estimates of its clinical effect from the confidence intervals for relative risks reported in the literature. This random sampling excludes cancer factors, for which Cuzick et al. did not produce confidence intervals. We drew 200 sets of risk-ratio estimates from a log-normal distribution and conducted separate simulations for each. We then computed and sorted the 200 simulation results for all variables of interest. The point estimates of our results correspond to the mean of each variable of interest across the 200. The bounds of the 95% confidence intervals correspond to the fifth lowest and highest results for each variable of interest. These intervals can be interpreted as simulated 95% confidence intervals regarding clinical uncertainty of aspirin’s effectiveness.

## 3. Results

### 3.1 Effects of increased aspirin use on life expectancy, quality of life, and functional status

Our cohort simulations revealed that increased aspirin use would significantly impact disease incidence, life expectancy, and quality of life. Table 2 summarizes how the Guideline Adherence and Universal Eligibility scenarios would affect key health indicators of the life course of nationally representative 51-year-olds in comparison to current aspirin use (Status Quo scenario).

**Table 2:** Increased Aspirin Use Would Prevent Heart Disease and Extend Life.

	Status Quo	Guideline Adherence*			Universal Eligibility**		
	Mean	Mean	Difference with Status Quo [95% CI]		Mean	Difference with Status Quo [95% CI]	
Panel A. Cumulative disease incidence at age 79 (per-thousand)							
Cardiovascular disease	487	476	-11.0	[-23.2 to -2]	469	-17.7	[-34.3 to -3.6]
Stroke	235	233	-2.2	[-11.7 to 7.8]	230	-5.3	[-16 to 6.7]
Cancer	293	290	-3.7	[-10.4 to 1.7]	288	-5.9	[-12.5 to -0.2]
Gastrointestinal bleeding	67	83	16.0	[3.6 to 30]	90	23.7	[6.5 to 42]
Panel B. Expected outcomes at age 51							
Life expectancy (years)	30.2	30.5	0.28	[0.08 to 0.5]	30.6	0.38	[0.14 to 0.65]
Disability-free life-years	22.8	22.9	0.12	[0.03 to 0.23]	23.0	0.18	[0.06 to 0.31]
Quality-adjusted life-years	24.8	25.0	0.20	[0.07 to 0.35]	25.1	0.28	[0.11 to 0.47]

*: Individuals follow 2009 USPSTF guidelines for primary prevention of heart diseases and stroke until age 79 and use aspirin for secondary prevention at all ages

**: All individuals over age 50 are assigned to use aspirin daily.

Panel A shows cumulative incident cases predicted by FEM for 1,000 individuals without prior cardiovascular disease, stroke, or cancer, ages 51–79. Age 79 is relevant because it is the last age individuals were eligible to use aspirin for primary prevention of cardiovascular disease in 2011–2012. After age 79, aspirin use drops in the Guideline Adherence scenario and disease prevalence and mortality catch up relative to Status Quo. Results at age 79 thus reveal the maximum impact of aspirin on disease prevention.

Panel B shows expected outcomes calculated from age 51 until death. Disability-free life expectancy refers to reporting no instrumental activity of daily living or activity of daily living limitations and not living in a nursing home. Quality-adjusted life-years adjust length of life for quality based on a person’s chronic conditions and functional status. 95% confidence intervals with regard to the uncertainty of the effectiveness of aspirin are presented in brackets.

We estimated that guideline adherence would significantly reduce cardiovascular disease. However, because of the differential effect of aspirin on ischemic and hemorrhagic stroke, adherence would not produce significant reductions in incident strokes. Because some individuals with extended life will develop cancer, we also did not find significant reduction in incident cancers despite imposing a modest cancer risk reduction. We predicted a 25% increase in gastrointestinal bleeds relative to Status Quo, which means that one additional American in 63 could expect to suffer a bleeding between ages 51 and 79.

**Fig 2:**
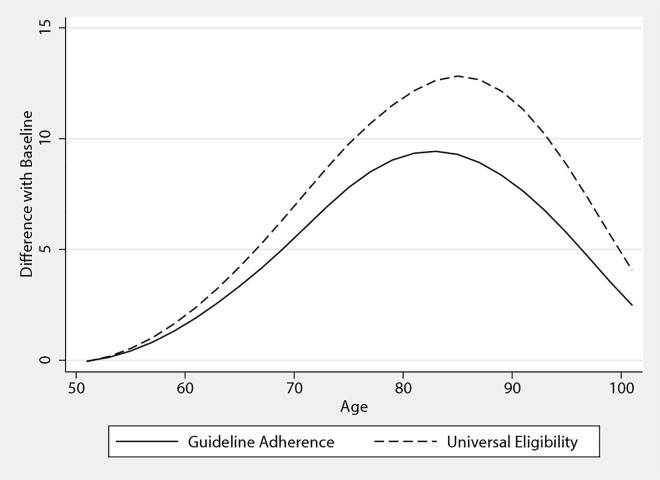
Lives Saved in Increased Aspirin-Use Scenarios Relative to the Status Quo. Difference in mortality of Guideline Adherence and Universal Eligibility scenarios with the Status Quo scenario for a thousand people representative of the American population at age 51. Confidence intervals are omitted for clarity.

Fig 2 shows how the health impact of increased aspirin use would gradually alter the mortality profile of a nationally representative cohort from age 51 until death. Out of 1,000 people, we found that increased aspirin use would mean that eight more Americans would reach age 80, and three would reach 100. As shown in Table 2, this mortality decline would translate into gains of 0.28 years in life expectancy, of which a third would be lived free of disability. When valuing both the health benefits and additional gastrointestinal-bleeding risks, FEM indicated that observing guidelines would increase expected quality-adjusted life expectancy by a fifth of a year. Effects vary across individuals. Younger men who are at high risk of heart disease and younger women at high risk of stroke stood to benefit the most from adherence with the guidelines, as shown in Table C in S2 File.

We presented the results of Universal Eligibility to serve as the upper bound of gains that could be obtained with preventive daily aspirin. This optimistic scenario assumes that the benefits of aspirin stemming from randomized clinical trials could be extended to all older Americans. This scenario produced slightly larger health gains than Guideline Adherence. Life-expectancy gains, for instance, were 0.1 year higher. This suggests that meeting the guidelines would capture most of aspirin’s potential health benefits.

### 3.2 Cost-effectiveness of increased aspirin use

In Table 3, we used FEM projections and reasonable cost estimates of gastrointestinal bleeding and aspirin to value the health benefits of Table 2. Both benefits and costs are discounted at a 3% rate applied from age 51. Following the example of a World Bank report, we assigned a value to health benefits of $150,000 per quality-adjusted life-year, roughly three times the US GDP per capita[23]. We estimated that the overall health effects of guideline compliance from age 51 onward—taking additional gastrointestinal bleeds into account—would be worth, on average, $14,200.

**Table 3:** The Net Benefits of Increased Aspirin Use Would Be Substantial ($2015 thousands).

	Difference with Baseline			
	Guideline Adherence*		Universal Eligibility**	
	Mean	95% CI	Mean	95% CI
Value of expected quality-adjusted life-years gained	14.2	[4.71 to 25.13]	19.9	[7.46 to 34.23]
Expected health-care and medication costs				
Health care excluding gastrointestinal bleeds	5.5	[0.36 to 11.64]	6.6	[0.47 to 12.3]
Gastrointestinal bleeds	0.1	[0.02 to 0.13]	0.1	[0.03 to 0.2]
Aspirin medication	0.1	[0.06 to 0.06]	0.1	[0.1 to 0.1]
Total	5.7	[0.51 to 11.77]	6.8	[0.69 to 12.54]
Net value per capita	8.5	[3.06 to 16.64]	13.0	[5.74 to 22.65]
Incremental cost-effectiveness ratio	64.2	[13.98 to 112.93]	55.3	[13.76 to 91.05]

*: Individuals follow 2009 USPSTF guidelines for primary prevention of heart diseases and stroke until age 79 and use aspirin for secondary prevention at all ages

**: All individuals over age 50 are assigned to use aspirin daily. All amounts are in present value at age 51, computed with a 3% discount rate. Quality-adjusted life-years adjust length of life for quality based on a person’s chronic conditions and functional status. 95% confidence intervals with regard to the uncertainty of the effectiveness of aspirin are presented in brackets.

Expected costs associated with these gains were estimated at $5,500. The majority of additional costs resulted from life-expectancy gains of aspirin, especially additional time lived with a disability or in a nursing home. Thus, the main cost of increasing aspirin use would likely be its very success at preventing disease and extending life. The resulting incremental cost-effectiveness ratio indicates that observing the guidelines would on average cost $64,200 per additional discounted quality-adjusted life-year gained. Despite higher medical costs, the net value of the scenario was positive and significant, with a point estimate of $8,500 at age 51.

The Universal Eligibility scenario produced an upper-bound value of increasing aspirin use of $13,000 per American. As with health gains, this suggests that guideline adherence would provide Americans with most of the possible net gains of aspirin. Table D in S2 File indicated that high-risk Americans, who stand to accrue the most health benefits, would also spend more on health care.

### 3.3 Long-term population implications

**Fig 3:**
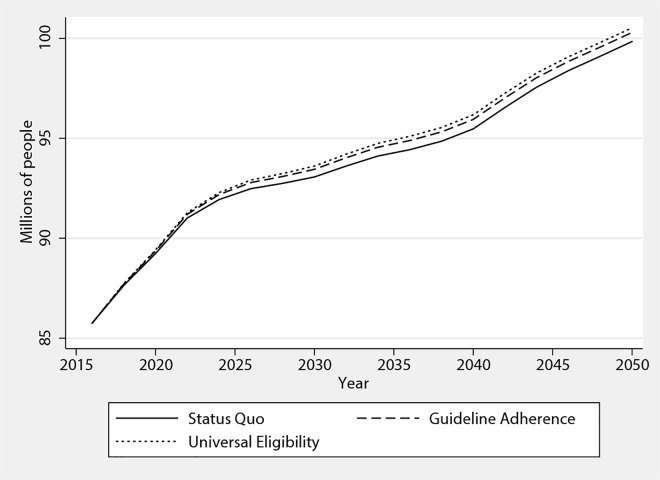
Increasing Aspirin Use for 20 Years Would Add 450,000 People to the Nondisabled Population. Nondisabled population aged over 51. Nondisabled refers to reporting no instrumental activity of daily living or activity of daily living limitations and not living in a nursing home. Confidence intervals are omitted for clarity.

Population-wide simulations help grasp the long-run aggregate implications of the US scenarios. We applied our two extended aspirin use scenarios to the American population for 2016–2050. Notably, these simulations revealed how full guideline implementation would gradually impact population size. In Fig 3, this is shown graphically for the nondisabled American population, which would not see the full effect of adherence until 2036. By that date, we estimated that guideline adherence would result in 460,000 more nondisabled older Americans alive (95% CI 200,000–700,000). Including the disabled population, we estimated that 900,000 lives would be saved because of increased aspirin use (95% CI 300,000–1,400,000) relative to Status Quo.

This additional population would translate into additional annual health-care spending, but not significantly so in the medium term. Annual spending was projected to increase $29.0 billion by 2036 (95% CI –3.0–62.7). By 2050, $63.9 billion in additional total expenditures (95% CI 5.4–123.3) were projected.

Taking into account the quality-adjusted life-years gained, medical spending and gastrointestinal bleeds, we estimated that increased aspirin use would generate $692 billion (95% CI 345–975) in net benefits for the US population over the 2016–2036 period.

### 3.4 Sensitivity analysis

To investigate the sensitivity of our results, we implemented a “worst-case” version of the Guideline Adherence and Universal Eligibility scenarios, in which we consider alternative assumptions likely to lessen the value of implementing the guidelines, departing from the previous scenarios in four ways: 1) Aspirin is assumed to have no impact on cancer incidence; 2) Aspirin is assumed to have no impact on all-cause mortality in primary prevention; 3) Individuals need to take a higher-dose daily aspirin tablet to obtain health benefits, and thus face higher costs and bleed risks; and 4) Direct costs of aspirin tablets are those of brand-name drugs rather than generics (see S3 File). We found that guideline adherence would produce a positive level net value per capita despite the more pessimistic parameters, albeit significant at the 10% confidence level.

## 4. Discussion

As the US works to advance the triple aim of better care, better health, and smarter spending, ensuring patients receive effective preventive care will be critical. Given aspirin’s remarkable preventive effectiveness, it is a rare example of a technology that may produce less disease and better long-term health outcomes for Americans at a low price.

Based on randomized clinical trials on the effectiveness of aspirin for cardiovascular disease prevention and new evidence on cancer, we estimated that observing the guidelines would prevent 11 cases of heart disease and four cancers for every 1,000 Americans aged 51–79 and improve life expectancy by 0.3 years. By 2036, an estimated 900,000 more Americans would be alive if aspirin guidelines were observed. However, according to FEM simulations, about two-thirds of the life-expectancy gains would be lived with a disability, and aspirin’s success in extending life would ultimately increase lifetime medical spending. Despite these higher medical costs, observing the guidelines would yield positive and significant net value.

This study has several potential limitations. Simulations are based on the premise that aspirin’s health effects, as reported by randomized clinical trials literature, can be generalized to all users, though real-world effects are bound to differ. Also, like other effective treatments [24], aspirin may reduce the incentive for other preventive behavior.

More generally, this article provides an illustration of a key challenge of preventive medicine. A *New York Times* blog post recently warned that 2,000 people need to take daily aspirin to prevent a single heart attack over a two-year period, while stressing that “for 1,999 of the 2,000 people, aspirin doesn’t make any difference at all”[25]. Such framing, especially over the near term, is unfavorable to preventive care and favorable to curative care. Indeed, a high number of people must receive preventive care to save one life, while the associated risks may be understandably worrisome. Over the long term, however, preventive care can save millions of lives. The present study, while demonstrating the marked positive impact of aspirin compliance, also emphasizes the need for definitive studies to validate outcomes, as well as the need to develop improved and safer therapeutic alternatives for risk prevention.

Risk prevention for the elderly is critical to achieving the health benefits required for optimal long-term economic and population health. Expanded use of aspirin by older Americans with elevated risk of cardiovascular disease could generate substantial population health benefits over the next twenty years and do so very cost-effectively.

## Supporting Information

S1 File Technical Documentation. (PDF)

S2 File Tables. Table A. Health Impact of Aspirin Reported by Large Meta-Analyses and Table B. Potential for Aspirin to Reduce Cancer Incidence in the Population Aged over 50 and Table C. Health Impact of Guideline Adherence for High-Risk Population and Table D. Costs and Benefits of Guideline Adherence for High-Risk Population, $2015 Thousands and Table E. Sensitivity Analysis: Health Impact of Increased Aspirin Use under Pessimistic Assumptions
(PDF)

S3 File Sensitivity Analyses. A. Sensitivity analysis: health impact and cost-effectiveness of increased aspirin use under pessimistic assumptions and B. Sensitivity analysis: health impact of increased aspirin use under alternative cancer parameters
(PDF)

## Abbreviations

FEM: Future Elderly Model. A dynamic microsimulation that follows Americans aged 50 and older and projects their health and medical spending over time.
NHANES: National Health and Nutrition Examination Survey
