# Supplementary material for: The Long-Term Benefits of Increased Aspirin Use by At-Risk Americans Aged 50 and Older
Source: PLoS One. 2016 Nov 30;11(11):e0166103. doi: 10.1371/journal.pone.0166103 (PMC5130201; doi:10.1371/journal.pone.0166103)
Supplement: S1 File — (PDF) [file pone.0166103.s001.pdf]

The long-term benefits of increased aspirin use by at-risk Americans aged 50 and older

Technical Documentation

Section 1:
Étienne Gaudette, University of Southern California

Sections 2 to 8:
Dana P. Goldman, University of Southern California
Darius Lakdawalla, University of Southern California
Pierre-Carl Michaud, RAND Corporation
Christine Eibner, RAND Corporation
Yuhui Zheng, National Bureau of Economic Research
Adam Gailey, RAND Corporation
Igor Vaynman, RAND Corporation
Jeffrey Sullivan, Precision Health Economics, LLC

September 2016

Table of Contents

TABLE OF CONTENTS **2**

INTRODUCTION **3**

1 SIMULATING THE IMPACT OF EXTENDED ASPIRIN USE **3**

1.1 SIMULATIONS OVERVIEW 6
1.2 ELIGIBILITY 7
1.3 HEALTH IMPACT AND COSTS OF ASPIRIN 11
1.4 UNCERTAINTY 15

2 FUNCTIONING OF THE DYNAMIC MODEL **16**

2.1 BACKGROUND 16
2.2 OVERVIEW 16
2.3 COMPARISON WITH OTHER PROMINENT MICROSIMULATION MODELS 17

3 DATA SOURCES FOR ESTIMATION **18**

3.1 HEALTH AND RETIREMENT STUDY 19
3.2 SOCIAL SECURITY COVERED EARNINGS FILES 19
3.3 NATIONAL HEALTH INTERVIEW SURVEY (NHIS) 20
3.4 THE MEDICAL EXPENDITURE PANEL SURVEY (MEPS) 20
3.5 MEDICARE CURRENT BENEFICIARY SURVEY (MCBS) 20

4 DATA SOURCES FOR TRENDS AND BASELINE SCENARIO **21**

4.1 DATA FOR TRENDS IN ENTERING COHORTS 21
4.2 DATA FOR OTHER PROJECTIONS 21
4.3 DEMOGRAPHIC ADJUSTMENTS 22

5 ESTIMATION **22**

5.1 TRANSITION MODEL 22
5.2 GOODNESS-OF-FIT 26
5.3 QUALITY ADJUSTED LIFE YEARS 26

6 MODEL FOR NEW COHORTS **26**

6.1 INFORMATION AVAILABLE AND EMPIRICAL STRATEGY 26
6.2 MODEL AND ESTIMATION 27

7 GOVERNMENT REVENUE AND EXPENDITURES **30**

7.1 SOCIAL SECURITY BENEFITS 30
7.2 DISABILITY INSURANCE BENEFITS 30
7.3 SUPPLEMENTAL SECURITY INCOME BENEFITS 31
7.4 MEDICAL COSTS ESTIMATION 31
7.5 TAXES 33

8 IMPLEMENTATION OF THE FEM **33**

REFERENCES **34**

TECHNICAL TABLES **40**

Introduction

The first section of this document applies specifically to the article "The long-term benefits of increased aspirin use by at-risk Americans aged 50 and older". It expands upon the Methods section of the article in greater detail. The remainder of the document consists of a detailed technical appendix for the Future Elderly Model, the engine used to conduct simulations.

1 Simulating the impact of extended aspirin use

We conduct simulations using the Future Elderly Model (FEM), a dynamic microsimulation model developed by Goldman *et al.* (2004) to forecast the implications of different medical technology scenarios on long-term health and health care spending. The FEM follows Americans aged 51 and older and projects their health and medical spending over time. Its unique feature is to follow the evolution of individual-level health trajectories, rather than the average or aggregate health characteristics of a cohort. In the recent past, researchers have used it for a variety of purposes, including forecasting the changing health status of the elderly Medicare population in the decades 2010-2030 (Gaudette, Tysinger, Cassil, & Goldman, 2015); estimating the impact of the introduction of statin medication on the costs of obesity (Gaudette, Goldman, Messali, & Sood, 2015); and estimating the value of medical interventions to reduce obesity prevalence (P. C. Michaud, Goldman, Lakdawalla, Zheng, & Gailey, 2012); to delay Alzheimer's disease (Zissimopoulos, Crimmins, & St.Clair, 2015); and to delay the biology of aging (D. P. Goldman et al., 2013; Dana P. Goldman, Gaudette, & Cheng, 2016).

The FEM simulates the lives of older Americans using the Health and Retirement Study, a biennial survey of the American population aged 51 and over that has been ongoing since 1992. The Health and Retirement Study data are used to compute the health transition models at the core of the FEM and the input population that goes into the simulations. Health and Retirement Study data are supplemented by the Medical Expenditure Panel Survey, a set of large-scale surveys of the non-institutionalized U.S. population, and the Medicare Current Beneficiary Survey, a nationally representative survey of Medicare beneficiaries, to project health care spending and assess quality of life during the simulations. For each individual, the FEM takes into account initial demographic characteristics and health conditions to project medical spending, health conditions and behaviors, disability status, and quality of life. We describe the model and methods briefly here; details on the modeling are presented in Sections 2 to 8.

The FEM has three core modules, as illustrated in Figure 1. The first module is the Health Transitions module that calculates transition probabilities across various health states based on the individual's current characteristics. Health transitions include chronic disease incidence, functional status, body-mass index, and mortality. These transition probabilities are modeled using first-order Markov processes that depend on a battery of predictors: age, sex, education, race, ethnicity, body-mass index, smoking behavior, marital status, employment, functional status, and health conditions. We also control for baseline factors using a series of initial health variables.

Figure 1 Structure of the Future Elderly Model

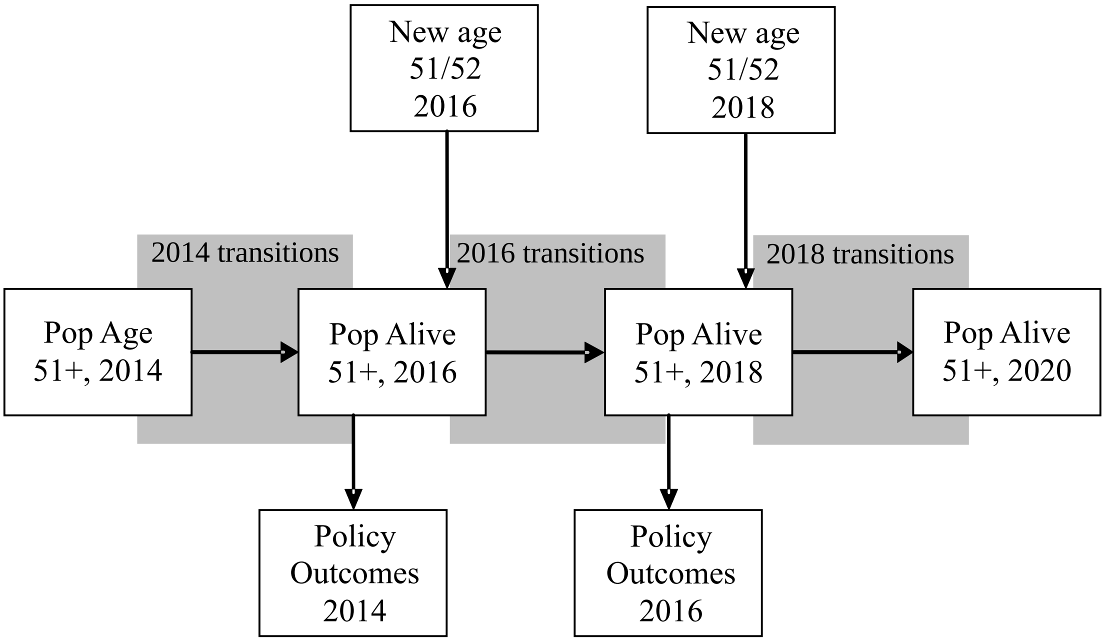

Health conditions are derived from Health and Retirement Study survey questions and include diabetes, high blood pressure, heart disease, cancer (except skin cancer), stroke or transient ischemic attack, and lung disease (either or both chronic bronchitis and emphysema). Transitions into illness and death are synthesized in Figure 2. The concept of chronic conditions used in the simulations corresponds to having *ever* been diagnosed with a condition. We thus treat chronic conditions as absorbing: once individuals receive a diagnosis, they are henceforth considered to have that condition.[1] The body-mass index variable is based on the self-reported height and weight of Health and Retirement Study respondents, and its evolution is projected with the estimates of a log-linear model. Functional status is measured by limitations in instrumental activities of daily living or activities of daily living, and residence in a nursing home. The instrumental activities of daily living limitations indicator is based on questions about difficulty using the phone, managing money, and taking medications. The activities of daily living limitations indicator is based on respondents' assessment of their ability to conduct basic tasks, such as dressing, eating, and bathing. For the purpose of this study, we consider individuals free of disability if they reported no limitations and did not live in a nursing home, and as disabled if they reported at least one limitation or lived in a nursing home. Unlike health conditions, we allow for transitions in and out of functional states.

[1] This interpretation is consistent with the Health and Retirement Study questionnaire, which asks respondents if they were ever diagnosed with a condition.

Figure 2 Chronic Conditions Transitions in the FEM

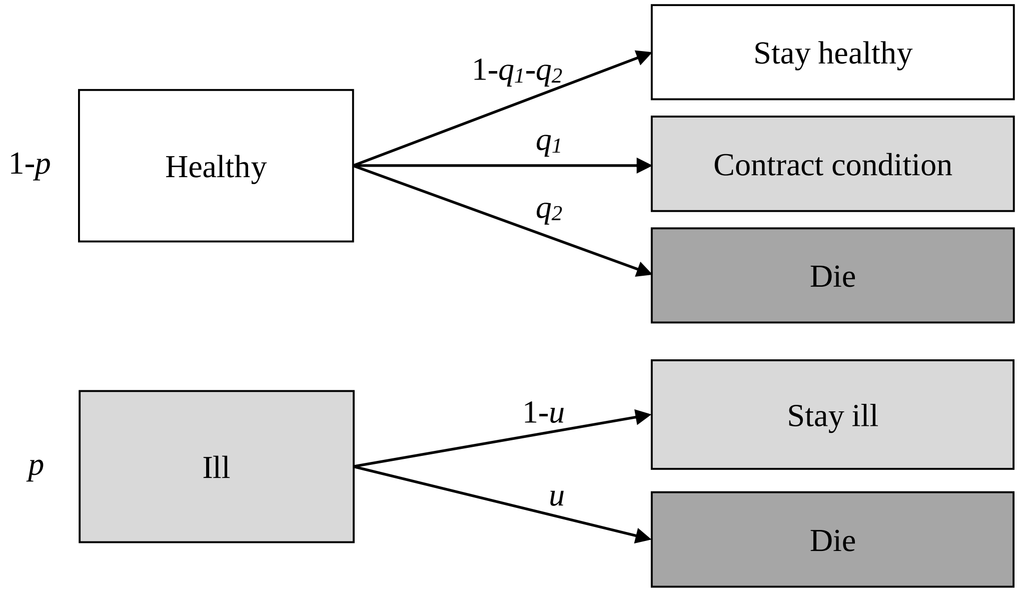

To evaluate quality of life, we predict quality-adjusted life-years using the EQ-5D, a commonly used quality-of-life index based on five health-related variables addressing mobility, daily activities, self-care, anxiety, depression, and pain. Using the Medical Expenditure Panel Survey data, we apply an ordinary least squares regression to fit derived EQ-5D quality adjustment scores as a function of the chronic conditions and functional states included in the FEM simulations. This model is then used to predict the quality of each person's life-years in our simulations.

Based on two complementary medical spending data sources, the Policy Outcomes module predicts an individual's health spending with regards to health status (chronic conditions and functional status), demographics (age, sex, race, ethnicity, and education), nursing home status, and mortality. Our definition of medical spending includes medical provider visits, hospital events, inpatient stays, outpatient visits, emergency department visits, dental care, home health care, optometry, other medical equipment and services, prescribed medicines, and nursing home stay. Our estimates are based on spending data from the 2007-2010 Medical Expenditure Panel Survey for individuals younger than 65 and the 2007-2010 Medicare Current Beneficiary Survey for individuals aged 65 and older. The estimates are based on pooled least squares regressions of each type of spending on risk factors, self-reported conditions, and functional status, with spending inflated to current dollars using the medical component of the consumer price index.

Finally, the Replenishing Cohorts module, shown at the top of Figure 1, predicts economic and health outcomes of new cohorts of 51-year-olds. This module primarily uses data from the Health and Retirement Study and incorporates trends in demography, disease, body-mass index, smoking, and pensions from the National Health and Nutrition Examination Survey (NHANES), the National Health Interview Survey, and the American Community Survey. This module generates cohorts as the simulation proceeds, so that we can measure outcomes for the age 51-plus population in any given year.

1.1 Simulations overview

In this study, we conduct two types of simulations. The first type is cohort simulations, in which we turn off the Replenishing Cohorts module and follow cohorts of Americans until death under alternative aspirin take-up scenarios. These simulations focus on a representative cohort of Americans aged 51 to 52 in 2010. In each period,[2] the Policy Outcomes module predicts medical expenditure on the person's current-state vector. Then, the Health Transitions module predicts survival, health transitions, gastrointestinal bleeding, functional status, and quality-adjusted life-years for the next period, using the FEM's transition probabilities. The same process is repeated at each time step until everyone in the cohort has died. These simulations are useful to compare expected lifetime outcomes across scenarios, such as the probability of contracting a disease by a given age, life expectancy, and lifetime medical spending.

The second type consists of population simulations, in which we use the full FEM structure to project outcomes for the entire American population aged 51 and older until year 2050. In each period, individual outcomes are aggregated to reflect population health measures, such as disease prevalence and aggregate medical costs.

Scenarios

We consider two main scenarios:

- The **Guideline Adherence** scenario provides the health benefits and side effects of aspirin to all individuals for whom aspirin is recommended by the US Preventive Services Task Force and American Heart Association guidelines. We consider the guidelines that were effective at the time of the 2011-2012 NHANES survey on aspirin use. With regard to primary prevention therapy, these guidelines specify a series of 10-year of coronary heart disease and stroke risk thresholds over which men and women are eligible for daily aspirin use as a primary prevention therapy (US Preventive Services Task Force, 2009). With regard to secondary prevention therapy, this scenario considers individuals with a prior stroke or cardiovascular disease as eligible for daily aspirin, as specified by the American Heart Association and American College of Cardiology Foundation guidelines (Smith et al., 2011).[3] Comparisons between the baseline FEM projections (Status Quo scenario) and this scenario will illustrate the full potential of the guidelines, as well as the foregone benefits because of low aspirin use.
- The **Universal Eligibility** scenario provides the health effects of aspirin to all Americans aged 51 and older. This scenario aims to provide an *upper bound* of the impact of additional aspirin use for the U.S. elderly, rather than a realistic

[2] Since the Health and Retirement Study is biennial, we simulate health and costs over two-year periods.

[3] Guidelines for secondary prevention therapy and risk reduction produced by the American Heart Association and American College of Cardiology Foundation broadly define secondary prevention patients as "patients with established coronary and other atherosclerotic vascular disease, including peripheral artery disease, atherosclerotic aortic disease, and carotid artery disease." The most recent update to the secondary prevention guidelines specifically recommends using aspirin 75-162 mg daily in all patients with coronary artery disease unless contraindicated, as well as Aspirin alone (75-325 mg daily), clopidogrel alone (75 mg daily), or combination aspirin plus extended-release dipyridamole in all patients with extracranial or vertebral atherosclerosis who have had ischemic stroke or transient ischemic attack.

assessment of the impact that would occur if everyone aged 51 and older used aspirin daily.

1.2 Eligibility

Scenarios are implemented in two steps. First, we identify the individuals affected by the scenario. Since a subset of the population eligible for aspirin already takes the medication, we identify in FEM simulations individuals admissible for aspirin use under each scenario but *not* using aspirin. These are the individuals who would see their life outcomes change if the guidelines were fully observed or if all Americans aged 51 and older started taking aspirin. Since there is no Health and Retirement Study question about aspirin use, we turn to NHANES data, which contain detailed information about clinical bio-markers and aspirin use.

The NHANES data reveal a large unmet need for daily aspirin. In the article's Figure 1, we compare the population eligible for aspirin in a primary or secondary prevention setting against its reported aspirin use. Primary prevention eligibility, which corresponds to 10-year risks of coronary heart disease and stroke above the thresholds of the guidelines, is shown in yellow; secondary prevention eligibility, which corresponds to the population with a prior stroke or heart disease, is shown in blue. The darker section of the bars shows the proportion of the eligible population that also reported using daily aspirin.

Strikingly, most men aged 50 to 69 presented coronary heart disease risks above the risks for which aspirin use was recommended, but less than 20% were following the guidelines (in pale yellow). Fewer women presented a risk of stroke above the thresholds recommended for aspirin use, but over 10% of women in all age groups over 55 were eligible for aspirin use but not taking it. The data used to estimate this population and the process to identify it in FEM simulations are detailed in following section.

1.2.1 Data

To estimate the probability that individuals are eligible for aspirin use within FEM simulations, we utilize estimates based on the National Health and Nutrition Examination Survey (NHANES) data. NHANES is a set of studies combining interviews and physical examinations to assess the health and nutritional status of adults and children in the United States.

In this study, we assemble and use several components of NHANES to assign aspirin eligibility. Demographic files and medical conditions files are used to extract characteristics of respondents common to both NHANES and the FEM (which is based on the Health and Retirement Study). Second, information from biomarker measurements¬—namely cholesterol and blood pressure—are used to calculate 10-year risks of coronary heart disease and stroke. Comparing these risks against the US Preventive Services Task Force's (USPSTF) 2009 guidelines' thresholds provides the eligibility for aspirin use. Finally, this study benefits from a special survey on the preventive use of low-dose aspirin conducted in 2011-2012.

By bringing in components together for years 2007-2012, we obtained an individual-level database including individual characteristics, risk of cardiovascular events, and daily aspirin use in 2011-2012. A NHANES respondent is defined as an aspirin We define as an aspirin user a respondent of the NHANES 2011-2012 preventive

aspirin use questionnaire if he or she self-reported taking daily low-dose aspirin as advised by a doctor of other health care provider; or if he or she self-reported as taking daily low-dose aspirin on his or her own.

Under the Guideline Adherence scenario that we implement in FEM, we define the eligibility for aspirin use in a primary prevention context as the intersection of 1) not using low-dose aspirin, and 2) displaying a risk for coronary heart disease and stroke events above the thresholds of the 2009 USPSTF guidelines. Since both outcomes are dichotomous, we conduct probit regressions on our NHANES dataset to find the probability that individuals are eligible at each period in the FEM simulations.

1.2.2 Estimation

The first regression finds the factors influencing the probability that individuals aged 51 and over (the population covered by the FEM) declare using daily low-dose aspirin, based on information common to NHANES and the FEM: demographics, education, health conditions and body-mass index. The second sets of regressions find the factors influencing the probability of women (men) having a risk of stroke (coronary heart disease) above the USPSTF thresholds, respectively. Since the guidelines concern the primary prevention of cardiovascular diseases and target men aged 45-79 and women aged 55-79, we apply the same restrictions to these regressions' samples. Additionally, the USPSTF guidelines statement specifies that all diabetic men are eligible for daily aspirin use, therefore they are excluded from the regression.

After several specification checks, we used age splines with nodes at age 59 and 69 in the models for aspirin use and eligibility for primary prevention aspirin use in men to maximize fit. For women age and age-squared variables produced a better fit than age splines and were favored. The results of these regressions are presented in tables 1 and 2.

1.2.3 Assignment and validation

The use of these estimates follows the same methodology as the other non-absorbing binary outcomes in the FEM. At the end of each two-year period, we assign aspirin use and Guideline Adherence eligibility to each simulated individuals based on their characteristics and the models presented in tables 1 and 2. In the Guideline Adherence scenario, individuals for whom the simulations indicate that they do not currently use aspirin and have a risk superior to the thresholds suggested by the USPSTF are defined as eligible. Additionally, all diabetic men under age 80 are considered eligible. These constitute the population eligible for daily use of aspirin in a primary prevention setting. Additionally, all individuals with a prior diagnosis of stroke or cardiovascular disease are considered eligible for daily aspirin use in a secondary prevention setting.

Thus, NHANES data is used to assign aspirin eligibility to our FEM populations, which are based on Health and Retirement Study data. The two surveys are representative of the American population they study, but whether our assignment of aspirin intervention eligibility will be consistent with observed data is initially unclear. To verify this, we impute eligibility for aspirin as a primary prevention therapy to each individual of the FEM sample used for population simulations. We then compare the population

identified as eligible for an aspirin intervention to its observed counterpart in NHANES in wave 2011-2012 (Table 3).

We find that our imputation models identify a somewhat smaller proportion of the population as eligible for an aspirin intervention relative to the NHANES data. Also, the population identified by the imputation presents superior health indicators: it is less likely to be obese, currently smoking, or hypertensive. These discrepancies suggest that the imputation leads to conservative estimates of the true population that could benefit from aspirin therapy and of the health benefits they obtain from the intervention.

Table 1 Factors Influencing the Probability of Declaring Using Aspirin between Ages 51 and 79 in NHANES, 2011-2012: Probit Estimates

	Coefficient		Standard error	Marginal effect
Demographics and education				
Black	0.01		(0.93)	0.003
Hispanic	-0.06		(0.61)	-0.023
Male	0.03		(0.83)	0.010
Less than high school	0.18	*	(0.09)	0.068
College education	0.27	*	(0.06)	0.101
Male and less than high school	0.03		(0.88)	0.010
Male and college	-0.10		(0.53)	-0.035
Male and black	-0.10		(0.47)	-0.036
Male and Hispanic	-0.22		(0.18)	-0.080
Health Conditions				
Cancer	0.09		(0.27)	0.036
Diabetes	0.39	***	(0.00)	0.151
Heart diseases	0.71	***	(0.00)	0.276
High blood pressure	0.49	***	(0.00)	0.181
Lung diseases	-0.30	*	(0.07)	-0.106
Stroke	0.23	*	(0.05)	0.089
Weight (BMI status dummies)				
BMI in [30.35]	0.10		(0.18)	0.037
BMI in [35,40]	0.15		(0.15)	0.057
BMI ≥ 40	0.13		(0.28)	0.048
Age (linear splines)				
Less than 59 years old	0.06	***	(0.00)	0.023
Between 59 and 69	0.00		(0.83)	-0.001
over 69 years old	0.03	**	(0.02)	0.013
Constant	-4.61	***	(0.00)	
Observations	2,132			
Partial R-squared	0.126			

*** p<0.01, ** p<0.05, * p<0.1. BMI refers to “body mass index”, defined as the ratio between mass of individuals, expressed in kilograms, and the square of height, expressed in meters. A respondent of the NHANES 2011-2012 preventive aspirin use questionnaire respondent is defined as an aspirin user if he or she self-reported taking daily low-dose aspirin as advised by a doctor of other health care provider; or if he or she self-reported as taking daily low-dose aspirin on his or her own.

Table 2 Factors Influencing the Probability of Eligibility for Primary Prevention Aspirin Use in NHANES, 2007-2012: Probit Estimates

	1. Women aged 55-79 without prior diagnosis of heart disease or stroke				2. Men aged 51-79 without prior diagnosis of heart disease, stroke or diabetes			
	Coefficient		Standard error	Marginal effect	Coefficient		Standard error	Marginal effect
Demographics and education								
Black	0.16	**	(0.00)	0.055	-0.15	*	(0.08)	-0.033
Hispanic	0.12		(0.12)	0.040	0.14		(0.13)	0.029
Less than high school	0.00		(0.98)	-0.001	0.10		(0.31)	0.021
College education	-0.15	**	(0.04)	-0.050	-0.17	**	(0.04)	-0.037
Health Conditions								
Cancer	-0.08		(0.34)	-0.027	-0.06		(0.56)	-0.012
Diabetes	0.86	***	(0.00)	0.319				
High blood pressure	0.66	***	(0.00)	0.215	0.44	***	(0.00)	0.091
Lung diseases	0.23		(0.20)	0.082	0.01		(0.97)	0.002
Weight (BMI status dummies)								
BMI in [30.35]	-0.08		(0.25)	-0.028	0.15	*	(0.09)	0.030
BMI in [35,40]	-0.13		(0.17)	-0.042	0.20		(0.18)	0.039
BMI ≥ 40	-0.13		(0.22)	-0.042	0.44	*	(0.09)	0.073
Age	-0.80	***	(0.00)	-0.270				
Age squared	0.01	***	(0.00)	0.002				
Age linear splines								
Less than 59 years old					0.01		(0.39)	0.003
Between 59 and 69					-0.07	***	(0.00)	-0.014
over 69 years old					-0.01		(0.39)	-0.003
Constant	24.35	***	(0.00)		0.38		(0.69)	
Observations	2,469				2,258			
Partial R-squared	0.166				0.0703			

*** p<0.01, ** p<0.05, * p<0.1. BMI refers to “body mass index”, defined as the ratio between mass of individuals, expressed in kilograms, and the square of height, expressed in meters. Eligibility for primary prevention daily use of aspirin is assigned to NHANES respondents based on 2009 USPSTF guidelines and established 10-year risk calculators for coronary heart disease and stroke.

Table 3 Comparison of Population Identified as Eligible for Aspirin Intervention in FEM Simulations and NHANES Data

	NHANES (%)	FEM (%)	Difference (%)
Population aged 51+ identified as eligible for aspirin as a primary prevention therapy*	38	34	-4
Using aspirin	10	11	1
Eligible for intervention	28	23	-5
Profile of population eligible for intervention			
Male	82	78	-5
With less than a high school degree	17	17	1
Obese	35	26	-8
Currently smoking	29	21	-9
Hypertensive	45	41	-4
Diabetic	14	14	1

*Eligibility for primary prevention daily use of aspirin is assigned to respondents based on USPSTF thresholds and established 10-year risk calculators for coronary heart disease and stroke and aspirin use. “NHANES” refers to author’s calculations with the 2011-2012 wave of the NHANES. “FEM” refers to the imputation of aspirin eligibility in the Future Elderly Model’s stock population using the models shown in Tables 1 and 2.

1.3 Health Impact and Costs of Aspirin

The second step modifies health transitions and outcomes of eligible individuals to reflect the health impact of aspirin use reported by the clinical trials literature. These include both health benefits of daily aspirin use and its side effects, both of which have been reported by many researchers. Table 4 summarizes the most important findings from recent meta-analyses.

For eligible individuals, we decrease the probabilities of contracting heart disease, experiencing a stroke, and mortality and increase the risks of gastrointestinal bleeding. We incorporate differential effects by sex and context:

1. **Heart disease:** For individuals who have never been diagnosed with a cardiovascular disease (i.e. in a primary prevention setting), we decrease the probability of contracting heart disease by factors with a mean of 0.90. The distribution used corresponds to the risk ratios of total cardiovascular disease events among the primary prevention population reported by Seshasai *et al.* (2012). For individuals with a prior stroke but no history of other heart disease, we decrease the probability of contracting a heart disease by factors with a mean of 0.79, based on Berger *et al.* (2008).
2. **Stroke:** A meta-analysis conducted in 2006 by Berger revealed differential effects of aspirin by stroke type and sex in individuals with no prior cardiovascular disease. For men, this study reported that daily aspirin use does not significantly reduce the risk of ischemic stroke but *increases* the risk of hemorrhagic stroke. For women, however, aspirin reduces the ischemic stroke risk and does not significantly increase hemorrhagic stroke risk. Since the concept of stroke in the Health and Retirement Study includes both types, we implement the total stroke effects reported by the same study: we modify the

overall risk of stroke for men and women by factors with a mean of 1.13 and 0.83, respectively. In a secondary prevention setting, we reduce the risk of stroke of both men and women by factors with a mean of 0.75, based on Berger *et al.* (2008).

3. **Cancer:** While no comprehensive meta-analysis of the impact of aspirin on cancer exists yet, strong evidence suggests a causal reduction of the incidence of several cancers due to aspirin therapy. Table 5 weights best and conservative estimates of risk ratios for several cancers' incidence published in a recent review of existing clinical trials, cohort studies, and case-control studies (Cuzick et al., 2014) against the relative incidence of these cancers in the Health and Retirement Study. We reduce the incidence probability of cancer by 0.94, which corresponds to the conservative estimates.
4. **Mortality:** Depending on whether aspirin is used for primary or secondary prevention, the literature reports differential mortality effects of aspirin. In a primary prevention setting, we modify mortality probability by factors with a mean of 0.94, based on the estimates reported by Seshasai *et al.* (2012).[4] In a secondary prevention setting, we decrease the mortality probability by factors with a mean of 0.87, based on Berger *et al.* (2008). In the simulations, the parameters are adjusted for the indirect impact of aspirin on mortality through its prevention of non-fatal chronic disease incidence, which impact the probability of mortality in subsequent periods. The adjustment is obtained by conducting intermediary cohort simulations in which aspirin is allowed to impact heart disease, stroke, and cancer, but not mortality, and then comparing the ratio of mortality rates in this scenario to the baseline. Specifically, participants of the studies comprising the Berger *et al.* (2008) meta-analysis had a mean age of 63.9 at baseline and were followed for an average of 33 months, over which aspirin use was associated with an all-cause mortality risk ratio point estimate of 0.87. With FEM, we allowed aspirin to impact incidence of chronic disease but not mortality at ages 63 to 67 in a secondary prevention setting. This exercise resulted in an all-cause mortality ratio with the status quo of 0.998 over these ages. We thus applied a reduction of mortality using factors with a mean of 0.87/0.998 = 0.872. Similarly, participants of the studies comprising Seshasai *et al.* (2012) had a mean age of 57 at baseline and were followed for an average of 6 years. Allowing aspirin to impact incidence of chronic disease and mortality in a secondary prevention setting but not of mortality in a primary prevention setting resulted in an all-cause mortality ratio of 0.984 at ages 57 to 63. We thus modify mortality probabilities using factors with a mean of 0.94/0.984 = 0.955.
5. **Gastrointestinal bleeding**: Gastrointestinal bleeding is the most common side effect of daily aspirin use. Based on the estimates of McQuaid and Laine (2006), we increase bleeding risk by factors with a mean of 1.73, which are consistent with low-dose daily use of aspirin (75 to 162.5 mg/day). To introduce the baseline bleeding risks in the FEM to which the factors are

[4] We note that a recent large-scale randomized study conducted in Japan, which has not yet been included in meta-analyses, did not find a reduction in all-cause mortality in a primary care setting (Ikeda et al., 2014). We therefore conduct a sensitivity scenario in which we remove this parameter.

applied, we use the age- and gender-stratified rates of any gastrointestinal bleeding events in the UK general population risks reported by Thorat and Cuzick (2015).[5] Following estimates from the medical literature on bleeds, we impose a utility adjustment of 0.125[6] and a medical cost of $4,639[7] for additional gastrointestinal bleeding incidents incurred because of aspirin use (Campbell et al., 2015; Ghate, Biskupiak, Ye, Kwong, & Brixner, 2011).

[5] The authors estimate that about 10% of gastrointestinal bleeding events in the population were due to the use of nonsteroidal anti-inflammatory drugs, of which aspirin is a prominent member. To correctly reflect the risks of individuals prior to using aspirin, we thus consider .9 times the incidence rates as the baseline risks of gastrointestinal bleeding.

[6] Campbell *et al.* find that upper gastrointestinal bleeding is associated with a mean EQ-5D health-related quality-of-life score among survivors of 0.735 at 28 days, in comparison with a general population score of 0.86. In our simulations, this quality-of-life decrement is applied as a reduction of 0.125 to QALY scores predicted by the FEM in the whole year in which a gastrointestinal bleed occurs.

[7] Ghate *et al.* used generalized linear regression to model health care costs during the 12 months after a warfarin prescription among patients using warfarin to prevent occlusive stroke secondary to atrial fibrillation. They compared health care costs between those patients that had a bleeding event within the first 30 days following their warfarin claim and those that did not. The models adjusted for age, gender, region, insurance plan type, and comorbidity. Assuming that the difference in costs between the "no bleeding" group and the "major GI" group is entirely attributable to the gastrointestinal bleed, then the gastrointestinal bleed resulted in $13,747 in combined inpatient and outpatient costs over 12 months, after adjusting for the previously mentioned factors. Minor gastrointestinal bleeds, which accounted for 66.3% of gastrointestinal bleeding events, did not increase annual costs significantly. The value used in our simulation corresponds to the costs of major gastrointestinal bleeds weighted by their relative occurrence.

Table 4 Health Impact of Aspirin Reported by Large Meta-Analyses

Meta-Analysis	Population (Primary or Secondary CVD Prevention)	Effect of Aspirin Therapy Compared to Placebo
Seshasai *et al.*, 2012	Primary	**Decreased nonfatal MI (RR 0.80 95% CI 0.67-0.96)**<br>Decreased total CVD events (RR 0.90 95% CI 0.85-0.96)<br>Increased total bleeds (RR 1.7 95% CI 1.17-2.46)<br>Increased nontrivial bleeds (RR 1.31 95% CI 1.14-1.5)<br>**Decreased all-cause mortality (RR 0.94 95% CI 0.88-1.00)**
Berger *et al.*, 2011	Primary	Decreased major CVD events (RR 0.9 95% CI 0.85-0.96)<br>Increased hemorrhagic stroke (RR 1.35 95% CI 1.01-1.81)<br>Increased major bleeds (RR 1.62 95% CI 1.31-2)
Raju *et al.*, 2011	Primary	Decreased major CVD events (RR 0.88 95% CI 0.83-0.94)<br>Decreased ischemic stroke (RR 0.86 95% CI 0.75-0.98)<br>Increased hemorrhagic stroke (RR 1.35 95% CI 1.01-0.94)<br>Increased GI bleeds (RR 1.37 95% CI 1.15-1.62)<br>Increased major bleeds (RR 1.66 95% CI 1.41-1.95)
Rothwell *et al.*, 2011	Primary or Secondary	Decreased colorectal cancer mortality (RR 0.6 95% CI 0.43-0.81)<br>Decreased oesophageal cancer mortality (RR 0.42 95% CI 0.25-0.71)<br>Decreased lung cancer mortality (RR 0.71 95% CI 0.58-0.89)<br>Decreased all solid cancer mortality (RR 0.75 95% CI 0.67-0.84)<br>Decreased all cancer mortality (RR 0.78 95% CI 0.7-0.87)
Rothwell *et al.*, 2010	Primary or Secondary	Decreased colorectal cancer incidence after ≥ 2.5 years of aspirin treatment (RR 0.69 95% CI 0.51-0.93), ≥ 5 years of aspirin treatment (RR 0.62 95% CI 0.43-0.94), or an unspecified treatment duration (RR 0.75 95% CI 0.56-0.97)
Baigent *et al.*, 2009(Baigent et al., 2009)	Primary	Decreased major coronary events in men (RR 0.77 95% CI 0.67-0.89)<br>Decreased ischemic stroke in women (RR 0.77 95% CI 0.59-0.99)
	Secondary	Decreased major coronary events in men (RR 0.81 95% CI 0.72-0.92)
Berger *et al.*, 2008	Secondary	**Decreased total CVD events (OR 0.794 95% CI 0.715-0.882)**<br>Decreased total MI (OR 0.738 95% CI 0.598-0.91)<br>**Decreased total stroke (OR 0.754 95% CI 0.654-0.869)**<br>**Decreased all-cause mortality (OR 0.872 95% CI 0.764-0.995)**<br>Increased major bleeding (OR 2.332 95% CI 1.599-3.399)
Berger *et al.*, 2006	Primary	Decreased total CVD events in men (OR 0.86 95% CI 0.78-0.94) and women (OR 0.88 95% CI 0.79-0.99)<br>Decreased total MI in men (OR 0.68 95% CI 0.54-0.86)<br>**Decreased total stroke in women (OR 0.83 95% CI 0.7-0.97)**<br>Decreased ischemic stroke in women (OR 0.76 95% CI 0.63-0.93)<br>**Increased total stroke in men (OR 1.13 95% CI 0.96-1.33)**<br>Increased hemorrhagic stroke in men (OR 1.69 95% CI 1.04-2.73)<br>Increased major bleeding in men (OR 1.72 95% CI 1.35-2.2) and women (OR 1.69 95% CI 01.13-2.52)

Table 4 Health Impact of Aspirin (cont.)

McQuaid and Laine, 2006	Primary or Secondary	**Increased any major bleeding (RR 1.71 95% CI 1.41-2.08)**<br>Increase major GI bleeding (RR 2.07 95% CI 1.61-2.66)<br>Increased intracranial bleeding (RR 1.65 95% CI 1.12-2.44)<br>Increased non-GI, non-intracranial bleeding (RR 1.72 95% CI 1.39-2.13)<br>Increased fatal intracranial bleeding (RR 2.52 95% CI 1.06-5.99)

Findings used in the simulations are shown in bold font. Only statistically significant findings are shown, with the exception of non-statistically significant findings that were included in the simulations. RR - risk ratio, OR - odds ratio, CVD - cardiovascular disease, MI - myocardial infarction, GI – gastrointestinal.

Table 5 Potential for aspirin to reduce cancer incidence in the population aged over 50

Incident Cancer Site	Relative incidence*	Risk Ratio ** Conservative estimate	Best estimate
Prostate	24%	0.95	0.9
Breast	16%	0.95	0.9
Rectum Bowel Colon	13%	0.7	0.65
Bronchia bronchus Lung chest-NFS	8%	1	0.95
Stomach	1%	0.75	0.7
Other	37%	1	1
**Total**	**100%**	**0.94**	**0.91**

*: Corresponds to the share of new incident cancers observed in Health and Retirement Study at ages over 50 (years 1995-2008). "Incident" means that the respondent reported no cancer in the prior wave, but reported cancer in reference wave; **: Site-specific risk ratio estimates of Cuzick *et al*.(2014).

Finally, we take into account the direct purchasing cost of aspirin medication. As mentioned in the introduction, aspirin is quite inexpensive. As of the writing of this article, the estimated cost of providing a patient with a daily low-dose aspirin tablet would range from less than $5 to about $20 per year, depending on the seller and brand. In our simulation, we opt for a measure of $7.29, based on a unit cost of $0.019 per 81mg tablet.[8] As shown in the results section, the direct cost of aspirin is minimal in comparison with its health impact and health care spending consequences.

1.4 Uncertainty

To account for documented uncertainty in the health impact of aspirin, we sampled estimates of its clinical effect from the confidence intervals for relative risks reported by the literature.[9] Assuming parameters to be independent from each other, we drew 200 sets of risk-ratio estimates from a log-normal distribution and conducted separate simulations for each of them. We then computed and sorted the simulation results for all variables of interest. In the remainder of the article, the point estimates of our results correspond to the mean of each variable of interest across the 200 draws. The bounds of the 95%

[8] This cost corresponds to the 500-count package price for Walgreens brand aspirin found on the website www.drugstore.com on March 2, 2015.

[9] This random sampling excludes cancer factors, for which Cuzick *et al.* did not produce confidence intervals.

confidence intervals correspond to the 5th lowest and highest results for each variable of interest. These intervals can be interpreted as simulated 95% confidence intervals with regard to the clinical uncertainty of the effectiveness of aspirin.

2 Functioning of the Dynamic Model

2.1 Background

The Future Elderly Model (FEM) is a microsimulation model originally developed out of an effort to examine health and health care costs among the elderly Medicare population (age 65+). A description of the previous incarnation of the model can be found in Goldman et al. (2004). The original work was founded by the Centers for Medicare and Medicaid Services and carried out by a team of researchers composed of Dana P. Goldman, Paul G. Shekelle, Jayanta Bhattacharya, Michael Hurd, Geoffrey F. Joyce, Darius N. Lakdawalla, Dawn H. Matsui, Sydne J. Newberry, Constantijn W. A. Panis and Baoping Shang.

Since then various extensions have been implemented to the original model. The most recent versions now projects health outcomes for all Americans aged 51 and older and uses the Health and Retirement Study (HRS) as a host dataset rather than the Medicare Current Beneficiary Survey (MCBS). The work has also been extended to include economic outcomes such as earnings, labor force participation and pensions. This work was funded by the National Institute on Aging through its support of the RAND Roybal Center for Health Policy Simulation (P30AG024968), the Department of Labor through contract J-9-P-2-0033, the National Institutes of Aging through the R01 grant “Integrated Retirement Modeling” (R01AG030824） and the MacArthur Foundation Research Network on an Aging Society. Finally, the computer code of the model was transferred from Stata to C++. This report incorporates these new development efforts in the description of the model.

All tables referenced in the following sections are shown at the end of the document.

2.2 Overview

The defining characteristic of the model is the modeling of real rather than synthetic cohorts, all of whom are followed at the individual level. This allows for more heterogeneity in behavior than would be allowed by a cell-based approach. Also, since the HRS interviews both respondent and spouse, we can link records to calculate household-level outcomes such as net income and Social Security retirement benefits, which depend on the outcomes of both spouses. The omission of the population younger than age 51 sacrifices little generality, since the bulk of expenditure on the public

programs we consider occurs after age 50. However, we may fail to capture behavioral responses among the young.

The model has three core components:

• The initial cohort module predicts the economic and health outcomes of new cohorts of 51/52 year-olds. This module takes in data from the Health and Retirement Study (HRS) and trends calculated from other sources. It allows us to "generate" cohorts as the simulation proceeds, so that we can measure outcomes for the age 51+ population in any given year.

• The transition module calculates the probabilities of transiting across various health states and financial outcomes. The module takes as inputs risk factors such as smoking, weight, age and education, along with lagged health and financial states. This allows for a great deal of heterogeneity and fairly general feedback effects. The transition probabilities are estimated from the longitudinal data in the Health and Retirement Study (HRS).

• The policy outcomes module aggregates projections of individual-level outcomes into policy outcomes such as taxes, medical care costs, pension benefits paid, and disability benefits. This component takes account of public and private program rules to the extent allowed by the available outcomes. Because we have access to HRS-linked restricted data from Social Security records and employer pension plans, we are able to realistically model retirement benefit receipt.

Figure 1 provides a schematic overview of the model. We start in 2014 with an initial population aged 51+ taken from the HRS. We then predict outcomes using our estimated transition probabilities (see section 4.1). Those who survive make it to the end of that year, at which point we calculate policy outcomes for the year. We then move to the following time period (two years later), when a new cohort of 51 and 52 year-olds enters (see section 5.1). This entrance forms the new age 51+ population, which then proceeds through the transition model as before. This process is repeated until we reach the final year of the simulation.

2.3 Comparison with Other Prominent Microsimulation Models

The FEM is unique among existing models that make health expenditure projections. It is the only model that projects health trends rather than health expenditures. It is also the only model that generates mortality out of assumptions on health trends rather than historical time series.

2.3.1 CBOLT Model (CBO)

The Congressional Budget Office (CBO) uses time-series techniques to project health expenditure growth in the short term and then makes an assumption on long-term growth.

They use a long term growth of excess costs of 2.3 percentage points starting in 2020 for Medicare. They then assume a reduction in excess cost growth in Medicare of 1.5% through 2083, leaving a rate of 0.9% in 2083. For non-Medicare spending they assume an annual decline of 4.5%, leading to an excess growth rate in 2083 of 0.1%.

2.3.2 Centers for Medicare and Medicaid Services (CMS)

The Centers for Medicare and Medicaid Services (CMS) performs an extrapolation of medical expenditures over the first ten years, then computes a general equilibrium model for years 25 through 75 and linearly interpolates to identify medical expenditures in years 11 through 24 of their estimation. The core assumption they use is that excess growth of health expenditures will be one percentage point higher per year for years 25-75 (that is if nominal GDP growth is 4%, health care expenditure growth will be 5%).

3 Data Sources for Estimation

The Health and Retirement Study is the main data source for the model. We supplemented this data with merged Social Security covered earnings histories and data on health trends and health care costs coming from 3 major health surveys in the U.S. We describe these surveys below and the samples we selected for the analysis. We first list the variables used in the analysis. We then give details on the data sources.

Estimated Outcomes in Initial Conditions Model

**Economic Outcomes**	**Health Outcomes**
Employment	Hypertension
Earnings	Heart Disease
Wealth	Self-Reported Health
Defined Contribution Pension Wealth	BMI Status
Pension Plan Type	Smoking Status
AIME	Functional Status
Social Security Quarters of Coverage	
Health Insurance	

Estimated Outcomes in/from Transition Model

Economic Outcomes	Health Outcomes	Other Outcomes
Employment	Death	Income Tax Revenue
Earnings	Heart	Social Security Revenue
Wealth	Stroke	Medicare Revenue
Demographics	Cancer	Medical Expenses
Health Insurance	Hypertension	Medicare Part A Expenses
Disability Insurance Claim	Diabetes	Medicare Part B Expenses
Defined Benefit Claim	Lung Disease	Social Security Outlays
SSI Claim	Nursing Home	
Social Security Claim	BMI	
	Smoking Status	
	ADL Limitations	
	IADL Limitations	

3.1 Health and Retirement Study

The Health and Retirement Study (HRS), waves 1992-2004 are used to estimate the transition model. Interviews occur every two years. We use the dataset created by RAND (RAND HRS, version K) as our basis for the analysis. We use all cohorts in the analysis and consider sampling weights whenever appropriate. When appropriately weighted, the HRS in 2004 is representative of U.S. households where at least one member is at least 51. The HRS is also used as the host data for the simulation (pop 51+ in 2004) and for new cohorts (aged 51 and 52 in 2004).

The HRS adds new cohorts every six years. Until recently, the latest available cohort had been added in 2004, which is why that is the FEM's base year. The FEM is currently being updated to use the newly released 2010 data.

3.2 Social Security Covered Earnings Files

To get information on Social Security entitlements of respondents, we match the HRS data to the Social Security Covered Earnings files of 1992, 1993, 1998 and 2004 which provides information on earning histories of respondents as well as their entitlement to future Social Security benefits. We then construct the average indexed monthly earnings (AIME), the basis for the determination of benefit levels, from these earning histories. The AIME is constructed by first indexing using the National Wage Index (NWI) to the wage level when the respondent turns age 60. If this occurs after 2008, we project the evolution of the NWI using the average annual rate of change of the last 20 years (2.9% nominal). We then take the 35 highest years (if less than 35 years are available, remaining years are considered zero earning years) and take the average. We then convert back this annual amount on a monthly basis and convert back to $2004 U.S. dollars using the CPI. Quarters of coverage, which determine eligibility to Social Security, are defined as the sum of posted quarters to the file. A worker is eligible to Social Security if he has accumulated at least 40 quarters of coverage. A worker roughly accumulates a quarter of

coverage for every $4000 of coverage earnings up to a maximum of 4 per year. Not all respondents agree to have their record matched. Hence, there is the potential for non-representativeness. However, recent studies show that the extent of non-representativeness is quite small and that appropriate weighting using HRS weights mostly corrects for this problem (P.-C. Michaud, Kapteyn, Smith, & van Soest, 2006).

3.3 National Health Interview Survey (NHIS)

The NHIS contains individual-level data on height, weight, smoking status, self-reported chronic conditions, income, education, and demographic variables. It is a repeated cross-section done every year for several decades. But the survey design has been significantly modified several times. Before year 1997, different subgroups of individuals were asked about different sets of chronic conditions, after year 1997, a selected sub-sample of the adults were asked a complete set of chronic conditions. The survey questions are quite similar to that in HRS. As a result, for projecting the trends of chronic conditions for future 51/52 year-olds, we only use data from 1997 to 2010. A review of survey questions is provided in Table 2. Information on weight and height were asked every year, while information on smoking was asked in selected years before year 1997, and has been asked annually since year 1997.

3.4 The Medical Expenditure Panel Survey (MEPS)

The MEPS, beginning in 1996, is a set of large-scale surveys of families and individuals, their medical providers (doctors, hospitals, pharmacies, etc.), and employers across the United States. The Household Component (HC) of the MEPS provides data from individual households and their members, which is supplemented by data from their medical providers. The Household Component collects data from a representative sub sample of households drawn from the previous year's National Health Interview Survey (NHIS). Since NHIS does not include the institutionalized population, neither does MEPS: this implies that we can only use the MEPS to estimate medical costs for the non-elderly population. Information collected during household interviews include: demographic characteristics, health conditions, health status, use of medical services, sources of medical payments, and body weight and height. Each year the household survey includes approximately 12,000 households or 34,000 individuals. Sample size for those aged 51-64 is about 4,500. MEPS has comparable measures of social-economic (SES) variables as those in HRS, including age, race/ethnicity, educational level, census region, and marital status.

3.5 Medicare Current Beneficiary Survey (MCBS)

The MCBS is a nationally representative sample of aged, disabled and institutionalized Medicare beneficiaries. The MCBS attempts to interview each respondent twelve times over three years, regardless of whether he or she resides in the community, a facility, or

transitions between community and facility settings. The disabled (under 65 years of age) and oldest-old (85 years of age or older) are over-sampled. The first round of interviewing was conducted in 1991. Originally, the survey was a longitudinal sample with periodic supplements and indefinite periods of participation. In 1994, the MCBS switched to a rotating panel design with limited periods of participation. Each fall a new panel is introduced, with a target sample size of 12,000 respondents and each summer a panel is retired. Institutionalized respondents are interviewed by proxy. The MCBS contains comprehensive self-reported information on the health status, health care use and expenditures, health insurance coverage, and socioeconomic and demographic characteristics of the entire spectrum of Medicare beneficiaries. Medicare claims data for beneficiaries enrolled in fee-for-service plans are also used to provide more accurate information on health care use and expenditures.

4 Data Sources for Trends and Baseline Scenario

Two types of trends need to be projected in the model. First, we need to project trends in the incoming cohorts (the future new age 51/52 individuals). This includes trends in health and economic outcomes. Second, we need to project excess aggregate growth in real income and excess growth in health spending.

4.1 Data for Trends in Entering Cohorts

We used a multitude of data sources to compute U.S. trends. First, we used NHIS for chronic conditions and applied the methodology discussed in (D. Goldman et al., 2004). The method consists of projecting the experience of younger cohorts into the future until they reach age 51. The projection method is tailored to the synthetic cohorts observed in NHIS. For example, we observe a representative sample of age 35 individuals born in 1945 in 1980. We follow their disease patterns in 1980 to 1981 surveys by then selecting those aged 36 in 1981, accounting for mortality, etc.

We then collected information on other trends, i.e. for obesity and smoking, from other studies (Honeycutt et al., 2003; Levy, 2006; Mainous et al., 2007; Poterba, Venti, & Wise, 2007a, 2007b; Ruhm, 2007). Table 3 presents the sources and Table 4 presents the trends we use in the baseline scenario. Table 5 presents the prevalence of obesity, hypertension, diabetes, and current smokers in 1978 and 2004, and the annual rates of change from 1978 to 2004. We refer the readers to the analysis in Goldman et al. (2004) for information on how the trends were constructed.

4.2 Data for Other Projections

We make two assumptions relating to real growth in wages and medical costs. Firstly, as is done in the social security trustees report intermediate scenario, we assume a long term real increase in wages (earnings) of 1.1% per year. As is done by The Centers for Medicare and Medicaid Services, we assume excess real growth in medical costs (that is additional cost growth to GDP growth), as 1.5% in 2004, reducing linearly to 1% in 2033, .4% in 2053, and -.2% in 2083. We also include the Affordable Care Act cost growth targets as an optional cap on medical cost growth. Baseline medical spending figures presented assume those targets are met. GDP growth in the near term (through 2019) is based on CBO projections, with the OASDI Trustees assumption of 2% yearly afterwards.

4.3 Demographic Adjustments

We make two adjustments to the weighting in the Health and Retirement Study to match population counts from the Census. First, we post-stratify the HRS sample by 5 year age groups, gender and race and rebalance weights using the 2004 Current Population Survey (CPS). The CPS is itself matched to the decennial Census. Since we deleted some cases from the data and only considered the set of respondents with matched Social Security records, this takes account of selectivity based on these characteristics. We do this for both new cohort and host data set. The second adjustment we make is to scale up weights for future new cohorts using population projections from the Census Bureau. Again, we do this by race and gender. We use the intermediate net migration scenario produced by SSA in our simulation.

5 Estimation

In this section we describe the approach used to estimate the transition model, the core of the FEM, and the initial cohort model which is used to rejuvenate the model.

5.1 Transition Model

We consider a large set of outcomes for which we model transitions. Table 6 gives the set of outcomes considered for the transition model along with descriptive statistics and the population at risk when estimating the relationships.

Since we have a stock sample from the age 51+ population, each respondent goes through an individual-specific series of intervals. Hence, we have an unbalanced panel over the age range starting from 51 years old. Denote by j_{i0} the first age at which respondent *i* is observed and j_{iT_i} the last age when he is observed. Hence we observe outcomes at ages $j_i = j_{i0}, ..., j_{iT_i}$.

We first start with discrete outcomes which are absorbing states (e.g. disease diagnostic, mortality, benefit claiming). Record as $h_{i,j_i,m}$=1 if the individual outcome *m* has occurred as of age j_i. We assume the individual-specific component of the hazard

can be decomposed in a time invariant and variant part. The time invariant part is composed of the effect of observed characteristics x_i and permanent unobserved characteristics specific to outcome *m*, $h_{i,m}$. The time-varying part is the effect of previously diagnosed outcomes $h_{i,j_i-1,-m}$, (outcomes other than the outcome *m*) on the hazard for *m*.[10] We assume an index of the form $z_{m,j_i} = x_i b_m + h_{i,j_i-1,-m} g_m + h_{i,m}$. Hence, the latent component of the hazard is modeled as

$$\begin{aligned} &h^*_{i,j_i,m} = x_i b_m + h_{i,j_i-1,-m} g_m + h_{i,m} + a_{m,j_i} + e_{i,j_i,m}, \\ &m = 1,\ldots,M_0,\ j_i = j_{i0},\ldots,j_{iT_i},\ i = 1,\ldots,N \end{aligned} \quad . \qquad (1)$$

We approximate a_{m,j_i} with an age spline. After several specification checks, a node at age 75 appears to provide the best fit. This simplification is made for computational reasons since the joint estimation with unrestricted age fixed effects for each condition would imply a large number of parameters.

The outcome, conditional on being at risk, is defined as

$$\begin{aligned} &h_{i,j_i,m} = \max(I(h^*_{i,j_i,m} > 0), h_{i,j_i-1,m}) \\ &m = 1,\ldots,M_0,\ j_i = j_{i0},\ldots,j_{iT_i},\ i = 1,\ldots,N \end{aligned} \quad . \qquad (2)$$

As mentioned in the text we consider 8 outcomes which are absorbing states. The occurrence of mortality censors observation of other outcomes in a current year. Mortality is recorded from exit interviews.

A number of restrictions are placed on the way feedback is allowed in the model. Table 7 documents restrictions placed on the transition model. We also include a set of other controls. A list of such controls is given in Table 8 along with descriptive statistics. Since the "Did Statins Reduce the Health and Health Care Costs of Obesity?" article uses cohort simulations, this table presents descriptive statistics for the 2010 cohort used as baseline. We test the statistical significance of the restrictions on the health transition models (both the economic effect on health and the health effect on health) in Table 9. A number of them are statistically significant, and so we perform a robustness check on simulations not including these restrictions in section 7.2.

We have three other three other types of outcomes.

First, we have binary outcomes which are not an absorbing state. We specify latent indices as in (1) for these outcomes as well but where the lag dependent outcome also appears as a right-hand side variable. This allows for state-dependence.

Second, we have ordered outcomes. These outcomes are also modeled as in (1) recognizing the observation rule is a function of unknown thresholds V_m. Similarly to binary outcomes, we allow for state-dependence by including the lagged outcome on the right-hand side.

---

[10] With some abuse of notation, $j_i - 1$ denotes the previous age at which the respondent was observed.

The third type of outcomes we consider are censored outcomes, earnings and financial wealth. Earnings are only observed when individuals work. For wealth, there are a non-negligible number of observations with zero and negative wealth. For these, we consider two part models where the latent variable is specified as in (1) but model probabilities only when censoring does not occur. In total, we have M outcomes.

The term $\varepsilon_{i,j_i,m}$ is a time-varying shock specific to age j_i. We assume that this last shock is normally distributed and uncorrelated across diseases. Unobserved difference η_{im} are persistent over time and are allowed to be correlated across diseases $m=1,...,M$. We assume that these have a normal distribution with covariance matrix Ω_η.

The parameters $\theta_1 = (\{\beta_m, \gamma_m, V_m\}_{m=1}^{M}, vech(\Omega_\eta))$, can be estimated by maximum simulated likelihood. Given the normality distribution assumption on the time-varying unobservable, the joint probability of all time-intervals until failure, right-censoring or death conditional on the individual frailty is the product of normal univariate probabilities. Since these sequences, conditional on unobserved heterogeneity, are also independent across diseases, the joint probability over all disease-specific sequences is simply the product of those probabilities.

For a given respondent with frailty η_i observed from initial age j_{i0} to a last age j_{T_i}, the probability of the observed health history is (omitting the conditioning on covariates for notational simplicity)

$$l_i^{-0}(\theta;\eta_i,h_{i,j_{i0}}) = \left[\prod_{m=1}^{M-1}\prod_{j=j_{i1}}^{j_{T_i}} P_{ij,m}(\theta;\eta_i)^{(1-h_{ij-1,m})(1-h_{ij,M})}\right]\times\left[\prod_{j=j_{i1}}^{j_{T_i}} P_{ij,M}(\theta;\eta_i)\right] \quad (3)$$

We make explicit the conditioning on $h_{i,j_{i0}} = (h_{i,j_{i0},0},...,h_{i,j_{i0},M})'$, we have limited information on outcomes prior to this age.

To obtain the likelihood of the parameters given the observables, it remains to integrate out unobserved heterogeneity. The complication is that $h_{i,j_{i0},-m}$, the initial outcomes in each hazard is not likely to be independent of the common unobserved heterogeneity term which needs to be integrated out. A solution is to model the conditional probability distribution $p(\eta_i \mid h_{i,j_{i0}})$ (Wooldridge, 2000). Implementing this solution amounts to including initial outcomes at baseline each hazard. This is equivalent to writing

$$\eta_i = \Gamma h_{i0} + \alpha_i$$

$$\alpha_i \sim N(0,\Omega_\alpha)$$

Therefore, this allows for permanent differences in outcomes due to differences in baseline outcomes. The likelihood contribution for one respondent's sequence is therefore given by

$$l_i(\theta;h_{i,j_{i0}}) = \int l_i(\theta;\alpha_i,h_{i,j_{i0}})dF(\alpha_i) \quad (4)$$

To estimate the model, we make use of maximum simulated likelihood. We replace (4) with a simulated counterpart based on *R* draws from the distribution of a. We them optimize over this simulated likelihood using the BFGS algorithm. We could not obtain convergence of the joint estimator. So we assumed the distribution of a_i to be degenerate. This yielded the simpler estimation problem where each equation can be estimated separately.

One problem fitting the wealth and earnings distribution is that they have a long left-tail and wealth has some negative values. We use a generalization of the inverse hyperbolic sine transform (IHT), presented in (MacKinnon & Magee, 1990). First denote the variable of interest y. The hyperbolic sine transform is

$$y = \sinh(x) = \frac{\exp(y) - \exp(-y)}{2} \tag{5}$$

The inverse of the hyperbolic sin transform is

$$x = \sinh^{-1}(y) = h(y) = \log(y + (1 + y^2)^{0.5}) \tag{6}$$

Consider the inverse transformation. We can generalize such transformation, first allowing for a shape parameter q,

$$r(y) = h(qy)/q \tag{7}$$

Such that we can specify the regression model as

$$r(y) = xb + e, e \sim N(0, s^2). \tag{8}$$

A further generalization is to introduce a location parameter w such that the new transformation becomes

$$g(y) = \frac{h(q(y + w)) - h(qw)}{qh'(qw)} \tag{9}$$

where $h'(a) = (1 + a^2)^{-1/2}$.

We specify (8) in terms of the transformation g. The shape parameters can be estimated from the concentrated likelihood for q, w. We can then retrieve b, s by standard OLS.

Upon estimation, we can simulate

$$\tilde{g} = x\hat{b} + s\tilde{h} \tag{10}$$

where h is a standard normal draw. Given this draw, we can retransform using (9) and (5)

$$h(q(y + w)) = qh'(qw)\tilde{g} + h(qw)$$

$$\tilde{y} = \frac{\sinh[qh'(qw)\tilde{g} + h(qw)] - qw}{q}$$

Tables 10-14 give parameter estimates for the transition models.

5.2 Goodness-of-Fit

To judge the goodness-of-fit of the model, we estimated parameters on the 1998-2008 estimation sample and simulated outcomes of 1998 HRS respondents up to 2008. We then compared simulated and actual outcomes in 1998, 2004 and 2008. Table 15 presents the results. Some differences exist but in general the fit is satisfactory.

5.3 Quality Adjusted Life Years

As an alternative measure of life expectancy, we compute a quality adjusted life year based on the EQ-5D instrument, a health-related quality of life measure. The scoring system for EQ-5D was first developed by Dolan (1997) using a UK sample. Later a scoring system based on a US sample was generated (Shaw JW et al, 2005). Since the HRS does not ask the appropriate questions for compute EQ-5D, but the MEPS does, we use a crosswalk from the MEPS to the HRS for persons not living in a nursing home. The final OLS regression used to compute QALY in the FEM is shown in Table 26. If a person is living in a nursing home, then an additional 0.10 is subtracted from the computed QALY.

6 Model for New Cohorts

We first discuss the empirical strategy, then present the model and estimation results. The model for new cohorts integrates information coming from trends among younger cohorts with the joint distribution of outcomes in the current population of age 51 respondents in the HRS.

6.1 Information Available and Empirical Strategy

For the transition model, we need to first to obtain outcomes listed in Table 16. Ideally, we need information on

$$f_t(y_{i1},...,y_{iM}) = f_t(y_i) \tag{11}$$

where t denotes calendar time, and $y_i = (y_{i1},...,y_{iM})$ is a vector of outcomes of interest whose probability distribution at time t is $f_t()$. Information on how the joint distribution evolves over time is not available. Trends in conditional distributions are rarely reported either.

Generally, we have from published or unpublished sources good information on trends for some moments of each outcome (say a mean or a fraction). That is, we have information on

$$g_{t,m}(y_{i,m}) \qquad (12)$$

where $g_{t,m}()$ denotes the marginal probability distribution of outcome *m* at time *t.* For example we know from the NHIS repeated cross-sections that the fraction obese is increasing by roughly 2% a year among 51 year olds. In statistical jargon this means we have information on how the mean of the marginal distribution of y_{im}, an indicator variable that denotes whether someone is obese, is evolving over time.

We also have information on the joint distribution at one point in time, say year t_0. For example, we can estimate the joint distribution on age 51 respondents in the 1992 wave of the HRS, $f_{t_0}(y_i)$.

We make the assumption that only some part of $f_t(y_i)$ evolves over time. In particular, we will model the marginal distribution of each outcome allowing for correlation across these marginals. The correlations will be assumed fixed while the mean of the marginals will be allowed to change over time.

6.2 Model and Estimation

Assume the latent model for $y_i^* = (y_{i1}^*, ..., y_{iM}^*)'$,

$$y_i^* = \mu + \varepsilon_i \qquad (13)$$

where ε_i is normally distributed with mean zero and covariance matrix Ω. It will be useful to write the model as

$$y_i^* = \mu + L_\Omega \eta_i \qquad (14)$$

where L_Ω is a lower triangular matrix such that $L_\Omega L_\Omega' = \Omega$ and $\eta_i = (\eta_{i1}, ..., \eta_{iM})'$ are standard normal. We observe $y_i = \Gamma(y_i^*)$ which is a non-invertible mapping for a subset of the M outcomes. For example, we have binary, ordered and censored outcomes for which integration is necessary.

Because the mapping is non-invertible, integration needs to be performed to calculate the likelihood contributions $L_i(\theta \mid y_i)$. Integration needs to be done over a large number of dimensions. We will use maximum simulated likelihood to estimate the parameters of the model . The estimator is given by

$$\theta_{MSL} = \arg\max_{\theta=(\mu,\Omega)} \tfrac{1}{N} \sum_{i=1}^{N} \log \tfrac{1}{R} \sum_{r=1}^{R} \tilde{\Pr}(y_i \mid \theta)_r \qquad (15)$$

where $\frac{1}{R}\sum_{r=1}^{R}\tilde{\Pr}(y_i \mid \theta)_r$ is a consistent estimate of $\tilde{\Pr}(y_i \mid \theta)$. This estimator is consistent if both N, R tend to infinity. In practice, one can vary R to assess the bias of the estimator for smaller R. It is asymptotically efficient for $R/\sqrt{N}$ tending to infinity.

The vector μ_i can depend on some variables which have a stable distribution over time z_i (say race, gender and education). This way, estimation preserves the correlation with these outcomes without having to estimate their correlation with other outcomes. Hence, we can write

$$\mu_i = z_i \beta \tag{16}$$

and the whole analysis is done conditional on z_i.

For binary and ordered outcomes, we fix $\Omega_{m,m}$=1 which fixes the scale. Also we fix the location of the ordered models by fixing thresholds as $\tau_0 = -\infty, \tau_1 = 0, \tau_K = +\infty$ where K denotes the number of categories for a particular outcome. Because some of the binary outcomes are rare, we fix correlations to zero between two outcomes if both fraction positive are below 10%. Furthermore, we fix to zero the correlation between selected outcomes (say earnings) and their selection indicator. Hence, we consider two-part models for these outcomes.

For exposition, we order the observed outcomes as binary, ordered, continuous and finally censored. The GHK simulator can be used to simulate $\Pr(y_i \mid \theta)$.

We start with the first outcome y_{i1}^*, a discrete outcome.

1. A draw of η_{i1} consistent with observed choice y_{i1} is

$$\tilde{\eta}_{i1} = \Phi^{-1}[\tilde{u}_{i1}\Phi(\tfrac{\bar{c}_{i1}-\mu_{i1}}{L_{\Omega,11}}) + (1-\tilde{u}_{i1})\Phi(\tfrac{\underline{c}_{i1}-\mu_{i1}}{L_{\Omega,11}})] \tag{17}$$

   where $\bar{c}_{i1} = \begin{cases} +\infty & \text{if } y_{i1}=1 \\ 0 & \text{if } y_{i1}=0 \end{cases}, \underline{c}_{i1} = \begin{cases} 0 & \text{if } y_{i1}=1 \\ -\infty & \text{if } y_{i1}=0 \end{cases}$ and $\tilde{u}_{i1}$ is a uniform draw. The bounds are slightly different for ordered outcomes where thresholds are also estimated. In particular we have
   $\bar{c}_{i1} = \tau_k, \underline{c}_{i1} = \tau_{k-1}$ if $y_{i1} = k$
   where τ_k are parameters to be estimated.
2. The probability of that first outcome is $\tilde{\Pr}(y_{i1} \mid \theta) = \Phi(\frac{\bar{c}_{i1}-\mu_{i1}}{L_{\Omega,11}}) - \Phi(\frac{\underline{c}_{i1}-\mu_{i1}}{L_{\Omega,11}})$
3. Now a draw of η_{i2} consistent with y_{i2} and the draw $\tilde{\eta}_{i1}$ is given by

$$\tilde{\eta}_{i2} = \Phi^{-1}[\tilde{u}_{i2}\Phi(\tfrac{\bar{c}_{i2}-\mu_{i2}-L_{\Omega,21}\tilde{\eta}_{i1}}{L_{\Omega,22}}) + (1-\tilde{u}_{i2})\Phi(\tfrac{\underline{c}_{i2}-\mu_{i2}-L_{\Omega,21}\tilde{\eta}_{i1}}{L_{\Omega,22}})]$$

4. Then the probability is given by

$$\tilde{\Pr}(y_{i1}, y_{i2} \mid \theta) = \tilde{\Pr}(y_{i1} \mid \theta)[\Phi(\tfrac{\bar{c}_{i2}-\mu_{i2}-L_{\Omega,21}\tilde{\eta}_{i1}}{L_{\Omega,22}}) - \Phi(\tfrac{\underline{c}_{i2}-\mu_{i2}--L_{\Omega,21}\tilde{\eta}_{i2}}{L_{\Omega,22}})] \tag{18}$$

5. Cycle trough 3 and 4 until end of discrete outcomes. Denote by $m_0 - 1$ the number of discrete outcomes.
6. An error consistent with the first continuous outcome is

$$\tilde{\eta}_{im_0} = \frac{y_{i,m_0} - \mu_{i,m_0} - \sum_{s=1}^{m_0-1} \Lambda_{\Omega,m_0,s} \tilde{\eta}_{is}}{\Lambda_{\Omega,m_0,m_0}}$$

7. The probability is $\tilde{\Pr}(y_{i,m_0} \mid \theta) = \frac{1}{\Lambda_{\Omega,m_0,m_0}} \phi\left(\frac{y_{i,m_0} - \mu_{i,m_0} - \sum_{s=1}^{m_0-1} \Lambda_{\Omega,m_0,s} \tilde{\eta}_{is}}{\Lambda_{\Omega,m_0,m_0}}\right)$
8. Hence $\tilde{\Pr}(y_{i1},...,y_{i,m_0} \mid \theta) = \tilde{\Pr}(y_{i1},...,y_{i,m_0-1} \mid \theta)\tilde{\Pr}(y_{i,m_0} \mid \theta)$
9. Cycle trough 6 to 8 until reach $m_1 - 1$, the last continuous outcome.
10. Denote by m_1 the first censored outcome. Denote by y_{ij} the binary outcome that records whether y_{im_1} can be observed. A draw consistent with y_{i,m_1} is given by

$$\tilde{\eta}_{im_1} = \Phi^{-1}[\tilde{u}_{im_1}] \text{ if } y_{ij} = 0$$

and

$$\tilde{\eta}_{im_1} = \frac{y_{i,m1} - \mu_{i,m1} - \sum_{s=1}^{m_1-1} \Lambda_{\Omega,m_1,s} \tilde{\eta}_{is}}{\Lambda_{\Omega,m_1,m_1}} \text{ if } y_{ij} = 1$$

If y_{im1} is continuous and given by a draw similar to (7) if a binary outcome.
11. The probability is then

$$\tilde{\Pr}(y_{i,m_1} \mid \theta) = \left[\frac{1}{\Lambda_{\Omega,m_1,m1}} \phi\left(\frac{y_{i,m1} - \mu_{i,m_1} - \sum_{s=1}^{m_1-1} \Lambda_{\Omega,m1,s} \tilde{\eta}_{is}}{\Lambda_{\Omega,m_1,m_1}}\right)\right]^{I(y_{i,j}=1)}$$

for continuous and cumulative normal similar to (8) for discrete.

12. Cycle 10-11 until reach M.
13. Repeat 1-9 R times and calculate $\frac{1}{R}\sum_{r=1}^{R} \tilde{\Pr}(y_i \mid \theta)_r$.
14. Repeat for each $i = 1,...,N$

We use draws from Halton sequences to generate uniform random draws (Train, 2003). Note that draws $\left\{\left\{\left\{u_{im,r}\right\}_{m=1,...,M}\right\}_{r=1,...,R}\right\}_{i=1,...,N}$ are kept fixed trough estimation. For the first past, we used 10 draws along each dimension.

Because some parameters are naturally bounded, we re-parameterize the problem to guarantee an interior solution. In particular, we parameterize

$$\Omega_{m,m} = \exp(\delta_m),\ m = m_0 - 1,...,M$$
$$\Omega_{m,n} = \tanh(\xi_{m,n})\sqrt{\Omega_{m,m}\Omega_{n,n}},\ m,n = 1,...,M$$
$$\tau_{m,k} = \exp(\gamma_{m,k}) + \tau_{k-1},\ k = 2,...,K_m - 1, m \text{ ordered}$$

and estimate the $(\delta_{m,m}, \xi_{m,n}, \gamma_k)$ instead of the original parameters. Table 17 gives parameter estimates for the indices while Table 18 gives parameter estimates of the covariance matrix in the outcomes.

The latent model is written as

$$y_i^* = m + L_W h_i$$

Each marginal has a mean change equal to $E(y \mid m) = (1+t)g(m)$, where t is the percent change in the outcome and $g()$ is a non-linear but monotone mapping. Since it is invertible, we can find the vector m^* where $m^* = g^{-1}(E(y \mid m)/(1+t))$. We use these new intercepts to simulate new outcomes.

7 Government Revenue and Expenditures

This gives a limited overview of how revenues and expenditures of the government are computed. These functions are based on 2004 rules but we include predicted changes in program rules such changes based on year of birth (e.g. Normal retirement age).

We cover the following revenues and expenditures:

**Revenues**	**Expenditures**
Federal Income Tax	Social Security Retirement benefits
State and City Income Taxes	Social Security Disability benefits
Social Security Payroll Tax	Supplementary Security Income (SSI)
Medicare Payroll Tax	Medical Care Costs

7.1 Social Security Benefits

Workers with 40 quarters of coverage and of age 62 are eligible to receive their retirement benefit. The benefit is calculated based on the Average Indexed Monthly Earnings (AIME) and the age at which benefits are first received. If an individual claims at his normal retirement age (NRA) (65 for those born prior to 1943, 66 for those between 1943 and 1957, and 67 thereafter), he receives his Primary Insurance Amount (PIA) as a monthly benefit. The PIA is a piece-wise linear function of the AIME. If a worker claims prior to his NRA, his benefit is lower than his PIA. If he retires after the NRA, his benefit is higher. While receiving benefits, earnings are taxed above a certain earning disregard level prior to the NRA. An individual is eligible to half of his spouse's PIA, properly adjusted for the claiming age, if that is higher than his/her own retirement benefit. A surviving spouse is eligible to the deceased spouse's PIA. Since we assume prices are constant in our simulations, we do not adjust benefits for the COLA (Cost of Living Adjustment) which usually follows inflation. We however adjust the PIA bend points for increases in real wages.

7.2 Disability Insurance Benefits

Workers with enough quarters of coverage and under the normal retirement age are eligible for their PIA (no reduction factor) if they are judged disabled (which we take as the predicted outcome of DI receipt) and earnings are under a cap called the Substantial Gainful Activity (SGA) limit. This limit was $9720 in 2004. We ignore the 9 month trial period over a 5 year window in which the SGA is ignored.

7.3 Supplemental Security Income Benefits

Self-reported receipt of supplemental security income (SSI) in the HRS provides estimates of the proportion of people receiving SSI under what administrative data would suggest. To correct for this bias, we link the HRS with administrative data from the social security administration identifying those receiving SSI. In the linked administrative data, 3.96% of the population receives supplementary security income, while only 2.79% of the sample reports social security income. We therefore estimate a probit of receiving SSI as a function of self-reporting social security income, as well as demographic, health, and wealth. Table 11 contains the estimates for this model.

The benefit amount is taken from the average monthly benefits found in the 2004 Social Security Annual Statistical Supplement. We assign monthly benefit of $450 for person aged 51 to 64, and $350 for persons aged 65 and older.

7.4 Medical Costs Estimation

In the FEM, a cost module links a person's current state—demographics, economic status, current health, risk factors, and functional status—to 4 types of individual medical spending. The FEM models: total medical spending (medical spending from all payment sources), Medicare spending[11], Medicaid spending (medical spending paid by Medicaid), and out of pocket spending (medical spending by the respondent). These estimates are based on pooled weighted least squares regressions of each type of spending on risk factors, self-reported conditions, and functional status, with spending inflated to constant dollars using the medical component of the consumer price index. We use the 2002-2004 Medical Expenditure Panel Survey (n = 14,098) for these regressions for persons not Medicare eligible, and the 2002-2004 Medicare Current Beneficiary Survey (n = 33, 231) for spending for those that are eligible for Medicare. Those eligible for Medicare include people eligible due to age (65+) or due to disability status. A comparison across these different sources is provided in Table 2.

In the baseline scenario, this spending estimate can be interpreted as the resources consumed by the individual given the manner in which medicine is practiced in the United States at the beginning of the 21st century. Table 19 shows the model estimation results for total, Medicaid, and out of pocket spending, while Table 20 shows the model estimation results for the Medicare spending. These estimation results only use the MCBS dataset.

---

[11] We estimate annual medical spending paid by specific parts of Medicare (Parts A, B, and D) and sum to get the total Medicare expenditures.

Since Medicare spending has numerous components (Parts A and B are considered here), models are needed to predict enrollment. In 2004, 98.4% of all Medicare enrollees, and 99%+ of aged enrollees, were in Medicare Part A, and thus we assume that all persons eligible for Medicare take Part A. We use the 1999-2004 MCBS to model take up of Medicare Part B for both new enrollees into Medicare, as well as current enrollees without Part B. Estimates are based on weighted probit regression on various risk factors, demographic, and economic conditions. The HRS starting population for the FEM does not contain information on Medicare enrollment. Therefore another model of Part B enrollment for all persons eligible for Medicare is estimated via a probit, and used in the first year of simulation to assign initial Part B enrollment status. Estimation results are shown in Table 21. The MCBS data over represents the portion enrolled in Part B, having a 97% enrollment rate in 2004 instead of the 93.5% rate given by Medicare Trustee's Report. In addition to this baseline enrollment probit, we apply an elasticity to premiums of -0.10, based on the literature and simulation calibration for actual uptake through 2009 (Atherly, Dowd, and Feldman, 2004; Buchmueller, 2006). The premiums are computed using average Part B costs from the previous time step and the means-testing thresholds established by the ACA.

Since both the MEPS and MCBS are known to under-predict medical spending, we applied adjustment factors to the predicted three types of individual medical spending so that in year 2004, the predicted per-capita spending in FEM equal the corresponding spending in National Health Expenditure Accounts (NHEA), for age group 55-64 and 65 and over, respectively. Table 22 shows how these adjustment factors were determined by using the ratio of expenditures in the NHEA to expenditures predicted in the FEM.

The Medicare Current Beneficiaries Survey (MCBS) 2006 contains data on Medicare Part D. The data gives the capitated Part D payment and enrollment. When compared to the summary data presented in the CMS 2007 Trustee Report, the per capita cost is comparable between the MCBS and the CMS. However, the enrollment is underestimated in the MCBS, 53% compared to 64.6% according to CMS.

Since only one year of Part D enrollment is available in the MCBS, only a cross sectional model of Part D enrollment is estimated, rather than a transition model as with Part B enrollment. A probit model is estimated to link demographics, economic status, current health, and functional status to Part D enrollment - see Tables 23 and 24 for estimates. To account for both the initial under reporting of Part D enrollment in the MCBS, as well as the CMS prediction that Part D enrollment will rise to 75% by 2012, the constant in the probit model is increased by 0.22 in 2006, to 0.56 in 2012 and beyond. The per capita Part D cost in the MCBS matches well with the cost reported from CMS. An OLS regression using demographic, current health, and functional status is estimated for Part D costs.

The Part D enrollment and cost models are implemented in the Medical Cost module. The Part D enrollment model is executed conditional on the person being eligible for Medicare, and the cost model is executed conditional on the enrollment model leading a true result, after the Monte Carlo decision. Otherwise the person has zero Part D cost. The estimated Part D costs are added with Part A and B costs to obtain total Medicare cost, and any medical cost growth assumptions are then applied.

7.5 Taxes

We consider Federal, State and City taxes paid at the household level. We also calculate Social Security taxes and Medicare taxes. HRS respondents are linked to their spouse in the HRS simulation. We take program rules from the OECD's Taxing Wages Publication for 2004. Households have basic and personal deductions based on marital status and age (>65). Couples are assumed to file jointly. Social Security benefits are partially taxed. The amount taxable increases with other income from 50% to 85%. Low income elderly have access to a special tax credit and the earned income tax credit is applied for individuals younger than 65. We calculate state and city taxes for someone living in Detroit, Michigan. The OECD chose this location because it is generally representative of average state and city taxes paid in the U.S. Since Social Security administrative data cannot be used jointly with Geocoded information in the HRS, we apply these hypothetical taxes to all respondents.

At the state level, there is a basic deduction for each member of the household ($3100) and taxable income is taxed at a flat rate of 4%. At the city level, there is a small deduction of $750 per household member and the remainder is taxed at a rate of 2.55%. There is however a tax credit that decreases with income (20% on the first 100$ of taxes paid, 10% on the following 50$ and 5% on the remaining portion).

We calculate taxes paid by the employee for Old-Age Social Insurance (SS benefits and DI) and Medicare (Medicaid and Medicare). It does not include the equivalent portion paid by the employer. OASI taxes of 6.2% are levied on earnings up to $97,500 (2004 cap) while the Medicare tax (1.45%) is applied to all earnings.

8 Implementation of the FEM

The FEM is implemented in multiple parts. Estimation of the transition and cross sectional models is performed in Stata, the incoming cohort model is estimated in Ox, and the simulation is implemented in C++ to increase speed.

To match the two year structure of the Health and Retirement Study (HRS) data used to estimate the transition models, the FEM simulation proceeds in two year increments. The end of each two year step is designed to occur on July 1st to allow for easier matching to population forecasts from Social Security. A simulation of the FEM proceeds by first loading a population representative of the age 51+ US population in 2004, generated from HRS. In two year increments, the FEM applies the transition models for mortality, health, working, wealth, earnings, and benefit claiming with Monte Carlo decisions to calculate the new states of the population. The population is also adjusted by immigration forecasts from the US Census Department, stratified by race and age. If incoming cohorts are being used, the new 51/52 year olds are added to the population. The number of new 51/52 year olds added is consistent with estimates from the Census, stratified by race. Once the new states have been determined and new 51/52 year olds added, the cross sectional models for medical costs, and calculations for government expenditures and revenues are performed. Summary variables are then computed. Computation of medical costs includes the persons that died to account for end of life costs. Other computations, such as social security benefits and government tax

revenues, are restricted to persons alive at the end of each two year interval. To eliminate uncertainty due to the Monte Carlo decision rules, the simulation is performed multiple times (typically 100), and the mean of each summary variable is calculated across repetitions.

FEM simulation takes as inputs assumptions regarding growth in the national wage index, normal retirement age, real medical cost growth, interest rates, cost of living adjustments, the consumer price index, significant gainful activity, and deferred retirement credit. The default assumptions are taken from the 2010 Social Security Intermediate scenario, adjusted for no price increases after 2010. Therefore simulation results are in real 2009 dollars.

Different simulation scenarios are implemented by changing any of the following components: incoming cohort model, transition models, interventions that adjust the probabilities of specific transition, and changes to assumptions on future economic conditions.

References Cited in Section 1

Baigent, C., Blackwell, L., Collins, R., Emberson, J., Godwin, J., Peto, R., . . . Meade, T. (2009). Aspirin in the primary and secondary prevention of vascular disease: collaborative meta-analysis of individual participant data from randomised trials. *Lancet, 373*(9678), 1849-1860. doi:10.1016/S0140-6736(09)60503-1

Berger, J. S., Brown, D. L., & Becker, R. C. (2008). Low-dose aspirin in patients with stable cardiovascular disease: a meta-analysis. *Am J Med, 121*(1), 43-49. doi:10.1016/j.amjmed.2007.10.002

Berger, J. S., Lala, A., Krantz, M. J., Baker, G. S., & Hiatt, W. R. (2011). Aspirin for the prevention of cardiovascular events in patients without clinical cardiovascular disease: A meta-analysis of randomized trials. *American heart journal, 162*(1), 115-U159. doi:10.1016/j.ahj.2011.04.006

Berger, J. S., Roncaglioni, M. C., Avanzini, F., Pangrazzi, I., Tognoni, G., & Brown, D. L. (2006). Aspirin for the primary prevention of cardiovascular events in women and men: a sex-specific meta-analysis of randomized controlled trials. *JAMA, 295*(3), 306-313. doi:10.1001/jama.295.3.306

Campbell, H. E., Stokes, E. A., Bargo, D., Logan, R. F., Mora, A., Hodge, R., . . . investigators, T. (2015). Costs and quality of life associated with acute upper gastrointestinal bleeding in the UK: cohort analysis of patients in a cluster randomised trial. *BMJ open, 5*(4), e007230. doi:10.1136/bmjopen-2014-007230

Cuzick, J., Thorat, M., Bosetti, C., Brown, P., Burn, J., Cook, N., . . . La Vecchia, C. (2014). Estimates of benefits and harms of prophylactic use of aspirin in the general population. *Annals of Oncology*, mdu225.

Gaudette, É., Goldman, D. P., Messali, A., & Sood, N. (2015). Do Statins Reduce the Health and Health Care Costs of Obesity? *PharmacoEconomics, 33*(7), 723-734.

Gaudette, É., Tysinger, B., Cassil, A., & Goldman, D. P. (2015). *Health and Health Care of Medicare Beneficiaries in 2030.* Paper presented at the Forum for Health Economics and Policy.

Ghate, S. R., Biskupiak, J., Ye, X., Kwong, W. J., & Brixner, D. I. (2011). All-cause and bleeding-related health care costs in warfarin-treated patients with atrial fibrillation. *J Manag Care Pharm, 17*(9), 672-684. doi:10.18553/jmcp.2011.17.9.672

Goldman, D., Hurd, M., Shekelle, P., Newberry, S., Panis, C., Shang, B., . . . Lakdawalla, D. (2004). *Health status and medical treatment of the future elderly: Final Report* (TR-169). Retrieved from Santa Monica, CA: http://www.rand.org/publications/TR/TR169/

Goldman, D. P., Cutler, D., Rowe, J. W., Michaud, P. C., Sullivan, J., Peneva, D., & Olshansky, S. J. (2013). Substantial health and economic returns from delayed aging may warrant a new focus for medical research. *Health Affairs, 32*(10), 1698-1705. doi:10.1377/hlthaff.2013.0052

Goldman, D. P., Gaudette, É., & Cheng, W.-H. (2016). Competing Risks: Investing in Sickness Rather Than Health. *American Journal of Preventive Medicine, 50*(5, Supplement 1), S45-S50. doi:http://dx.doi.org/10.1016/j.amepre.2015.12.005

Goldman, D. P., Shekelle, P. G., Bhattacharya, J., Hurd, M., & Joyce, G. F. (2004). *Health status and medical treatment of the future elderly*. Retrieved from

Honeycutt, A. A., Boyle, J. P., Broglio, K. R., Thompson, T. J., Hoerger, T. J., Geiss, L. S., & Narayan, K. M. (2003). A dynamic Markov model for forecasting diabetes prevalence in the United States through 2050. *Health care management science, 6*(3), 155-164.

Ikeda, Y., Shimada, K., Teramoto, T., Uchiyama, S., Yamazaki, T., Oikawa, S., . . . Ishizuka, N. (2014). Low-dose aspirin for primary prevention of cardiovascular events in Japanese patients 60 years or older with atherosclerotic risk factors: a randomized clinical trial. *JAMA, 312*(23), 2510-2520. doi:10.1001/jama.2014.15690

Levy, D. (2006). *Trends in Smoking Rates Under Different Tobacco Control Policies:Results from the SimSmoke Tobacco Policy Simulation Model*.

MacKinnon, J. G., & Magee, L. (1990). Transforming the Dependent Variable in Regression Models. *International Economic Review, 31*(2), 315-339.

Mainous, A. G., 3rd, Baker, R., Koopman, R. J., Saxena, S., Diaz, V. A., Everett, C. J., & Majeed, A. (2007). Impact of the population at risk of diabetes on projections of diabetes burden in the United States: an epidemic on the way. *Diabetologia, 50*(5), 934-940.

McQuaid, K. R., & Laine, L. (2006). Systematic review and meta-analysis of adverse events of low-dose aspirin and clopidogrel in randomized controlled trials. *Am J Med, 119*(8), 624-638. doi:10.1016/j.amjmed.2005.10.039

Michaud, P.-C., Kapteyn, A., Smith, J. P., & van Soest, A. (2006). *The Effects of Attrition and Non-Response in the Health and Retirement Study*. Retrieved from Santa Monica, CA:

Michaud, P. C., Goldman, D. P., Lakdawalla, D. N., Zheng, Y., & Gailey, A. H. (2012). The value of medical and pharmaceutical interventions for reducing obesity. *Journal of Health Economics, 31*(4), 630-643. doi:10.1016/j.jhealeco.2012.04.006

Poterba, J., Venti, S., & Wise, D. A. (2007a). The Decline of Defined Benefit Retirement Plans and Asset Flows: National Bureau of Economic Research, Inc, NBER Working Papers: 12834.

Poterba, J., Venti, S., & Wise, D. A. (2007b). New Estimates of the Future Path of 401(k) Assets: National Bureau of Economic Research, Inc, NBER Working Papers: 13083.

Raju, N., Sobieraj-Teague, M., Hirsh, J., O'Donnell, M., & Eikelboom, J. (2011). Effect of aspirin on mortality in the primary prevention of cardiovascular disease. *Am J Med, 124*(7), 621-629. doi:10.1016/j.amjmed.2011.01.018

Rothwell, P. M., Fowkes, F. G. R., Belch, J. F., Ogawa, H., Warlow, C. P., & Meade, T. W. (2011). Effect of daily aspirin on long-term risk of death due to cancer: analysis of individual patient data from randomised trials. *The Lancet, 377*(9759), 31-41.

Rothwell, P. M., Wilson, M., Elwin, C. E., Norrving, B., Algra, A., Warlow, C. P., & Meade, T. W. (2010). Long-term effect of aspirin on colorectal cancer incidence and mortality: 20-year follow-up of five randomised trials. *Lancet, 376*(9754), 1741-1750. doi:10.1016/S0140-6736(10)61543-7

Ruhm, C. (2007). Current and Future Prevalence of Obesity and Severe Obesity in the United States. *Forum for Health Economics & Policy, 10*(2).

Seshasai, S. R., Wijesuriya, S., Sivakumaran, R., Nethercott, S., Erqou, S., Sattar, N., & Ray, K. K. (2012). Effect of aspirin on vascular and nonvascular outcomes: meta-analysis of randomized controlled trials. *Arch Intern Med, 172*(3), 209-216. doi:10.1001/archinternmed.2011.628

Smith, S. C., Jr., Benjamin, E. J., Bonow, R. O., Braun, L. T., Creager, M. A., Franklin, B. A., . . . Taubert, K. A. (2011). AHA/ACCF secondary prevention and risk reduction therapy for patients with coronary and other atherosclerotic vascular disease: 2011 update: a guideline from the American Heart Association and American College of Cardiology Foundation endorsed by the World Heart Federation and the Preventive Cardiovascular Nurses Association. *J Am Coll Cardiol, 58*(23), 2432-2446. doi:10.1016/j.jacc.2011.10.824

Thorat, M. A., & Cuzick, J. (2015). Prophylactic use of aspirin: systematic review of harms and approaches to mitigation in the general population. *European journal of epidemiology, 30*(1), 5-18.

Train, K. (2003). *Discrete Choice Methods with Simulation*. Cambridge: Cambridge University Press.

US Preventive Services Task Force. (2009). Aspirin for the prevention of cardiovascular disease: US Preventive Services Task Force recommendation statement. *Annals of Internal Medicine, 150*(6), 396.

Wooldridge, J. M. (2000). A framework for estimating dynamic, unobserved effects panel data models with possible feedback to future explanatory variables. *Economics Letters, 68*(3), 245-250.

Zissimopoulos, J., Crimmins, E., & St.Clair, P. (2015). The Value of Delaying Alzheimer’s Disease Onset. *Forum for Health Economics and Policy, 18*(1), 25. doi:10.1515/fhep-2014-0013

References Cited in Sections 2-8

Atherly A, Dowd B, Feldman R. 2004. " The Effects of Benefits, Premiums, and Health Risk on Health Plan Choice in the Medicare Program." *Health Services Research*, 39(4): 847-864.

Baigent, C., Blackwell, L., Collins, R., Emberson, J., Godwin, J., Peto, R., ... & Patrono, C. (2009). Aspirin in the primary and secondary prevention of vascular disease: collaborative meta-analysis of individual participant data from randomised trials. Lancet, 373(9678), 1849-60.

Berger, J. S., Brown, D. L., & Becker, R. C. (2008). Low-dose aspirin in patients with stable cardiovascular disease: a meta-analysis. The American journal of medicine, 121(1), 43-49.

Berger, J. S., Roncaglioni, M. C., Avanzini, F., Pangrazzi, I., Tognoni, G., & Brown, D. L. (2006). Aspirin for the primary prevention of cardiovascular events in women and men: a sex-specific meta-analysis of randomized controlled trials. Jama, 295(3), 306-313.

Berger, J. S., Lala, A., Krantz, M. J., Baker, G. S., & Hiatt, W. R. (2011). Aspirin for the prevention of cardiovascular events in patients without clinical cardiovascular disease: a meta-analysis of randomized trials. American heart journal, 162(1), 115-124.

Buchmueller, T. 2006. "Price and the Health Plan Choices of Retirees." *Journal of Health Economics*, 25: 81-101.

Dolan P. Modeling valuations for EuroQol health states.Med care 1997 Nov;35(11):1095-10.

Gaudette, É., Goldman, D. P., Messali, A., & Sood, N. (2015). Do Statins Reduce the Health and Health Care Costs of Obesity?. PharmacoEconomics, 1-12.

Gaudette, É., Tysinger, B., Cassil, A., & Goldman, D. P. (2015). Health and Health Care of Medicare Beneficiaries in 2030. Forum for Health Economics and Policy (Vol. 18, No. 2, pp. 75-96).

Goldman DP, et al. (2004). Health status and medical treatment of the future elderly: Final Report. Santa Monica, CA: RAND Corporation.

Goldman, D. P., Cutler, D., Rowe, J. W., Michaud, P. C., Sullivan, J., Peneva, D., & Olshansky, S. J. (2013). Substantial health and economic returns from delayed aging may warrant a new focus for medical research. Health affairs, 32(10), 1698-1705.

Goldman, Dana P., Étienne Gaudette, and Wei-Han Cheng. "Competing Risks: Investing in Sickness Rather Than Health." American Journal of Preventive Medicine 50, no. 5 (2016): S45-S50.

Gutierrez J, Ramirez G, Rundek T, Sacco RL. Statin therapy in the prevention of recurrent cardiovascular events: a sex-based meta-analysis. Archives of internal medicine. 2012 Jun 25;172(12):909-19.

Health and Retirement Study, (RAND HRS). Produced and distributed by the University of Michigan with funding from the National Institute on Aging (grant number NIA U01AG009740). Ann Arbor, MI, (2012).

Honeycutt, A. A., J. P. Boyle, K. R. Broglio, T. J. Thompson, T. J. Hoerger, L. S. Geiss, and K. M. Narayan. 2003. A dynamic Markov model for forecasting diabetes

prevalence in the United States through 2050. Health care management science 6 (3):155-64.

Levy, D. 2006. Trends in Smoking Rates Under Different Tobacco Control Policies:Results from the SimSmoke Tobacco Policy Simulation Model.

MacKinnon, James G., and Lonnie Magee. 1990. Transforming the Dependent Variable in Regression Models. International Economic Review 31 (2):315-339.

Mainous, A. G., 3rd, R. Baker, R. J. Koopman, S. Saxena, V. A. Diaz, C. J. Everett, and A. Majeed. 2007. Impact of the population at risk of diabetes on projections of diabetes burden in the United States: an epidemic on the way. Diabetologia 50 (5):934-40.

Michaud, P. C., Goldman, D. P., Lakdawalla, D. N., Zheng, Y., & Gailey, A. H. (2012). The value of medical and pharmaceutical interventions for reducing obesity. Journal of health economics, 31(4), 630-643.

Michaud, P.-C., A. Kapteyn, J. P. Smith, and A. van Soest. 2006. The Effects of Attrition and Non-Response in the Health and Retirement Study. In RAND Working Paper 407. Santa Monica, CA.

Poterba, James, Steven Venti, and David A Wise. 2007. The Decline of Defined Benefit Retirement Plans and Asset Flows: National Bureau of Economic Research, Inc, NBER Working Papers: 12834.

———. 2007. New Estimates of the Future Path of 401(k) Assets: National Bureau of Economic Research, Inc, NBER Working Papers: 13083.

Raju, N., Sobieraj-Teague, M., Hirsh, J., O'Donnell, M., & Eikelboom, J. (2011). Effect of aspirin on mortality in the primary prevention of cardiovascular disease. The American journal of medicine, 124(7), 621-629.

Reville, Robert T., Seth A. Seabury, Frank Neuhauser, John F. Burton, Michael R. Greenberg, Institute for Civil Justice (U.S.), Rand Corporation., and California. Commission on Health and Safety and Workers' Compensation. 2005. An evaluation of California's permanent disability rating system. Santa Monica, CA: RAND Corp.

Rothwell, P. M., Wilson, M., Elwin, C. E., Norrving, B., Algra, A., Warlow, C. P., & Meade, T. W. (2010). Long-term effect of aspirin on colorectal cancer incidence and mortality: 20-year follow-up of five randomised trials. The Lancet, 376(9754), 1741-1750.

Rothwell, P. M., Fowkes, F. G. R., Belch, J. F., Ogawa, H., Warlow, C. P., & Meade, T. W. (2011). Effect of daily aspirin on long-term risk of death due to cancer: analysis of individual patient data from randomised trials. The Lancet, 377(9759), 31-41.

Ruhm, Christopher. 2007. Current and Future Prevalence of Obesity and Severe Obesity in the United States. Forum for Health Economics & Policy 10 (2).

Seshasai, S. R. K., Wijesuriya, S., Sivakumaran, R., Nethercott, S., Erqou, S., Sattar, N., & Ray, K. K. (2012). Effect of aspirin on vascular and nonvascular outcomes: meta-analysis of randomized controlled trials. Archives of internal medicine, 172(3), 209-216.

Shaw JW, Johnson JA, Coons SJ. US Valuation of the EQ-5D Health States: Development and Testing of the D1 Valuation Model, Med Care 2005, 43(3), 203-220.

Taylor F, Huffman MD, Macedo AF, Moore TH, Burke M, Davey Smith G, et al. Statins for the primary prevention of cardiovascular disease. The Cochrane database of systematic reviews. 2013;1:CD004816.

Train, K. 2003. Discrete Choice Methods with Simulation. Cambridge: Cambridge University Press.

Wooldridge, Jeffrey M. 2000. A framework for estimating dynamic, unobserved effects panel data models with possible feedback to future explanatory variables. Economics Letters 68 (3):245-250.

Technical Tables

List of Technical Tables

Table Number	Short Title
1	Comparison of Questions on Chronic Health Conditions in HRS and SHARE
2	Comparison of Prevalence and Questions on Chronic Health Conditions in HRS, NHIS, MEPS, and MCBS
3	Data Sources and Methods for Projecting Trends for Future Cohorts
4	Projected Trends in the U.S.
5	Prevalence of Obesity, Hypertension, Diabetes and Current Smokers Among Aged 47-56 in 1978 and in 2004
6	Outcomes in the Transition Model
7	Restrictions on Health Outcomes
8	Descriptive Statistics on Exogenous Controls (2010 cohort)
9	Statistical Test of Restrictions on Health Transition Model
10	Transition of Mortality and Chronic Conditions (coefficient/t statistic)
11	Transition of Economic Outcomes (coefficient/t statistic)
12	OLS regression for Log(BMI) (coefficient/t statistic)
13	Ordered Probits for Transition of Smoking, and Functional Status (coefficient/t statistic)
14	Estimating Earnings and Household Wealth Using Inverse Hyperbolic Sine Transformation Preserving Shape (coefficient/t statistic)
15	Comparison of Predicted and Observed Outcomes for HRS Cohort in 2004
16	Descriptive Statistics Initial Conditions for Estimation (1992) and Simulation (2004)
17	Parameter Estimates for Latent Model (Conditional Mean and Thresholds)
18	Parameter Estimates: Parameterized Covariance Matrix
19	Regression Estimates of Medical Spending (coefficient/t statistic)
20	Regression Estimates of Medicare Spending in Parts A and B (coefficient/t statistic)
21	Probit Regression Estimates on Medicare Part B Take up (coefficient/t statistic)
22	Compare per capita medical spending by payment sources and by age group in year 2004
23	Probit Regression Estimates on Medicare Part D Enrollment (coefficient/t statistic)
24	OLS Regression Estimates on Medicare Part D Spending (coefficient/t statistic)
25	Status Quo FEM Estimates
26	Quality Adjusted Life Year Model
27	Cross Validation Results

Table 1

	HRS	SHARE
Question	Has a doctor ever told you that you have …	Has a doctor ever told you that you had any of the conditions on this card? Please tell me the number or numbers of the conditions
Heart Disease	… a heart attack, coronary heart disease, angina, congestive heart failure, or other heart problems?	… A heart attack including myocardial infarction or coronary thrombosis or any other heart problem including congestive heart failure
Hypertension	… high blood pressure or hypertension?	… High blood pressure or hypertension
Stroke	… a stroke?	… A stroke or cereberal vascular disease
Diabetes	… diabetes or high blood sugar?	… Diabetes or high blood sugar
Lung Disease	… chronic lung disease such as chronic bronchitis or emphysema?	… Chronic lung disease such as chronic bronchitis or emphysema
Cancer	… cancer or a malignant tumor, excluding minor skin cancers?	… Cancer or malignant tumour, including leukaemia or lymphoma, but excluding minor skin cancers

Table 2

Condition	Prevalence (%) HRS (55-64)	NHIS (55-64)	MEPS (55-64)	HRS (65+)	NHIS (65+)	MCBS (65+)	MEPS (65+)	Description HRS	Description NHIS	Description MEPS	Description MCBS
Cancer	8%	8%	7%	16%	15%	18%	12%	Has a doctor ever told you that you have cancer or a malignant tymor, excluding minor skin cancers?	Have you ever been told be a doctor or other health professional that you had … cancer or a malignanacy of any kind? (WHEN RECODED, SKIN CANCERS WERE EXCLUDED)	List all the conditions that have bothered (the person) from (START time) to (END time) CCS codes for the conditions list are 11-21, 24-45	Has a doctor ever told you that you had any (other) kind of cancer malignancy, or tumor other than skin cancer?
Heart Diseases	14%	17%	16%	30%	31%	40%	33%	Has a doctor ever told you that you had a heart attack, coronary heart disease, angina, congestive heart failure, or other heart problems?	Four separate questions were asked about whether ever told by a docotor or oither health professional that had: CHD, Angina, MI, other heart problems.	have you ever been told by a doctor or health professional that you have … CHD; Angina; MI; other heart problems	Siz separate questions were asked about whether ever told by a doctor that had: Angina or MI; CHD; other heart problems (included four questions)
Stroke	4%	3%	4%	11%	9%	12%	11%	Has a doctor ever told you that you had a stroke?	Have you EVER been told by a doctor or other health professional that you had a stroke?	If Female, add: [Other than during pregnancy,] Have you ever been told by a doctor or health professional that you have a stroke or TIA (transient ischemic attack)	[Since (PREV< SUPP. RD. INT. DATE),] has a doctor (ever) told (you/SP) that (you/he/she) had a stroke, a bran hemorrhage, or a cerebrovascular accident?
Diabetes	14%	13%	14%	17%	15%	22%	19%	Has a doctor ever told you that you have diabetes or high blood sugar?	If Female, add: [Other than during pregnancy,] Have you ever been told by a doctor or health professional that you have diabetes or sugar diabetes?	If Female, add: [Other than during pregnancy,] Have you hever been told by a doctor or health professional that you have diabetes or sugar diabetes?	has a doctor (ever) told (you/SP) that (you/he/she) had diaebtes, high blood sugar, or sugar in (your/his/her) urine? [DO NOT INCLUDE BOERDERLINE PREGNANCY, OR PRE-DIABETEIC DIABETES.]
Hypertension	42%	42%	46%	56%	54%	64%	63%	Has a doctor ver told you that you have high blood pressure or hypertension?	Have you EVER been told by a doctor or other health professional that you had Hypertension, also called high blood pressure?	Have you EVER been told by a doctor or other health professional that you had Hypertension, also called high blood pressure?	has a doctor (ever) told (you/SP) that (you/he/she) (still) (had) (have/has) hypertension, sometimes called high blood pressure?

Table 2

Lung Disease	7%	8%	7%	10%	10%	16%	9%	Has a doctor ever told you that you have chronic lung disease such a schronic bronchitis or emphysema? [IWER: DO NOT INCLUDE ASTHMA]	Question 1: During the PAST 12 MONTHS, have you ever been told by a doctor or other health professional that you had chronic bronchitis? Question 2: Have you EVER been told by a docotor or other health professional that you had … emphysema?	List all the conditions that have bothered (the person) from (START time) to (END time) CCS codes for the conditions list are 127, 129-312	has a doctor (ever) told (you/SP) that (you/he/she) had emphysema, asthma, or COPD? [COPD=CHRONIC OBSTRUCTIVE PULMONARY DISEASE.]
Overweight	40%	38%	38%	38%	36%	38%	38%	self-reported body weight and height			
Obese	30%	31%	32%	20%	23%	23%	24%				

Table 3

	Data source	Projection method	Directly obtained from other sources
Chronic conditions	National Health Interview Survey 1997-2006	Assume no recovery	There are other forecasts (Honeycutt, 2003, Mainous 2007) for the trends of diabetes in the U.S population; we compare their forecasts to ours and they are reasonably close
	cohort-mortality rate from mortality.org	Use synthetic cohort approach to estimate age-specific incidence rate for each condition, using NHIS 1997-2006	
	Assumed annual mortality improvement rate for year 2005-2030: 0.8% per year. Assume relative risks of mortality for each condition: rr = 2 for cancer, diabetes and heart and rr = 1.5 for hypertension, lung and stroke	Baseline prevalence is obtained from the NHIS 2003-2005 pooled data	
Cancer		Use Markov model to model the transition into a certain condition or die from 2005 to 2030	
Diabetes			
Heart			
Hypertension			
Lung			
Stroke			
Over-weight and obese	Prevalence of over-weight and obese for aged 46-56 from year 2001 to 2030, generated by Ruhm upon request		Ruhm, Christopher J., "Current and Future Prevalence of Obesity and Severe Obesity in the United States", *Forum for Health Economics and Policy* , Vol. 10, No. 2 (Obesity), Article 6, 2007, 1-26.
Ever-smoked and smoking now	Status quo - Tobacco control policies will be frozen in place as of the beginning of 2006, with excise tax rates assumed to be adjusted for inflation.		Forecast of prevalence of ever-smoked and smoking now for aged 45-54 from year 2005 to 2025, by David
Any DB from current job	Prevalence of DB entitlement from current job among aged 50-55, in HRS 1992 and 2004		Historical trends of DB participation rates among all persons by different birth cohorts and by age, by Poberta 2007 (a)
Any DC from current job	Prevalence of DC entitlement from current job among aged 50-55, in HRS 1992 and 2004		Forecast of DC participation rates among all persons by different birth cohorts and by age, by Poberta 2007 (b)
Hispanic	Projection of population from US census Bureau, Interim projection consistent with 2000 census (2004), Projection of population from US census		
Non-Hispanic black	Since the interim projection consistent with 2000 census doesn't provide projection for all race/ethnicity categories, we cannot obtain the projection of		
Population size 50-52	non-Hispanic black population. As a result I turn to the final projection consistent with 1990 census and find out what proportion of the black		

Table 4

		Prevalence/Means relative to year 2004					
		2004	2010	2020	2030	2040	2050
Prevalence of binary outcomes	Hypertension	100%	104%	107%	109%	111%	113%
	Heart Disease	100%	95%	91%	88%	85%	83%
	Diabetes	100%	112%	122%	127%	131%	136%
Prevalence of highest category in ordered outcomes	BMI Status - obesity	100%	124%	172%	238%	303%	328%
	Smoking Status - smoking now	100%	94%	73%	60%	50%	41%
Prevalence of censored discrete outcomes	Any DB Plan	100%	89%	72%	59%	48%	39%
	Any DC Plan	100%	114%	141%	156%	156%	156%

Table 5

	1978	2004	Annual rate of change in prevalence rate from 1978 to 2004
Obesity (BMI >=30 kg/m^2)	15.70%	31.60%	-0.027
Hypertension (Self-reported)	29.60%	33.00%	-0.004
Diabetes (Self-reported)	4.80%	8.60%	-0.022
Current smokers	39.50%	26.20%	0.016

Prevalence in 1978 is based on NHANES II 1976-1980; Prevalence in 2004 is based on NHANES 2001-2006 pooled data.
BMI is calculated using self-reported weight and height

Table 6

			Type	mean/fraction	At risk
Disease	heart disease		biannual incidence	3.2%	undiagnosed
	hypertension		biannual incidence	4.2%	undiagnosed
	stroke		biannual incidence	1.6%	undiagnosed
	lung disease		biannual incidence	1.5%	undiagnosed
	cancer		biannual incidence	2.0%	undiagnosed
	diabetes		biannual incidence	2.1%	undiagnosed
Risk Factors	Smoking Status	never smoked	ordered	41.6%	all
		ex smoker	ordered	43.4%	all
		current smoker	ordered	15.0%	all
	Log BMI		continuous	3.28	all
	ADL Status	no ADLs	ordered	76.7%	all
		1 ADL	ordered	8.1%	all
		2 ADLS	ordered	3.6%	all
		3+ ADLS	ordered	5.5%	all
	IADL Status	no IADLs	ordered	83.2%	all
		1 IADL	ordered	5.8%	all
		2+ IADLs	ordered	4.5%	all
LFP & Benefits	working		prevalence	48.5%	age < 75
	DB pension receipt		biannual incidence	8.3%	eligible & not receiving
	SS benefit receipt		biannual incidence	7.0%	eligible & not receiving
	DI benefit receipt		prevalence	3.5%	eligible & age < 65
	Any health insurance		prevalence	88.5%	age < 65
	SSI receipt		prevalence	3.1%	all
	Nursing Home residency		prevalence	2.1%	all
	Death		biannual incidence	7.3%	all
Financial Resources $USD 2004	financial wealth		median	$ 162,354	all positive wealth
	earnings		median	$ 3,151	all working
	wealth positive		prevalence	96.3%	all

Table 7

	Outcome at time T																			
Value at time T-1	heart disease	hypertension	stroke	lung disease	diabetes	cancer	disability	mortality	smoking status	BMI	Any HI	DI Claim	SS Claim	DB Claim	SSI Claim	Nursing Home	Work	Earnings	Nonzero Wealth	Wealth
heart disease			X				X	X	X	X	X	X	X	X	X	X	X	X	X	X
blood pressure	X		X				X	X	X	X	X	X	X	X	X	X	X	X	X	X
stroke							X	X	X	X	X	X	X	X	X	X	X	X	X	X
lung disease							X	X	X	X	X	X	X	X	X	X	X	X	X	X
diabetes	X	X	X				X	X	X	X	X	X	X	X	X	X	X	X	X	X
cancer			X				X	X	X	X	X	X	X	X	X	X	X	X	X	X
disability							X	X	X	X	X	X	X	X	X	X	X	X	X	X
claimed DI											X	X	X	X	X		X	X	X	X
claimed SS											X			X	X		X	X	X	X
claimed DB													X		X		X	X	X	X
claimed SSI															X					
work											X	X	X		X		X	X	X	X
earnings											X	X	X	X	X		X	X	X	X
nonzero wealth											X	X	X	X	X	X	X	X	X	X
wealth											X	X	X	X	X	X	X	X	X	X
nursing home stay															X	X			X	X

Table 8

Control variable	Mean	Standard Deviation	Minimum	Maximum
				Unweighted Statistics
age	51.96	0.548	51	52.92
black	0.159	0.366	0	1
hispanic	0.147	0.355	0	1
less than high school	0.123	0.329	0	1
college education	0.596	0.491	0	1
male	0.465	0.499	0	1
ever smoked (includes current)	0.549	0.498	0	1
widowed at baseline	0.0222	0.147	0	1
single at baseline	0.298	0.457	0	1
log AIME/10 at baseline	0.7	0.141	0.288	1.132
log quarters/10 at baseline	0.426	0.0927	0.151	0.678
Any DB at baseline	0.23	0.421	0	1
NRA 57-61	0.0575	0.233	0	1
NRA 62-63	0.0363	0.187	0	1
NRA 64+	0.0949	0.293	0	1
Any DC at baseline	0.293	0.455	0	1
(IHT of DC wealth in 1000s)/100 if any DC, zero else	0.0111	0.0194	0	0.0996

Table 9

	heart	blood pressure	stroke	lung disease	diabetes	cancer	disability	mortality	Smoking	BMI
**Prevalence of Condition at t-1**										
heart			x				x	x	x	x
blood pressure	x		x				x	x	x	x
stroke							x	x	x	x
lung disease							x	x	x	x
diabetes	x	x	x				x	x	x	x
cancer			x				x	x	x	x
disability							x	x	x	x
Test of Restrictions										
Primary Variable(s) Responsible										
**Economics Outcomes at t-1**										
claimed DI										
claimed SS										
claimed DB										
work										
earnings										
wlth = 0										
wealth										
nursing home stay										
Test of Restrictions										
Primary Variable(s) Responsible										

Notes: x denotes a parameter which is allowed to be estimated. Other controls include initial conditions, demographics, db and dc plan characteristics, AIME and social security quarters of coverage. Tests do not include nursing home restriction or claimed DI

Table 10

	Mortality	Heart disease	Stroke	Cancer	Hypertension	Diabetes	Lung disease
Non-Hispanic black	-0.0316	-0.0463	0.0280	-0.125	0.0701	0.0970	-0.151
	(-0.82)	(-1.07)	(0.53)	(-2.34)	(1.48)	(2.14)	(-2.73)
Hispanic	-0.156	-0.131	-0.0753	-0.114	-0.0177	0.342	-0.154
	(-2.77)	(-2.25)	(-1.00)	(-1.58)	(-0.34)	(6.43)	(-2.03)
Less than high school	0.0645	0.0995	0.0556	-0.0271	0.0479	0.0928	0.0566
	(2.11)	(2.73)	(1.24)	(-0.62)	(1.30)	(2.32)	(1.27)
Some college and above	-0.0325	0.00560	-0.00962	0.0501	-0.0507	-0.0452	-0.0501
	(-1.40)	(0.22)	(-0.30)	(1.80)	(-2.14)	(-1.60)	(-1.57)
Male	0.220	0.209	0.109	0.152	-0.0777	0.126	-0.0838
	(8.64)	(7.60)	(3.01)	(5.04)	(-2.99)	(4.04)	(-2.39)
Male AND Less than high school	-0.0540	-0.0780	-0.0821	0.0821	0.00267	-0.0372	0.0542
	(-1.30)	(-1.51)	(-1.27)	(1.41)	(0.05)	(-0.66)	(0.85)
Male AND Non-Hispanic black	0.111	-0.157	0.00428	0.158	0.0348	0.0635	-0.0683
	(1.99)	(-2.31)	(0.05)	(2.09)	(0.47)	(0.90)	(-0.75)
Male AND Hispanic	0.179	-0.0558	0.00187	-0.0991	0.0888	-0.207	-0.0557
	(2.33)	(-0.65)	(0.02)	(-0.97)	(1.13)	(-2.48)	(-0.49)
Age Spline for Lag of Age <= 65	0.0370	0.0228	0.0185	0.0201	0.0125	0.0160	0.00732
	(6.81)	(5.40)	(3.02)	(4.23)	(3.78)	(3.85)	(1.46)
Age Spline for Lag of Age between 65 and 74	0.0210	0.0214	0.0244	0.0206	0.0120	0.00837	0.0271
	(5.96)	(5.74)	(4.98)	(5.00)	(3.30)	(2.03)	(5.74)
Age Spline for Lag of Age 75+	0.0489	0.0185	0.0254	-0.00724	-0.00422	-0.00865	-0.000672
	(24.05)	(6.02)	(7.25)	(-1.96)	(-1.22)	(-2.12)	(-0.16)
Lag of Heart disease	0.223		0.140				
	(10.94)		(4.46)				
Lag of Stroke	0.0889						
	(3.22)						
Lag of Cancer	0.376		-0.00501				
	(15.76)		(-0.12)				
Lag of Hypertension	0.107	0.163	0.144				
	(5.26)	(7.15)	(4.88)				
Lag of Diabetes	0.211	0.157	0.149		0.146		
	(8.65)	(4.90)	(3.92)		(3.85)		
Lag of Lung disease	0.346						
	(13.08)						
Lag of Has exactly 1 IADL	0.188						
	(5.74)						
Lag of Has 2 or more IADLs	0.571						
	(15.59)						
Lag of Has exactly 1 ADL	0.214						
	(7.35)						
Lag of Has exactly 2 ADLs	0.402						
	(10.65)						
Lag of Has 3 or more ADLs	0.636						
	(18.53)						
Lag of Current smoking	0.156	0.0913	0.143	0.0803	0.0695	0.00519	0.269

Table 10

	(5.02)	(2.49)	(2.97)	(1.95)	(1.93)	(0.12)	(6.65)
Lag of Widowed	0.154	0.0529	0.0421	0.0899	0.0720	-0.00368	0.0561
	(6.05)	(1.59)	(1.05)	(2.40)	(2.08)	(-0.09)	(1.35)
Init. of Heart disease	0.0631		0.260	0.0923	0.0644	0.202	0.136
	(0.92)		(2.69)	(1.02)	(0.66)	(2.38)	(1.30)
Init. of Stroke	-0.167	0.357		0.0121	0.0718	0.0355	0.530
	(-0.77)	(1.66)		(0.05)	(0.28)	(0.15)	(2.31)
Init. of Cancer	-0.189	0.112	0.0246		0.0262	-0.00829	0.270
	(-2.80)	(1.64)	(0.25)		(0.43)	(-0.11)	(3.78)
Init. of Hypertension	0.0885	0.0825	0.151	0.0248		0.211	-0.0918
	(1.50)	(1.48)	(2.13)	(0.37)		(3.99)	(-1.22)
Init. of Diabetes	0.0633	0.131	0.166	-0.0731	0.112		0.251
	(1.34)	(2.33)	(2.52)	(-1.11)	(1.75)		(4.23)
Init. of Lung disease	0.0101	0.390	0.197	0.229	0.0387	0.0521	
	(0.07)	(2.27)	(0.92)	(1.18)	(0.23)	(0.28)	
Init. of Ever smoked	0.0712	0.0463	0.00934	0.0563	0.000155	0.0135	0.272
	(2.84)	(1.68)	(0.27)	(1.85)	(0.01)	(0.44)	(7.11)
Init. of Current smoking	0.153	0.106	0.0222	0.00818	0.00348	0.0465	0.232
	(5.74)	(3.31)	(0.54)	(0.23)	(0.11)	(1.30)	(5.99)
Init. of Has exactly 1 IADL	0.0230	-0.101	0.141	-0.0507	-0.0423	0.123	-0.118
	(0.46)	(-1.51)	(1.89)	(-0.67)	(-0.62)	(1.75)	(-1.39)
Init. of Has 2 or more IADLs	0.173	0.0252	0.116	0.0643	0.0263	-0.00761	0.0767
	(6.41)	(0.79)	(2.92)	(1.80)	(0.85)	(-0.21)	(1.96)
Init. of Has exactly 1 ADL		-0.00700	-0.236	-0.134	0.818	1.150	0.000441
		(-0.05)	(-1.38)	(-0.85)	(5.90)	(6.52)	(0.00)
Init. of Has exactly 2 ADLs		0.340	-0.128	0.218	0.372	1.173	0.985
		(1.82)	(-0.56)	(0.99)	(1.73)	(6.59)	(4.44)
Init. of Has 3 or more ADLs		0.170	0.185	0.120	0.00698	0.711	-0.101
		(1.27)	(1.12)	(0.80)	(0.05)	(4.42)	(-0.61)
Init. of Widowed		0.169	0.399	-0.0614	0.0805	0.141	0.110
		(0.86)	(1.69)	(-0.26)	(0.34)	(0.73)	(0.45)
Init. of Single		0.243	0.453	0.401	0.248	0.395	0.172
		(2.98)	(4.29)	(4.35)	(3.22)	(4.37)	(1.69)
Init. of R working for pay	-4.904	-4.210	-3.839	-3.613	-4.936	-9.399	-2.778
	(-14.89)	(-10.29)	(-7.03)	(-8.09)	(-13.66)	(-19.52)	(-5.71)
IHT(Init. of Individual earnings in 1000s-max 200)/100	-0.652	-0.617	-1.801	2.079	-0.670	-1.516	-1.426
	(-0.50)	(-0.50)	(-1.09)	(1.54)	(-0.60)	(-1.15)	(-0.89)
Init. of Non-pension wlth(hatota) not zero	0.00695	0.0525	0.0937	-0.0745	0.0268	0.0445	0.0133
	(0.13)	(0.76)	(1.10)	(-0.91)	(0.35)	(0.62)	(0.16)
IHT(Init. of HH wlth in 1000s if positive-max 2000 zero otherwise)/100	-1.052	-1.067	-1.813	1.755	-1.135	-1.868	-1.844
	(-2.17)	(-1.91)	(-2.64)	(2.60)	(-2.14)	(-3.17)	(-2.79)
Init. of Health fair/poor	0.103	0.130	0.0548	-0.0180	-0.00971	0.120	0.177
	(4.31)	(4.23)	(1.48)	(-0.51)	(-0.29)	(3.60)	(4.88)
Init. of Any DB from current job RND VG	0.202	-0.295	0.0623	0.0774	0.00831	0.0237	-0.0344

Table 10

	(2.24)	(-2.91)	(0.56)	(0.94)	(0.12)	(0.28)	(-0.29)
Init. of Normal DB Retirement Age 60-61	-0.0685	0.369	-0.279	-0.149	-0.0980	-0.0200	-0.131
	(-0.61)	(3.21)	(-1.79)	(-1.41)	(-1.12)	(-0.19)	(-0.87)
Init. of Normal DB Retirement Age 62-64	-0.0643	0.283	-0.0345	-0.0755	0.0542	0.0496	0.146
	(-0.54)	(2.26)	(-0.23)	(-0.67)	(0.58)	(0.45)	(1.01)
Init. of Normal DB Retirement Age 65+	-0.0760	0.341	-0.0723	-0.0956	0.0226	-0.0517	0.107
	(-0.73)	(3.07)	(-0.55)	(-0.99)	(0.28)	(-0.52)	(0.81)
Init. of Any DC from current job RND VG	0.0368	0.0388	0.120	0.0501	0.127	0.0710	0.0469
	(0.43)	(0.53)	(1.17)	(0.62)	(1.97)	(0.94)	(0.48)
Init. of (IHT of DC wlth in 1000s)/100 if any DC zero otherwise	-2.559	-0.807	-4.687	-2.410	-2.679	-2.528	-2.039
	(-1.20)	(-0.46)	(-1.79)	(-1.25)	(-1.71)	(-1.34)	(-0.83)
Splined lag of BMI <= log(30)		-0.00721	-0.213	-0.194	0.865	1.100	-0.104
		(-0.05)	(-1.09)	(-1.08)	(5.51)	(5.49)	(-0.53)
Splined lag of BMI > log(30)		0.175	-0.488	0.133	0.401	1.241	1.070
		(0.86)	(-1.90)	(0.56)	(1.76)	(6.44)	(4.34)
Splined init of BMI <= log(30)		0.229	0.164	0.183	0.00766	0.891	-0.0285
		(1.38)	(0.80)	(0.99)	(0.05)	(4.45)	(-0.14)
Splined init of BMI > log(30)		0.204	0.747	0.0925	0.0806	0.0236	-0.241
		(0.93)	(2.84)	(0.36)	(0.32)	(0.11)	(-0.87)
Log of years between current interview and previous		0.249	0.461	0.404	0.243	0.402	0.173
		(3.04)	(4.35)	(4.37)	(3.15)	(4.43)	(1.68)
Constant	-4.740	-4.228	-3.590	-3.651	-5.098	-9.604	-2.601
	(-14.42)	(-9.56)	(-6.17)	(-7.53)	(-12.83)	(-17.78)	(-4.89)

t statistics in parentheses

Table 11

	HI cov - gov/emp/other	Claiming SSDI	Claiming DB	Claiming SSI	R live in nursing home at interview	Non-pension wlth(hatota) not zero	Claiming OASI	R working for pay
Non-Hispanic black	-0.185	0.127	0.144	0.322	-0.345	-0.622	-0.216	0.0539
	(-3.26)	(1.78)	(1.33)	(3.86)	(-4.74)	(-15.12)	(-2.93)	(1.32)
Hispanic	-0.525	0.00749	-0.157	0.184	-0.576	-0.677	-0.138	-0.112
	(-8.53)	(0.08)	(-0.88)	(1.64)	(-5.01)	(-13.84)	(-1.43)	(-1.99)
Less than high school	-0.281	-0.114	-0.0347	0.0575	-0.0673	-0.343	0.0527	-0.0878
	(-5.53)	(-1.60)	(-0.25)	(0.71)	(-1.23)	(-8.55)	(0.75)	(-2.33)
Some college and above	0.224	-0.112	-0.164	-0.0922	0.0618	0.0765	-0.198	0.101
	(5.64)	(-2.20)	(-2.73)	(-1.15)	(1.37)	(1.61)	(-4.34)	(4.50)
Male	-0.345	0.0474	0.178	0.267	0.0264	-0.251	-0.299	0.177
	(-6.73)	(0.72)	(2.58)	(2.71)	(0.46)	(-4.10)	(-5.29)	(7.13)
Male x Less than high school	-0.0438	0.0386	0.00785	0.0498	-0.0510	0.113	-0.0870	0.0750
	(-0.55)	(0.36)	(0.04)	(0.41)	(-0.61)	(1.70)	(-0.88)	(1.45)
Male x Black	0.462	0.00438	-0.0102	-0.496	0.255	0.112	0.254	-0.111
	(4.63)	(0.04)	(-0.06)	(-3.43)	(2.20)	(1.52)	(2.25)	(-1.78)
Male x Hispanic	0.154	-0.245	0.224	-0.0791	0.177	0.0640	0.159	0.0494
	(1.55)	(-1.54)	(0.90)	(-0.45)	(0.98)	(0.77)	(1.15)	(0.62)
Spline Lag of Age <= 65	-0.00987		0.129	-0.000341	0.0611			
	(-2.07)		(13.02)	(-0.03)	(3.61)			
Lag of Heart disease	0.310	0.126	0.0404	0.0493	-0.0203	0.0165	0.0486	-0.0260
	(5.09)	(2.23)	(0.49)	(0.74)	(-0.51)	(0.47)	(0.86)	(-0.91)
Lag of Stroke	0.0269	-0.0485	-0.0164	-0.0255	0.119	-0.0951	-0.166	-0.131
	(0.27)	(-0.53)	(-0.09)	(-0.28)	(2.48)	(-2.13)	(-1.74)	(-2.44)
Lag of Cancer	0.264	0.211	-0.139	0.0632	-0.0499	0.182	-0.0478	-0.0753
	(2.99)	(2.32)	(-1.23)	(0.66)	(-0.97)	(3.46)	(-0.62)	(-1.97)
Lag of Hypertension	-0.00251	0.113	0.114	0.0131	0.0167	-0.00541	0.0449	-0.0753
	(-0.07)	(2.32)	(2.02)	(0.21)	(0.43)	(-0.17)	(1.10)	(-3.52)
Lag of Diabetes	0.0393	0.152	-0.0342	-0.0188	0.190	0.0826	0.0744	-0.0883
	(0.64)	(2.28)	(-0.39)	(-0.24)	(3.98)	(2.13)	(1.20)	(-2.64)
Lag of Lung disease	0.142	0.0543	-0.282	0.185	-0.0473	0.0548	-0.0826	-0.146
	(1.82)	(0.72)	(-2.05)	(2.28)	(-0.79)	(1.14)	(-1.09)	(-3.36)
Lag of Has exactly 1 IADL	0.109	0.221	0.222	0.127	0.451	-0.139	0.0346	-0.255
	(1.17)	(2.64)	(1.26)	(1.34)	(8.53)	(-2.96)	(0.32)	(-3.78)
Lag of Has 2 or more IADLs	0.328	-0.0351	-0.332	0.0427	0.871	-0.122	-0.0372	-0.224
	(1.96)	(-0.27)	(-0.62)	(0.36)	(14.89)	(-2.02)	(-0.20)	(-1.62)
Lag of Has exactly 1 ADL	0.143	0.416	0.150	0.0892	0.238	-0.0658	-0.154	-0.109
	(2.06)	(6.30)	(1.08)	(1.09)	(4.60)	(-1.53)	(-1.94)	(-2.51)
Lag of Has exactly 2 ADLs	-0.0309	0.378	0.305	-0.0700	0.471	-0.120	-0.0310	-0.277
	(-0.31)	(4.22)	(1.23)	(-0.66)	(7.38)	(-2.15)	(-0.25)	(-3.49)
Lag of Has 3 or more ADLs	0.257	0.580	0.673	0.101	0.500	-0.0824	-0.176	-0.545
	(2.20)	(6.56)	(2.65)	(1.02)	(8.27)	(-1.58)	(-1.45)	(-5.24)
Lag of Widowed	-0.165	0.202	-0.268	0.0415	0.232	-0.302	-0.0265	0.0556
	(-1.83)	(1.84)	(-1.69)	(0.44)	(4.97)	(-7.30)	(-0.28)	(1.41)
Lag of R working for pay	-0.373	-0.352		0.233		0.145	0.0419	1.800
	(-5.47)	(-3.25)		(1.74)		(1.73)	(0.44)	(50.78)
Lag of (IHT of earnings in 1000s)/100 if working zero otherwise	10.71	-7.543	23.15	-13.27			-18.12	7.543
	(6.39)	(-2.73)	(11.70)	(-3.07)			(-8.11)	(8.27)
Lag of Non-pension wlth(hatota) not zero	-0.119	0.0107	-0.298	-0.00798	-0.0974		-0.130	0.464
	(-1.31)	(0.10)	(-0.87)	(-0.08)	(-1.27)		(-1.13)	(5.16)
Lag of (IHT of hh wlth in 1000s if positive)/100 zero otherwise	7.083	-2.412	2.918	-4.885	-5.274	20.77	1.605	-1.894
	(9.98)	(-2.75)	(1.79)	(-4.18)	(-6.41)	(40.09)	(1.65)	(-3.62)
l2diclaim	1.046	2.851	-0.158	0.442			-1.014	-0.444
	(10.25)	(50.88)	(-0.60)	(5.26)			(-14.01)	(-6.47)
l2ssclaim	-0.0665		0.138	-0.00226				-0.0401
	(-1.08)		(1.51)	(-0.02)				(-1.15)
Init. of Heart disease	-0.204	0.194	0.519	-0.151	-0.186	0.226	-0.156	-0.174
	(-1.73)	(1.70)	(2.18)	(-0.97)	(-0.86)	(1.90)	(-1.24)	(-2.03)
Init. of Stroke	-0.0468	-0.0758	1.110	0.0349	0.267	0.206	0.283	-0.369
	(-0.20)	(-0.37)	(1.51)	(0.10)	(0.61)	(0.94)	(0.45)	(-1.25)
Init. of Cancer	-0.294	-0.0211	0.298	0.165	-0.108	-0.0412	-0.0205	0.0376
	(-2.80)	(-0.18)	(1.65)	(1.05)	(-0.63)	(-0.41)	(-0.17)	(0.56)
Init. of Hypertension	0.0183	-0.0178	0.0992	0.0484	0.0810	0.0287	-0.0281	0.0151
	(0.29)	(-0.25)	(0.82)	(0.39)	(0.57)	(0.39)	(-0.24)	(0.29)
Init. of Diabetes	0.00820	0.0445	0.0606	-0.108	0.186	-0.0391	-0.0107	-0.0430
	(0.10)	(0.52)	(0.41)	(-0.90)	(2.03)	(-0.61)	(-0.11)	(-0.76)
Init. of Lung disease	-0.438	0.227	0.928	-0.292	0	-0.464	-0.280	-0.118

Table 11

	(-2.58)	(1.36)	(1.93)	(-0.90)	(.)	(-2.72)	(-0.56)	(-0.62)
Init. of Ever smoked	0.187	0.0503	0.0340	-0.0425	-0.0000395	0.00550	0.0669	-0.0686
	(4.11)	(0.84)	(0.53)	(-0.54)	(-0.00)	(0.14)	(1.37)	(-2.75)
Init. of Current smoking	-0.284	0.153	0.0791	0.104	0.0253	-0.101	-0.00192	-0.00627
	(-6.25)	(2.71)	(1.18)	(1.35)	(0.51)	(-2.46)	(-0.04)	(-0.24)
fwidowed50	0.0974	-0.302	0.193	-0.00654	0.104	-0.169	-0.0370	-0.0925
	(0.79)	(-1.98)	(0.85)	(-0.05)	(1.26)	(-2.96)	(-0.26)	(-1.33)
fsingle50	-0.161	-0.0149	0.0167	0.171	0.266	-0.428	-0.0643	0.0438
	(-3.63)	(-0.26)	(0.22)	(2.36)	(4.88)	(-11.47)	(-1.20)	(1.47)
logdeltaage	-0.0663	-0.0210	0.297	0.0647	1.194	-0.0976	0.505	-0.238
	(-0.56)	(-0.13)	(1.47)	(0.31)	(8.39)	(-0.89)	(3.28)	(-3.04)
fraime	0.000257	-0.000112	0.00000344	-0.0000242	-0.0000240	0.000184	-0.0000589	
	(10.63)	(-3.78)	(0.12)	(-0.46)	(-0.79)	(5.76)	(-2.34)	
frq	-0.00259	0.00612	-0.00314	-0.00361	-0.000324	0.00140	0.00917	
	(-4.20)	(7.33)	(-3.07)	(-3.20)	(-0.51)	(2.56)	(12.67)	
nraplus10		0.411						
		(3.69)						
nraplus9		0.591						
		(4.63)						
nraplus8		0.392						
		(3.04)						
nraplus7		0.498						
		(3.94)						
nraplus6		0.684						
		(5.81)						
nraplus5		0.423						
		(3.46)						
nraplus4		0.584						
		(5.10)						
nraplus3		0.654						
		(5.80)						
nraplus2		0.554						
		(5.00)						
nraplus1		0.418						
		(3.71)						
l2age6574			-0.0944	-0.0209	0.0430			
			(-4.28)	(-1.61)	(5.36)			
l2age75p			0.377	-0.00976	0.0483			
			(2.59)	(-1.01)	(13.69)			
l2ssiclaim				2.990				
				(48.81)				
l2dbclaim				-0.0886			-0.0810	0.0337
				(-0.51)			(-1.05)	(0.85)
l2nhmliv					2.083	-0.757		
					(26.73)	(-9.94)		
l2a6						-0.0227		
						(-2.21)		
l2a7						-0.00470		
						(-1.24)		
l2a7p						-0.0206		
						(-6.16)		
w5						0.0290		
						(0.63)		
w6						0.0237		
						(0.50)		
w7						0.0738		
						(1.56)		
w8						0.00434		
						(0.09)		
l2logiearnuc						7.106		
						(2.74)		
at_eea							0.435	-0.224
							(7.01)	(-6.41)
at_nra							1.423	-0.0911
							(21.48)	(-2.48)
yrs_before_nra							-0.234	0.0420
							(-8.19)	(9.39)
yrs_after_nra							-0.0990	-0.0379
							(-8.13)	(-10.04)

Table 11

unemployment								0.00652
								(0.51)
Constant	1.637	-2.830	-9.432	-2.361	-7.367	2.931	-0.511	-1.497
	(5.59)	(-14.49)	(-13.79)	(-3.61)	(-7.05)	(5.05)	(-2.92)	(-12.35)

t statistics in parentheses

Table 12

	Log(BMI)
Non-Hispanic black	-0.000279
	(0.58)
Hispanic	-0.00149
	(-0.88)
Less than high school	-0.00100
	(-0.88)
Some college and above	-0.000275
	(-0.36)
Male	0.000583
	(0.68)
Male AND Less than high school	0.00114
	(0.69)
Male AND Non-Hispanic black	-0.00683
	(-3.22)
Male AND Hispanic	0.000732
	(0.28)
Age Spline for Lag of Age <= 65	0.000480
	(2.92)
Age Spline for Lag of Age between 65 and 74	-0.000134
	(-0.84)
Age Spline for Lag of Age 75+	-0.00144
	(-9.13)
Lag of Heart disease	0.0000125
	(0.01)
Lag of Stroke	-0.00325
	(-2.40)
Lag of Cancer	0.000883
	(0.80)
Lag of Hypertension	0.00258
	(3.54)
Lag of Diabetes	0.00110
	(1.05)

Table 12

Lag of Lung disease	-0.00264<br>(-2.09)
Lag of Has exactly 1 IADL	-0.00291<br>(-1.80)
Lag of Has 2 or more IADLs	-0.00324<br>(-1.40)
Lag of Has exactly 1 ADL	0.00126<br>(1.00)
Lag of Has exactly 2 ADLs	-0.00105<br>(-0.55)
Lag of Has 3 or more ADLs	-0.00181<br>(-0.93)
Lag of Current smoking	-0.00960<br>(-8.05)
Lag of Widowed	0.000286<br>(0.26)
Init. of Heart disease	0.00213<br>(0.77)
Init. of Stroke	0.0119<br>(1.71)
Init. of Cancer	0.00232<br>(1.04)
Init. of Hypertension	0.00337<br>(1.97)
Init. of Diabetes	-0.00232<br>(-1.28)
Init. of Lung disease	0.000139<br>(0.03)
Init. of Ever smoked	0.000741<br>(0.88)
Init. of Current smoking	0.00272<br>(2.67)
Init. of Widowed	-0.00113<br>(-0.55)

Table 12

Init. of Single	-0.00136
	(-1.37)
Splined lag of BMI <= log(30)	0.816
	(186.48)
Splined lag of BMI > log(30)	0.822
	(135.39)
Splined init of BMI <= log(30)	0.136
	(32.53)
Splined init of BMI > log(30)	0.107
	(16.65)
Log of years between current interview and previous	-0.0121
	(-4.88)
frbyr	0.000726
	(6.13)
Constant	-1.258
	(-5.31)

t statistics in parentheses

Table 13

	ADL Status	IADL Status	Smoking status
Non-Hispanic black	0.0805	0.124	-0.0623
	(3.13)	(3.87)	(-2.63)
Hispanic	0.118	0.115	-0.230
	(3.58)	(2.84)	(-7.45)
Less than high school	0.121	0.160	-0.0389
	(5.55)	(5.92)	(-1.90)
Some college and above	-0.0527	-0.0796	0.0290
	(-3.09)	(-3.69)	(2.13)
Male	-0.0630	0.0944	0.404
	(-3.29)	(3.94)	(27.03)
Male AND Less than high school	-0.0244	0.0568	0.157
	(-0.75)	(1.48)	(5.25)
Male AND Non-Hispanic black	0.0694	-0.0630	-0.132
	(1.66)	(-1.25)	(-3.42)
Male AND Hispanic	0.0244	-0.0650	0.200
	(0.47)	(-1.05)	(4.28)
Age Spline for Lag of Age <= 65	0.00434	-0.00729	0.00698
	(1.58)	(-2.07)	(3.42)
Age Spline for Lag of Age between 65 and 74	0.0186	0.0337	-0.00740
	(7.39)	(10.63)	(-3.54)
Age Spline for Lag of Age 75+	0.0429	0.0435	-0.00954
	(22.48)	(20.19)	(-4.99)
Lag of Heart disease	0.138	0.0966	0.0859
	(8.35)	(4.82)	(5.56)
Lag of Stroke	0.284	0.288	0.0151
	(12.22)	(10.84)	(0.62)
Lag of Cancer	0.0293	0.0105	0.0764
	(1.33)	(0.40)	(3.87)
Lag of Hypertension	0.0525	0.0263	0.00758
	(3.42)	(1.39)	(0.58)
Lag of Diabetes	0.134	0.122	0.0138
	(6.78)	(4.98)	(0.73)

Table 13

Lag of Lung disease	0.230	0.0819	0.255
	(10.07)	(2.88)	(10.91)
Lag of Has exactly 1 IADL	0.323	1.047	0.00602
	(12.25)	(39.86)	(0.21)
Lag of Has 2 or more IADLs	0.648	1.890	-0.0125
	(17.83)	(49.89)	(-0.30)
Lag of Has exactly 1 ADL	1.022	0.323	0.0393
	(51.47)	(12.22)	(1.72)
Lag of Has exactly 2 ADLs	1.459	0.505	0.0291
	(51.48)	(14.15)	(0.84)
Lag of Has 3 or more ADLs	2.059	0.627	-0.0310
	(67.09)	(18.28)	(-0.87)
Lag of Current smoking	0.0788	0.121	2.513
	(3.21)	(3.92)	(97.50)
Lag of Widowed	0.0472	0.0437	0.00477
	(2.26)	(1.74)	(0.25)
Init. of Heart disease	0.00919	0.0419	0.0400
	(0.17)	(0.64)	(0.81)
Init. of Stroke	-0.108	0.0987	0.393
	(-0.87)	(0.70)	(3.20)
Init. of Cancer	0.0832	-0.0101	0.0555
	(1.84)	(-0.17)	(1.40)
Init. of Hypertension	0.0181	0.158	0.0219
	(0.51)	(3.56)	(0.73)
Init. of Diabetes	0.127	0.194	0.0660
	(3.80)	(4.77)	(2.05)
Init. of Lung disease	0.0246	-0.133	-0.230
	(0.26)	(-1.00)	(-2.34)
Init. of Ever smoked	0.00513	0.0287	
	(0.28)	(1.31)	
Init. of Current smoking	0.0735	-0.0568	2.776
	(3.47)	(-2.17)	(57.12)
Init. of Widowed	0.0919	0.102	0.0190
	(2.38)	(2.19)	(0.51)

Table 13

Init. of Single	0.0523	0.108	0.0384
	(2.55)	(4.32)	(2.15)
Splined lag of BMI <= log(30)	-0.384	-0.769	-0.137
	(-4.43)	(-7.68)	(-1.73)
Splined lag of BMI > log(30)	0.866	-0.403	0.303
	(8.06)	(-2.72)	(2.80)
Splined init of BMI <= log(30)	0.591	0.445	0.0961
	(6.97)	(4.47)	(1.28)
Splined init of BMI > log(30)	0.263	0.262	-0.460
	(2.31)	(1.76)	(-4.01)
Log of years between current interview and previous	0.180	0.251	-0.0206
	(3.43)	(3.89)	(-0.47)
cut1			
Constant	2.826	0.857	0.703
	(10.71)	(2.64)	(3.31)
cut2			
Constant	3.452	1.545	4.793
	(13.08)	(4.76)	(21.94)
cut3			
Constant	3.903		
	(14.78)		

t statistics in parentheses

Table 14

	Household Wealth if nonzero	Individual earnings if working
Non-Hispanic black	-3.882	0.0643
	(-16.57)	(0.66)
Hispanic	-3.924	-0.480
	(-10.16)	(-2.30)
Less than high school	-3.063	-0.208
	(-12.02)	(-1.37)
Some college and above	4.344	0.777
	(25.68)	(9.86)
Male	-1.103	0.293
	(-5.13)	(3.02)
Male AND Less than high school	0.714	0.00795
	(1.95)	(0.04)
Male AND Non-Hispanic black	0.0672	0.234
	(0.14)	(1.05)
Male AND Hispanic	-0.622	0.634
	(-1.07)	(2.20)
Age Spline for Lag of Age <= 65	0.347	-0.0519
	(8.35)	(-4.86)
Age Spline for Lag of Age between 65 and 74	0.154	-0.0515
	(8.13)	(-2.57)
Age Spline for Lag of Age 75+	-0.184	-0.0785
	(-8.04)	(-2.28)
Lag of Heart disease	-0.713	-0.385
	(-3.80)	(-3.44)
Lag of Stroke	-1.814	-0.0909
	(-6.07)	(-0.38)
Lag of Cancer	0.859	0.124
	(3.59)	(0.83)
Lag of Hypertension	-0.977	-0.0484
	(-6.26)	(-0.62)
Lag of Diabetes	-2.030	-0.140
	(-9.06)	(-1.05)

Table 14

Lag of Lung disease	-1.902	-0.0209
	(-6.89)	(-0.11)
Lag of Has exactly 1 IADL	-0.798	-0.137
	(-2.22)	(-0.48)
Lag of Has 2 or more IADLs	-1.277	-2.808
	(-2.34)	(-3.56)
Lag of Has exactly 1 ADL	-1.754	-0.282
	(-6.30)	(-1.56)
Lag of Has exactly 2 ADLs	-1.576	-0.468
	(-3.67)	(-1.22)
Lag of Has 3 or more ADLs	-1.710	-0.293
	(-3.80)	(-0.52)
Lag of Widowed	-4.401	0.723
	(-18.46)	(4.44)
Lag of R working for pay	0.188	-1.068
	(0.55)	(-6.70)
Lag of (IHT of earnings in 1000s)/100 if working zero otherwise	767.2	1.484
	(209.26)	(0.81)
Lag of Non-pension wlth(hatota) not zero		0.227
		(0.55)
Lag of Claiming SSDI		-2.010
		(-4.49)
Lag of Claiming OASI		-1.516
		(-11.72)
Lag of Claiming DB		-1.336
		(-7.53)
R live in nursingh ome at interview	-2.508	
	(-2.66)	
Init. of Heart disease	-0.807	0.0989
	(-1.34)	(0.29)
Init. of Stroke	0.700	-0.0404
	(0.45)	(-0.04)
Init. of Cancer	-0.741	-0.161

Table 14

	(-1.54)	(-0.70)
Init. of Hypertension	-0.505	-0.318
	(-1.36)	(-2.03)
Init. of Diabetes	-0.617	-0.209
	(-1.55)	(-1.03)
Init. of Lung disease	-0.340	-0.173
	(-0.28)	(-0.28)
Init. of Ever smoked	-0.0874	-0.0355
	(-0.48)	(-0.41)
Init. of Current smoking	-1.859	-0.127
	(-9.74)	(-1.40)
Init. of Has exactly 1 IADL	-0.440	-0.230
	(-0.94)	(-0.85)
Init. of Has 2 or more IADLs	-4.100	0.185
	(-18.38)	(1.80)
Indicator for HRS Wave 5	0.00343	
	(0.01)	
Indicator for HRS Wave 6	-0.220	
	(-0.90)	
Indicator for HRS Wave 7	0.468	
	(1.96)	
Indicator for HRS Wave 8	0.812	
	(3.29)	
Log(Time Between Interviews)	0.245	0.00828
	(0.43)	(0.03)
AIME in ini.intw (-9=no match)	0.00278	0.00130
	(28.49)	(31.82)
Quarters of earnings in ini.intw (-9=no match)	-0.0540	-0.0192
	(-21.49)	(-14.78)
Constant	-9.768	7.904
	(-4.17)	(10.62)

t statistics in parentheses

Table 15

	1992 Observed	2004 Observed	2004 Simulated
Survival	100%	88%	96%
Cancer Prevalence	5%	16%	16%
Diabetes Prevalence	10%	18%	19%
Heart Disease Prevalence	12%	29%	26%
Hypertension Prevalence	35%	56%	58%
Lung Disease Prevalence	6%	10%	14%
Stroke Prevalence	3%	11%	8%
Any Condition Prevalence	49%	80%	77%
3+ Conditions Prevalence	5%	32%	17%
Any IADLs Prevalence	12%	29%	4%
Any ADLs Prevalence	12%	35%	12%
Overweight Prevalence	41%	31%	36%
Obesity Prevalence	22%	36%	29%
Ever Smoked Prevalence	64%	57%	61%
Current Smoking Prevalence	29%	11%	15%
Working Prevalence	62%	20%	27%
OASI Claiming	5%	75%	82%
SSDI Claiming	8%	2%	3%
SSI Claiming	7%	2%	4%
Mean Earnings (thousands)	$ 14.90	$ 7.70	$ 8.29
Median HH wealth (thousands)	$ 131.93	$ 153.00	$ 208.66

Table 16

			1992	2004	Selection
Binary		working for pay	0.75	0.79	all
		non-zero wealth	0.97	0.98	all
		hypertension	0.30	0.36	all
		heate disease	0.09	0.09	all
		diabetes	0.07	0.11	all
		any health insurance	0.87	0.90	all
		SRH fair or poor	0.17	0.19	all
Ordered	BMI Status	normal	0.36	0.28	all
		overweight	0.41	0.38	all
		obese	0.24	0.35	all
	Smoking Status	never smoked	0.36	0.43	all
		former smoker	0.35	0.32	all
		current smoker	0.29	0.25	all
	Functional Status	no ADL	0.91	0.91	all
		no IADL	0.90	0.94	all
Continuous		aime ($USD)	1923	2023	all
		quarters of coverage	0.430	0.429	all
Censored Continuous		earnings	40030	42910	if working
		wealth	254137	286680	if non-zero
		dc wealth	17.07	26.58	if dc plan
Censored Discrete		any db plan	0.29	0.30	if working
		any dc plan	0.26	0.26	if working
Censored Ordered	Early Age Eligible DB	<52	0.21	0.23	
		52-57	0.58	0.58	
		58>	0.22	0.20	
	Normal Age Eligible DB	<57	0.18	0.23	
		57-61	0.26	0.29	
		62-63	0.17	0.17	
		64>	0.39	0.32	
Covariates		hispanic	0.07	0.09	all
		black	0.09	0.11	all
		male	0.47	0.49	all
		less high school	0.21	0.09	all
		college	0.40	0.63	all
		single	0.18	0.26	all
		widowed	0.04	0.02	all
		cancer	0.04	0.05	all
		lunge disease	0.05	0.04	all
		stroke	0.02	0.02	all

Table 17

covariate	Hypertension	Heart Disease	Diabetes	Any Health Insurance	Self-repoted Health	Weight Status
black	0.524	0.000	0.399	-0.149	0.515	0.356
hispan	-0.001	-0.169	0.311	-0.695	0.482	0.189
hsless	0.107	0.111	0.250	-0.514	0.472	0.097
college	-0.052	-0.082	-0.026	0.180	-0.376	-0.149
male	0.089	0.250	0.042	0.003	0.026	0.108
single	0.182	-0.006	0.070	-0.231	0.210	-0.032
widowed	0.151	0.049	0.037	-0.394	0.381	0.160
lunge	0.139	0.667	0.422	-0.075	1.035	0.046
cancre	-0.001	0.277	0.073	0.293	0.568	-0.135
stroke	0.937	0.946	0.557	-0.117	1.014	0.157
constant	-0.705	-1.519	-1.716	1.337	-1.222	0.304

covariate	Smoking Status	Function Status	Working	Nonzero Wealth	Log(AIME)	Log(Quarters Worked)
black	-0.107	0.325	-0.128	-0.953	-0.021	-0.007
hispan	-0.325	0.271	-0.161	-0.886	-0.063	-0.036
hsless	0.305	0.297	-0.346	-0.294	-0.056	-0.032
college	-0.105	-0.292	0.267	0.636	0.012	-0.004
male	0.419	-0.094	0.505	-0.037	0.132	0.074
single	0.268	0.050	0.065	-1.083	0.020	0.013
widowed	0.260	0.029	0.037	-1.149	-0.010	-0.008
lunge	0.633	0.704	-0.471	0.083	-0.006	-0.002
cancre	0.228	0.376	-0.205	0.134	0.007	0.004
stroke	0.127	0.771	-0.940	-0.562	-0.028	-0.022
constant	0.097	-1.049	0.485	2.821	0.652	0.405

covariate	IHT(HH Wealth)	IHT(Earned Income)	Log(DC Wealth)	Any DC Plan	Any DB Plan	Early Retirement Age	Normal Retirement Age
black	-16.850	-0.410	-0.028	-0.033	0.044	-0.134	-0.015
hispan	-15.690	-2.397	-0.041	-0.295	-0.193	0.071	0.081
hsless	-12.351	-2.718	-0.050	-0.279	-0.275	0.116	-0.042
college	11.191	4.543	0.087	0.256	0.088	-0.210	-0.337
male	-2.142	6.109	0.109	0.161	0.046	-0.106	0.054
single	-22.318	0.528	-0.021	0.063	-0.022	-0.065	0.054
widowed	-16.644	0.404	-0.086	0.123	-0.049	-0.074	0.074
lunge	-14.211	-1.379	-0.017	-0.103	0.094	0.071	-0.002
cancre	0.326	1.175	-0.044	0.044	-0.152	0.281	0.004
stroke	-12.300	-0.068	0.130	-0.032	0.000	0.000	0.000
constant	60.866	13.243	0.606	-0.588	-0.296	0.981	1.047

Table 18

	Hypertension	Heart Disease	Diabetes	Any Health Insurance	Self-repoted Health	Weight Status	Smoking Status	Function Status	Working	Nonzero Wealth	Log(AIME)	Log(Quarters Worked)	IHT(HH Wealth)	IHT(Earned Income)	Log(DC Wealth)	Any DC Plan	Any DB Plan	Early Retirement Age	Normal Retirement Age
Hypertension	1																		
Heart Disease	0.30	1																	
Diabetes	0.32	0.24	1																
Any Health Insurance	-0.03	0.03	0.00	1															
Self-repoted Health	0.34	0.50	0.40	-0.07	1														
Weight Status	0.29	0.12	0.24	0.00	0.19	1													
Smoking Status	-0.01	-0.02	0.00	-0.07	0.06	-0.10	1												
Function Status	0.15	0.23	0.17	-0.03	0.38	0.11	-0.01	1											
Working	-0.16	-0.27	-0.19	0.16	-0.39	-0.01	-0.04	-0.35	1										
Nonzero Wealth	-0.17	-0.13	-0.09	0.17	-0.16	-0.03	-0.05	-0.11	0.35	1									
Log(AIME)	0.00	-0.01	-0.01	0.02	-0.02	0.00	0.01	-0.02	0.05	0.03	0.02								
Log(Quarters Worked)	0.00	-0.01	-0.01	0.01	-0.01	0.00	0.01	-0.01	0.03	0.02	0.01	0.01							
IHT(HH Wealth)	-1.43	-1.58	-4.35	5.24	-5.42	-1.89	-3.86	-4.60	1.75	0	0.45	0.17	897.60						
IHT(Earned Income)	0.22	0.19	-0.28	2.83	-1.08	0.01	-0.15	-0.90	0	1.91	0.32	0.12	47.78	64.60					
Log(DC Wealth)	0.01	0.00	-0.01	0.05	-0.01	-0.02	-0.01	-0.02	0	0	0.01	0.00	1.87	0.79	0.05				
Any DC Plan	0.00	0.09	0.04	0.36	-0.06	-0.02	-0.03	-0.07	0	0.15	0.04	0.02	2.33	3.35	0.00	1			
Any DB Plan	0.05	0.02	-0.03	0.42	-0.05	0.00	-0.03	0.01	0	0.09	0.01	0.00	1.15	2.59	0.04	0.17	1		
Early Retirement Age	0.07	-0.01	0.04	-0.12	0.10	0.01	0.01	0.12	0	0	0.01	0.01	-2.28	-1.86	-0.03	-0.03	0.00	1.00	
Normal Retirement	-0.03	0.05	0.08	-0.13	0.08	0.00	0.03	0.04	0	-0.04	0.01	0.01	-3.44	-1.45	-0.01	-0.01	0.00	0.32	1

Table 19

	MCBS total medical costs	MEPS Total medical costs	MCBS total Medicaid costs	MEPS total Medicaid costs	MCBS out of pocket costs	MEPS out of pocket costs
Age 65 to 69	16.97		-482.5		1068.0	
	(0.34)		(1.80)		(1.19)	
Age 70 to 74	1086.5		4075.4		283.7	
	(0.99)		(2.22)		(1.05)	
Age 75 to 79	3571.1		4260.9		912.6	
	(3.19)		(2.36)		(3.08)	
Age 80 to 84	3441.2		3874.1		820.5	
	(3.09)		(2.16)		(2.84)	
Age > 84	4178.1		5408.6		1349.1	
	(3.68)		(2.90)		(4.43)	
Age 50 to 54		-376.5		0		-184.6
		(-1.42)		(.)		(-5.24)
Age 55 to 59		134.9		-553.2		-131.9
		(0.47)		(-0.81)		(-3.65)
Age 60 to 64		0		1334.8		0
		(.)		(1.19)		(.)
Male	-565.5	-448.2	-1247.9	-2874.7	-368.9	-242.2
	(-2.08)	(-1.77)	(-2.00)	(-2.49)	(-4.76)	(-7.26)
Male and Black	2042.1	500.0	23.70	867.5	261.1	101.5
	(1.80)	(0.87)	(0.03)	(0.57)	(1.47)	(1.99)
Male and Hispanic	568.7	-70.78	-455.6	2278.4	20.56	85.66
	(0.59)	(-0.14)	(-0.67)	(0.96)	(0.07)	(1.48)
Male and Less than high school	126.7	708.4	-245.1	1757.7	513.0	58.65
	(0.21)	(1.15)	(-0.37)	(1.19)	(3.26)	(1.06)
Black	565.6	-670.7	1011.1	618.4	-679.1	-418.8
	(0.84)	(-1.99)	(2.21)	(0.56)	(-5.56)	(-10.32)
Hispanic	-37.49	-1090.6	376.2	-212.4	-326.2	-288.1
	(-0.06)	(-3.07)	(0.85)	(-0.18)	(-1.94)	(-6.78)
Less than high school	-806.4	-1009.2	-248.7	-1632.5	-781.5	-192.2
	(-2.06)	(-1.96)	(-0.55)	(-1.55)	(-7.14)	(-4.42)
Some college and above	328.1	510.5	1230.9	-95.96	516.8	202.6
	(1.26)	(2.09)	(2.37)	(-0.10)	(5.74)	(7.38)
Widowed	-149.1	-128.7	-1977.8	-2759.2	-109.1	-32.13
	(-0.49)	(-0.28)	(-4.69)	(-2.01)	(-1.05)	(-0.60)
Single	-272.3	50.10	-1424.5	-932.0	-262.0	-39.76
	(-0.83)	(0.22)	(-3.70)	(-1.02)	(-2.64)	(-1.28)
**Incidence of disease:**						
Cancer	16011.2		3469.7		1734.3	
	(11.47)		(2.90)		(5.08)	
Diabetes	3843.6		4846.1		547.0	
	(3.51)		(1.97)		(1.95)	
Hypertension	4703.4		3467.9		1302.3	
	(5.98)		(3.37)		(5.00)	
Heart disease	8606.6		1918.4		1348.8	
	(8.53)		(1.76)		(4.63)	
Lung disease	5327.0		2055.3		435.1	
	(5.84)		(1.99)		(1.52)	
Stroke	9659.3		4458.2		1447.9	
	(5.02)		(1.76)		(2.29)	
**Maintenance phase of disease:**						
Cancer	2257.9		-344.6		155.1	
	(6.72)		(-1.44)		(1.37)	

Table 19

Diabetes	2501.4		1652.1		166.9	
	(3.88)		(1.16)		(0.90)	
Hypertension	3228.0		12.69		323.2	
	(6.21)		(0.02)		(2.69)	
Heart disease	1042.6		-809.4		252.4	
	(4.01)		(-1.83)		(2.42)	
Lung disease	2839.7		39.61		187.4	
	(8.24)		(0.15)		(1.87)	
Stroke	1163.9		1049.5		-71.48	
	(1.29)		(0.71)		(-0.23)	
**Disease (no information about phase)**						
Cancer		8150.1		3625.1		379.9
		(8.49)		(1.85)		(6.12)
Diabetes		3090.7		929.0		335.8
		(8.47)		(1.05)		(8.07)
Hypertension		1091.4		-992.5		179.9
		(4.59)		(-1.08)		(6.94)
Heart disease		3723.2		3809.6		213.8
		(9.17)		(3.50)		(4.89)
Lung disease		2664.2		2269.6		241.6
		(4.39)		(1.69)		(3.82)
Stroke		5567.7		4142.1		352.0
		(5.42)		(2.69)		(2.27)
Nursing Home Living	43753.8		30210.2		15736.3	
	(43.12)		(29.61)		(21.85)	
ADL 3+-Not in nursing home	7938.1		449.8		1014.5	
	(12.20)		(0.99)		(5.51)	
Eligable for Medicare due to disablity	3288.8		3331.3		559.1	
	(2.87)		(1.89)		(2.03)	
Died	-8633.3		3914.7		584.1	
	(-1.02)		(0.86)		(0.39)	
**Interactions:**						
Diabetes and Heart Disease	104.8		-859.5		-53.41	
	(0.16)		(-1.41)		(-0.30)	
Diabetes and Hypertension	1227.2		-1245.1		163.9	
	(1.71)		(-0.89)		(0.70)	
Hypertension and Heart Disease	244.2		332.4		-293.1	
	(0.42)		(0.40)		(-1.80)	
Hypertension and Stroke	1459.9		-1074.9		651.5	
	(1.39)		(-0.70)		(1.77)	
diclaim_died	29739.2		-2377.2		624.4	
	(2.81)		(-0.52)		(0.39)	
diclaim_nhmliv	-9640.6		-6218.3		-7685.9	
	(-2.66)		(-2.21)		(-4.21)	
**Terminal phase status: (Died and…)**						
Nursing Home Living	-26571.6		-18506.3		-7507.2	
	(-9.26)		(-11.72)		(-6.52)	
Cancer	4015.7		-786.8		270.2	
	(1.57)		(-0.76)		(0.70)	
Diabetes	5207.8		2600.0		203.9	
	(1.89)		(1.50)		(0.29)	
Hypertension	1980.9		20.59		-411.3	
	(0.87)		(0.02)		(-0.61)	
Heart disease	3173.3		427.1		-153.2	

Table 19

	(1.41)		(0.33)		(-0.26)	
Lung disease	-2660.3		590.2		387.9	
	(-1.08)		(0.49)		(0.51)	
Stroke	-473.4		987.0		-576.9	
	(-0.18)		(0.60)		(-0.86)	
Age 65 to 69	32560.5		242.7		540.1	
	(3.12)		(0.06)		(0.38)	
Age 70 to 74	34959.8		-3558.7		765.0	
	(3.78)		(-0.83)		(0.48)	
Age 75 to 79	22848.3		-2540.5		128.3	
	(2.67)		(-0.60)		(0.09)	
Age 80 to 84	24369.6		-1242.6		1192.5	
	(2.95)		(-0.28)		(0.75)	
Age > 84	16821.6		-2969.5		58.71	
	(2.13)		(-0.65)		(0.04)	
Log of Earnings		-18865.1		-12048.5		-904.9
		(-6.98)		(-1.39)		(-2.57)
Constant	4076.0	4444.3	-913.2	5905.5	704.8	882.5
	(3.81)	(8.95)	(-0.50)	(4.59)	(2.62)	(18.29)

t statistics in parentheses

Table 20

	Total Medicare Costs	Medicare Part A Costs	Medicare Part B Costs
Age 65 to 69	-198.2	-1153.1	612.4
	(-0.07)	(0.65)	(-0.51)
Age 70 to 74	556.7	619.0	-199.8
	(0.74)	(1.06)	(-0.44)
Age 75 to 79	2091.6	1594.2	520.6
	(2.75)	(2.67)	(1.14)
Age 80 to 84	2030.7	1768.2	93.45
	(2.66)	(2.92)	(0.21)
Age > 84	2262.5	2072.4	6.276
	(2.91)	(3.35)	(0.01)
Male	-232.4	17.11	-130.9
	(-1.20)	(0.10)	(-1.27)
Male and Black	1418.1	1169.7	585.6
	(1.47)	(1.12)	(1.30)
Male and Hispanic	178.0	-1374.9	405.0
	(0.29)	(-2.07)	(0.89)
Male and Less than high school	394.0	545.8	241.6
	(0.84)	(1.18)	(1.12)
Black	886.4	667.7	350.9
	(1.59)	(1.15)	(1.44)
Hispanic	180.7	648.5	183.7
	(0.44)	(1.29)	(0.65)
Less than high school	-125.8	-478.3	-370.9
	(-0.42)	(-1.63)	(-2.76)
Some college and above	-104.0	-102.2	145.5
	(-0.57)	(-0.60)	(1.52)
Widowed	566.0	269.1	51.48
	(2.62)	(1.30)	(0.48)
Single	682.2	145.8	20.33
	(2.77)	(0.65)	(0.15)
**Incidence of disease:**			
Cancer	10119.4	6942.7	6362.0
	(9.26)	(6.18)	(10.05)

Table 20

Diabetes	2033.9	1668.1	658.7
	(2.58)	(2.04)	(1.92)
Hypertension	1566.0	1245.7	595.3
	(2.89)	(2.22)	(2.65)
Heart disease	5476.9	4290.9	2490.2
	(6.93)	(5.42)	(6.65)
Lung disease	3697.9	2812.8	1094.1
	(5.35)	(3.96)	(3.53)
Stroke	5805.3	6370.7	508.7
	(4.29)	(4.52)	(1.26)
**Maintenance phase of disease:**			
Cancer	1580.2	430.6	1443.2
	(6.38)	(2.06)	(9.26)
Diabetes	1336.1	-239.4	775.4
	(2.90)	(-0.53)	(3.37)
Hypertension	2552.9	1043.1	1431.0
	(6.07)	(2.95)	(6.52)
Heart disease	403.0	-18.23	407.0
	(2.34)	(-0.12)	(4.19)
Lung disease	1856.6	405.6	766.8
	(7.39)	(1.91)	(5.53)
Stroke	625.2	-203.2	396.5
	(1.00)	(-0.34)	(1.46)
Nursing Home Living	4943.3	4241.5	1869.8
	(7.95)	(7.08)	(8.74)
ADL 3+-Not in nursing home	5599.8	3837.3	2433.8
	(11.02)	(7.71)	(10.29)
Eligable for Medicare due to disablity	1627.0	791.9	229.1
	(2.06)	(1.25)	(0.49)
Died	-9359.3	-9778.8	-2326.7
	(-1.26)	(-1.26)	(-1.62)
**Interactions:**			
Diabetes and Heart Disease	88.57	356.9	-106.7
	(0.19)	(0.80)	(-0.43)
Diabetes and Hypertension	1425.4	1124.8	438.2
	(2.81)	(2.27)	(1.72)

Table 20

Hypertension and Heart Disease	36.30	266.2	-221.1
	(0.08)	(0.66)	(-0.96)
Hypertension and Stroke	1152.3	1010.3	338.1
	(1.57)	(1.42)	(1.07)
diclaim_died	28486.3	29226.5	10846.6
	(3.12)	(3.18)	(2.69)
diclaim_nhmliv	-2294.9	-1269.0	-904.1
	(-1.50)	(-0.95)	(-1.45)
**Terminal phase status: (Died and…)**			
Nursing Home Living	-4312.8	-5090.6	-2498.7
	(-1.94)	(-2.19)	(-4.36)
Cancer	3322.9	-84.56	2454.5
	(1.52)	(-0.04)	(2.65)
Diabetes	2930.3	1589.5	2280.4
	(1.29)	(0.68)	(2.66)
Hypertension	1830.2	1595.9	1082.0
	(0.97)	(0.82)	(1.82)
Heart disease	3407.5	2788.7	485.8
	(1.78)	(1.44)	(0.72)
Lung disease	-3527.0	-4020.8	-1916.3
	(-1.71)	(-1.86)	(-2.62)
Stroke	-741.9	-1121.4	-353.2
	(-0.33)	(-0.50)	(-0.43)
Age 65 to 69	28195.7	26800.2	9393.9
	(2.90)	(2.72)	(4.47)
Age 70 to 74	31579.7	34850.7	7536.7
	(3.86)	(4.07)	(4.48)
Age 75 to 79	21966.9	26067.6	5492.8
	(2.85)	(3.05)	(3.52)
Age 80 to 84	20795.5	23722.7	4675.8
	(2.91)	(3.20)	(3.22)
Age > 84	16400.8	19887.1	3109.5
	(2.38)	(2.75)	(2.33)
Constant	1993.9	-58.14	1393.9
	(2.75)	(-0.10)	(3.21)

Table 20

t statistics in parentheses

Table 21

	New Medicare Enrollees	Existing Medicare Enrollees not in Part B	All Medicare Eligible
Male	-0.0930	-0.0775	-0.217
	(-0.91)	(-0.73)	(-6.52)
Black	-0.0388	-0.104	-0.132
	(-0.22)	(-0.71)	(-2.44)
Hispanic	0.155	-0.264	-0.252
	(0.82)	(-1.69)	(-5.11)
Less than high school	-0.217	0.103	-0.0167
	(-1.38)	(0.72)	(-0.38)
Some college and above	-0.0696	-0.0316	-0.0528
	(-0.61)	(-0.28)	(-1.41)
widowed	0.279	-0.134	0.0883
	(1.57)	(-0.85)	(1.96)
iearnx	-0.00495	-0.00111	-0.00334
	(-3.43)	(-0.85)	(-7.27)
work	-0.858	-0.592	-0.705
	(-8.21)	(-5.54)	(-19.97)
Cancer	0.160	0.0141	-0.0284
	(1.19)	(0.11)	(-0.70)
Hypertension	0.0458	0.201	0.0897
	(0.36)	(1.97)	(2.53)
Heart disease	-0.0440	0.0688	0.0402
	(-0.32)	(0.37)	(0.72)
Stroke	0.267	0.200	-0.0142
	(1.27)	(0.69)	(-0.13)
ADL 2-Not in nursing home	0.0675		
	(0.37)		
adl2	0.0813		
	(0.28)		
ADL 3+-Not in nursing home	-0.228	-0.0621	-0.0983
	(-0.67)	(-0.30)	(-1.42)
Obese(bmi>=30)	-0.127	0.122	0.0580
	(-0.69)	(1.17)	(1.57)

Table 21

Ever smoked	-0.0272	-0.0567	-0.0530
	(-0.27)	(-0.48)	(-1.32)
Eligable for Medicare due to disablity	0.132	-1.445	0.169
	(0.64)	(-5.27)	(2.02)
Diabetes & Hypertension	-0.156		
	(-0.88)		
Diabetes & Heart Disease	-0.127	0.116	
	(-0.53)	(0.48)	
Hypertension & Obesity	0.153		
	(0.66)		
DI Claim and 3+ ADLs	0.239		
	(0.45)		
Max(age, 75)		-0.0806	
		(-4.60)	
Min(0, age - 75)		-0.0558	-0.00114
		(-2.47)	(-0.22)
Diabetes		-0.000541	-0.0251
		(-0.00)	(-0.65)
Lung disease		0.0750	
		(0.56)	
hibpe_stroke		-0.299	0.171
		(-0.82)	(1.34)
hearte_smokev		-0.145	0.120
		(-0.64)	(1.75)
Age Spline Knot at 65			0.00101
			(0.10)
Age Spline Knot at 75			0.0991
			(16.82)
Constant	1.353	4.999	1.448
	(8.90)	(4.15)	(2.20)

t statistics in parentheses

Table 22

	Age 55-64			Age 65 and over		
	NHEA 2004 ($) (1)	FEM 2004, unadjusted ($) (2)	Adjustment factor (1)/(2)	NHEA 2004 ($) (3)	FEM 2004, unadjusted ($) (4)	Adjustment factor (3)/(4)
Payment sources						
Total	7,787	7,412	1.05	18,424	17,086	1.08
Medicare	706	675	1.05	10,016	9,264	1.08
Medicaid	1,026	638	1.61	2,047	1,367	1.50

Table 23

	Medicare Part D Enrollment
Max(age, 75)	0.0325
	(12.15)
Min(0, age - 75)	-0.0216
	(-12.66)
Male	-0.0411
	(-2.48)
Black	0.129
	(4.75)
Hispanic	0.281
	(9.55)
Less than high school	0.110
	(5.67)
Some college and above	-0.0660
	(-3.72)
married	0.0167
	(0.99)
Earned Income ($1000s)	-0.00420
	(-13.81)
work	-0.0996
	(-4.14)
Cancer	-0.0104
	(-0.57)
Diabetes	0.0232
	(1.31)
Hypertension	0.0467
	(2.77)
Stroke	0.0657
	(2.85)
Heart disease	0.0390
	(2.51)
ADL 3+-Not in nursing home	-0.00813
	(-0.30)

Table 23

Ever smoked	-0.00268
	(-0.17)
Eligable for Medicare due to disablity	0.536
	(11.69)
Constant	-2.163
	(-11.26)

t statistics in parentheses

Table 24

	Medicare Part D Expenditures
Max(age, 75)	-22.39
	(-3.41)
Min(0, age - 75)	-11.37
	(-5.00)
Male	-221.7
	(-11.81)
Black	483.4
	(14.39)
Hispanic	157.5
	(3.49)
Less than high school	494.5
	(20.75)
Some college and above	-87.30
	(-4.54)
widowed	164.0
	(8.06)
Diabetes	205.2
	(9.49)
Hypertension	162.7
	(7.96)
Stroke	114.3
	(4.02)
Heart disease	159.6
	(8.21)
Lung disease	217.6
	(9.00)
Current smoking	143.2
	(4.72)
Eligable for Medicare due to disablity	630.8
	(10.97)
IADL 1-Not in nursing home	157.8
	(6.37)

Table 24

IADL 2+-Not in nursing home	291.4
	(9.01)
ADL 2-Not in nursing home	65.36
	(2.44)
2 ADLs	92.94
	(2.37)
ADL 3+-Not in nursing home	228.4
	(4.42)
Constant	1560.5
	(6.58)

t statistics in parentheses

Table 25

	Baseline Estimates		
	Year		
	2010	2030	2050
Population size (Million)	98.19	126.97	146.30
Population 65+ (Million)	43.69	72.00	80.61
Prevalence of selected conditions			
obesity (BMI >=30) (%)	35%	49%	55%
over weight (25<=BMI<30) (%)	35%	31%	28%
Ever-smoked	56%	44%	32%
Smoking now	15%	9%	6%
Diabetes	20%	33%	39%
Heart disease	23%	30%	32%
Hypertension	55%	67%	69%
Labor participation			
Working (%)	46%	41%	41%
Average earnings if working ($2010)	$ 47,285.48	$ 55,200.23	$ 69,913.09
Government revenues from aged 51+ (Billion $2010)			
Federal personal income taxes	$ 376.62	$ 564.27	$ 929.23
Social security payroll taxes	$ 118.41	$ 178.34	$ 271.47
Medicare payroll taxes	$ 31.29	$ 42.63	$ 63.68
Total Revenue	$ 526.31	$ 785.24	$ 1,264.38
Government expenditures from aged 51+ (Billion $2010)			
Old Age and Survivors Insurance benefits (OASI)	$ 663.81	$ 1,232.69	$ 1,641.88
Disability Insurance benefits (DI)	$ 30.51	$ 34.52	$ 53.27
Supplementary Security Income (SSI)	$ 19.61	$ 26.36	$ 36.87
Medicare costs	$ 549.50	$ 1,316.60	$ 2,541.89
Medicaid costs	$ 163.74	$ 330.50	$ 827.51
Medicare + Medicaid	$ 713.24	$ 1,647.10	$ 3,369.41
Total medical costs for aged 51+ (Billion $2010)	$ 1,400.55	$ 2,994.40	$ 5,912.06

Table 26

	Quality Adjusted Life Year
Has exactly 1 IADL	-0.0325<br>(-37.74)
Has 2 or more IADLs	-0.0480<br>(-39.78)
Has exactly 1 ADL	-0.0683<br>(-98.28)
Has exactly 2 ADLs	-0.115<br>(-110.89)
Has 3 or more ADLs	-0.158<br>(-151.53)
Cancer	-0.0153<br>(-27.59)
Diabetes	-0.0352<br>(-67.24)
Heart disease	-0.0431<br>(-94.30)
Hypertension	-0.0308<br>(-80.96)
Lung disease	-0.0436<br>(-65.67)
Stroke	-0.0432<br>(-58.61)
Current smoking	-0.0357<br>(-69.83)
obese	-0.0362<br>(-84.70)
Single	-0.0186<br>(-36.12)
Widowed	-0.0120<br>(-25.14)
Constant	0.893<br>(2806.86)

Table 26

t statistics in parentheses

Table 27

Variable	Wave 4 FEM mean	Wave 4 HRS mean	Wave 4 p-value	Wave 7 FEM mean	Wave 7 HRS mean	Wave 7 p-value	Wave 9 FEM mean	Wave 9 HRS mean	Wave 9 p-value
adl1	0.07	0.07	0.51	0.08	0.08	0.69	0.09	0.09	0.68
adl2	0.04	0.03	0.39	0.03	0.04	0.14	0.04	0.03	0.09
adl3p	0.04	0.04	0.18	0.05	0.04	0.00	0.05	0.05	0.04
age	65.91	65.87	0.61	69.73	69.76	0.72	72.28	72.31	0.72
anyhi	0.94	0.94	0.60	0.97	0.96	0.00	0.98	0.97	0.01
black	0.09	0.09	0.29	0.09	0.09	0.91	0.08	0.08	0.83
bmi	27.66	26.85	0.00	28.16	27.26	0.00	28.53	27.80	0.00
cancre	0.10	0.10	0.52	0.15	0.15	0.22	0.19	0.18	0.14
dbclaim	0.00	0.04	0.00	0.06	0.08	0.00	0.09	0.00	0.00
diabe	0.12	0.12	0.62	0.19	0.18	0.00	0.23	0.22	0.48
diclaim	0.04	0.04	0.58	0.03	0.03	0.02	0.02	0.02	0.00
died	0.00	0.00		0.00	0.00		0.00	0.00	
hatotax	306.08	274.20	0.00	390.53	375.76	0.00	390.30	439.42	0.00
hearte	0.20	0.20	0.65	0.27	0.25	0.00	0.31	0.30	0.00
hibpe	0.42	0.42	0.42	0.55	0.54	0.21	0.62	0.63	0.10
hicap	15173.27	14742.81	0.54	16779.25	13420.51	0.00	15490.40	16080.52	0.40
hicap_nonzero	0.66	0.74	0.00	0.72	0.71	0.01	0.70	0.69	0.00
hispan	0.06	0.06	0.63	0.07	0.06	0.00	0.07	0.06	0.03
iadl1	0.05	0.05	0.63	0.05	0.05	0.24	0.06	0.06	0.60
iadl2p	0.03	0.03	0.18	0.04	0.03	0.00	0.04	0.04	0.04
iearnx	12.38	13.94	0.00	11.30	12.45	0.00	7.65	10.72	0.00
lunge	0.07	0.07	0.56	0.11	0.10	0.01	0.12	0.12	0.59
male	0.45	0.45	0.90	0.44	0.45	0.29	0.44	0.44	0.37
nhmliv	0.00	0.00		0.00	0.00		0.00	0.00	
smoken	0.18	0.17	0.00	0.14	0.14	0.59	0.11	0.12	0.00
smokev	0.60	0.60	0.99	0.59	0.59	0.36	0.57	0.58	0.11
ssiclaim	0.04	0.04	0.71	0.02	0.02	0.38	0.02	0.02	0.08
stroke	0.06	0.06	0.06	0.08	0.08	0.91	0.10	0.10	0.51
wlth_nonzero	0.97	0.97	0.53	0.97	0.98	0.00	0.97	0.98	0.00
work	0.43	0.43	0.28	0.32	0.34	0.00	0.24	0.28	0.00
