# Supplementary material for: The Long-Term Benefits of Increased Aspirin Use by At-Risk Americans Aged 50 and Older
Source: PLoS One. 2016 Nov 30;11(11):e0166103. doi: 10.1371/journal.pone.0166103 (PMC5130201; doi:10.1371/journal.pone.0166103)
Supplement: S2 File — Table A. Health Impact of Aspirin Reported by Large Meta-Analyses and Table B. Potential for Aspirin to Reduce Cancer Incidence in the Population Aged over 50 and Table C. Health Impact of Guideline Adherence for High-Risk Population and Table D. Costs and Benefits of Guideline Adherence for High-Risk Population, $2015 Thousands and Table E. Sensitivity Analysis: Health Impact of Increased Aspirin Use under Pessimistic Assumptions (PDF) [file pone.0166103.s002.pdf]

APPENDIX TABLES

Table A Health Impact of Aspirin Reported by Large Meta-Analyses

Meta-Analysis	Population (Primary or Secondary CVD Prevention)	Effect of Aspirin Therapy Compared to Placebo
Seshasai et al., 2012 [1]	Primary	**Decreased nonfatal MI (RR 0.80 95% CI 0.67-0.96)**<br>Decreased total CVD events (RR 0.90 95% CI 0.85-0.96)<br>Increased total bleeds (RR 1.7 95% CI 1.17-2.46)<br>Increased nontrivial bleeds (RR 1.31 95% CI 1.14-1.5)<br>**Decreased all-cause mortality (RR 0.94 95% CI 0.88-1.00)**
Berger et al., 2011 [2]	Primary	Decreased major CVD events (RR 0.9 95% CI 0.85-0.96)<br>Increased hemorrhagic stroke (RR 1.35 95% CI 1.01-1.81)<br>Increased major bleeds (RR 1.62 95% CI 1.31-2)
Raju et al., 2011 [3]	Primary	Decreased major CVD events (RR 0.88 95% CI 0.83-0.94)<br>Decreased ischemic stroke (RR 0.86 95% CI 0.75-0.98)<br>Increased hemorrhagic stroke (RR 1.35 95% CI 1.01-0.94)<br>Increased GI bleeds (RR 1.37 95% CI 1.15-1.62)<br>Increased major bleeds (RR 1.66 95% CI 1.41-1.95)
Rothwell et al., 2011 [4]	Primary or Secondary	Decreased colorectal cancer mortality (RR 0.6 95% CI 0.43-0.81)<br>Decreased oesophageal cancer mortality (RR 0.42 95% CI 0.25-0.71)<br>Decreased lung cancer mortality (RR 0.71 95% CI 0.58-0.89)<br>Decreased all solid cancer mortality (RR 0.75 95% CI 0.67-0.84)<br>Decreased all cancer mortality (RR 0.78 95% CI 0.7-0.87)
Rothwell et al., 2010 [5]	Primary or Secondary	Decreased colorectal cancer incidence after ≥ 2.5 years of aspirin treatment (RR 0.69 95% CI 0.51-0.93), ≥ 5 years of aspirin treatment (RR 0.62 95% CI 0.43-0.94), or an unspecified treatment duration (RR 0.75 95% CI 0.56-0.97)
Baigent et al., 2009 [6]	Primary	Decreased major coronary events in men (RR 0.77 95% CI 0.67-0.89)<br>Decreased ischemic stroke in women (RR 0.77 95% CI 0.59-0.99)
	Secondary	Decreased major coronary events in men (RR 0.81 95% CI 0.72-0.92)
Berger et al., 2008 [7]	Secondary	**Decreased total CVD events (OR 0.794 95% CI 0.715-0.882)**<br>Decreased total MI (OR 0.738 95% CI 0.598-0.91)<br>**Decreased total stroke (OR 0.754 95% CI 0.654-0.869)**<br>**Decreased all-cause mortality (OR 0.872 95% CI 0.764-0.995)**<br>Increased major bleeding (OR 2.332 95% CI 1.599-3.399)
Berger et al., 2006 [8]	Primary	Decreased total CVD events in men (OR 0.86 95% CI 0.78-0.94) and women (OR 0.88 95% CI 0.79-0.99)<br>Decreased total MI in men (OR 0.68 95% CI 0.54-0.86)<br>**Decreased total stroke in women (OR 0.83 95% CI 0.7-0.97)**<br>Decreased ischemic stroke in women (OR 0.76 95% CI 0.63-0.93)<br>**Increased total stroke in men (OR 1.13 95% CI 0.96-1.33)**<br>Increased hemorrhagic stroke in men (OR 1.69 95% CI 1.04-2.73)<br>Increased major bleeding in men (OR 1.72 95% CI 1.35-2.2) and women (OR 1.69 95% CI 01.13-2.52)

Table A Health Impact of Aspirin (cont.)

McQuaid and Laine, 2006 [9]	Primary or Secondary	**Increased any major bleeding (RR 1.71 95% CI 1.41-2.08)**<br>Increase major GI bleeding (RR 2.07 95% CI 1.61-2.66)<br>Increased intracranial bleeding (RR 1.65 95% CI 1.12-2.44)<br>Increased non-GI, non-intracranial bleeding (RR 1.72 95% CI 1.39-2.13)<br>Increased fatal intracranial bleeding (RR 2.52 95% CI 1.06-5.99)

Only statistically significant findings are shown, with the exception of non-statistically significant findings that were included in the simulations. Findings used in the simulations are shown in bold font. RR - risk ratio, OR - odds ratio, CVD - cardiovascular disease, MI - myocardial infarction, GI – gastrointestinal.

Table B Potential for Aspirin to Reduce Cancer Incidence in the Population Aged over 50

Incident Cancer Site	Relative Incidence*	Risk Ratio ** Conservative Estimate	Best Estimate
Prostate	24%	0.95	0.9
Breast	16%	0.95	0.9
Rectum bowel colon	13%	0.7	0.65
Bronchia bronchus lung chest-NFS	8%	1	0.95
Stomach	1%	0.75	0.7
Other	37%	1	1
**Total**	**100%**	**0.94**	**0.91**

*: Corresponds to the share of new incident cancers observed in Health and Retirement Study at ages over 50 (years 1995-2008). “Incident” means that the respondent reported no cancer in the prior wave, but reported cancer in reference wave; **: Site-specific risk ratio estimates of Cuzick et al., 2014 [10].

Table C Health Impact of Guideline Adherence for High-Risk Population

	High-Risk Women*				High-Risk Men*			
	Status Quo	Guideline Adherence**			Status Quo	Guideline Adherence**		
	Mean	Mean	Difference with Status Quo [95% CI]		Mean	Mean	Difference with Status Quo [95% CI]	
Panel A. Cumulative disease incidence at age 79 (per-thousand)***								
Cardiovascular disease	441	422	-18.6	[-26.5 to -11.8]	527	510	-17.6	[-29.1 to -5.9]
Stroke	216	194	-21.3	[-29.2 to -14.5]	232	239	6.7	[-5.3 to 19.2]
Cancer	236	231	-5.2	[-9.4 to -1]	328	322	-6.3	[-9.6 to -2.9]
Gastrointestinal bleeding	54	80	26.4	[19 to 35.6]	81	107	26.2	[9.3 to 44.1]
Panel B. Expected outcomes at age 51†								
Life expectancy (years)	27.9	28.3	0.40	[0.31 to 0.51]	28.8	29.1	0.30	[0.11 to 0.47]
Disability-free life-years	19.8	20.0	0.20	[0.13 to 0.26]	22.8	22.9	0.13	[0.05 to 0.23]
Quality-adjusted life-years	22.6	22.9	0.30	[0.23 to 0.38]	24.1	24.3	0.21	[0.08 to 0.34]

*: Simulations results from age 55 until death among the subset of women with a predicted stroke risk higher than or equal to 3% at age 55; Simulation results from age 51 until death among the subset of men with a predicted coronary heart disease risk higher than or equal to 4% at age 51; **: Individuals follow 2009 USPSTF guidelines for primary prevention of heart disease and stroke until age 79 and use aspirin for secondary prevention at all ages; ***: Total incident cases by age 79 for a thousand individuals without the disease at age 51; †: Lifetime outcomes are calculated from age 55 until death for high-risk women and from age 51 until death for high-risk men. Disability-free life expectancy refers to reporting no instrumental activity of daily living or activity of daily living limitations and not living in a nursing home. Quality-adjusted life-years adjust length of life for quality based on a person's chronic conditions and functional status. 95% confidence intervals with regard to the uncertainty of the effectiveness of aspirin are presented in brackets.

Table D Costs and Benefits of Guideline Adherence for High-Risk Population, $2015 Thousands

	Guideline Adherence*: Difference with Status Quo			
	Women Eligible for Aspirin Therapy**		Men Eligible for Aspirin Therapy**	
	Mean	95% CI	Mean	95% CI
Value of expected quality-adjusted life-years gained	22.5	[16.71 to 28.5]	15.3	[5.56 to 24.01]
Expected health-care and medication costs				
Health care excluding gastrointestinal bleeds	6.7	[3.37 to 10.25]	5.1	[0.1 to 9.74]
Gastrointestinal bleeds	0.15	[0.12 to 0.19]	0.13	[0.05 to 0.21]
Aspirin medication	0.08	[0.08 to 0.08]	0.08	[0.08 to 0.08]
Total	6.9	[3.6 to 10.5]	5.3	[0.33 to 9.93]
Net value per capita	15.6	[10.78 to 20.1]	10.0	[2.88 to 16.79]
Incremental cost-effectiveness ratio	50.8	[29.21 to 71.14]	48.1	[1.84 to 87.55]

*: Individuals follow 2009 USPSTF guidelines for primary prevention of heart diseases and stroke until age 79 and use aspirin for secondary prevention at all ages; **: The subset of women with a predicted stroke risk higher than or equal to 3% at age 55; ***: The subset of men with a predicted coronary heart disease risk higher than or equal to 4% at age 51. All amounts are in present value at age 51, computed with a 3% discount rate. Quality-adjusted life-years adjust length of life for quality based on a person's chronic conditions and functional status. 95% confidence intervals with regard to the uncertainty of the effectiveness of aspirin are presented in brackets.

Table E Sensitivity Analysis: Health Impact of Increased Aspirin Use under Pessimistic Assumptions

	Status Quo	Guideline Adherence*			Universal Eligibility***		
	Mean	Mean	Difference with Status Quo [95% CI]		Mean	Difference with Status Quo [95% CI]	
Panel A. Cumulative disease incidence at age 79 (per thousand)***							
Cardiovascular disease	487	474	-12.7	[-23.7 to -4.1]	467	-20.0	[-35.3 to -7.6]
Stroke	235	232	-3.0	[-12.5 to 6]	229	-6.2	[-16.4 to 4.9]
Cancer	293	294	1.0	[-0.6 to 3.7]	294	1.0	[-0.6 to 3.9]
Gastrointestinal bleeding	67	97	30.7	[1.6 to 70.8]	112	45.6	[1.6 to 109.6]
Panel B. Expected outcomes at age 51							
Life expectancy (years)	30.2	30.4	0.19	[0.01 to 0.39]	30.4	0.22	[0.04 to 0.44]
Disability-free life-years	22.8	22.9	0.08	[0.01 to 0.17]	22.9	0.11	[0.03 to 0.22]
Quality-adjusted life-years	24.8	24.9	0.13	[0 to 0.27]	24.9	0.16	[0.04 to 0.31]

*: Individuals follow 2009 USPSTF guidelines for primary prevention of heart diseases and stroke until age 79 and use aspirin for secondary prevention at all ages; **: All individuals over age 50 are assigned to use aspirin daily; ***: Total incident cases by age 79 for a thousand individuals without the disease at age 51; † Calculated from age 51 until death. Disability-free life expectancy refers to reporting no instrumental activity of daily living or activity of daily living limitations and not living in a nursing home. Quality-adjusted life-years adjust length of life for quality based on a person's chronic conditions and functional status. 95% confidence intervals with regard to the uncertainty of the effectiveness of aspirin are presented in brackets.

Table F Sensitivity Analysis: Costs and Benefits of Increased Aspirin Use under Pessimistic Assumptions, $2015 Thousands

	Difference with Baseline			
	Guideline Adherence*		Universal Eligibility**	
	Mean	95% CI	Mean	95% CI
Value of expected quality-adjusted life-years gained	9.1	[0.28 to 19.09]	11.2	[2.34 to 21.07]
Expected health-care and medication costs				
Health care excluding gastrointestinal bleeds	4.2	[-0.17 to 10.28]	3.8	[-0.66 to 9.95]
Gastrointestinal bleeds	0.1	[0.02 to 0.33]	0.2	[0.02 to 0.53]
Aspirin medication	0.2	[0.18 to 0.2]	0.3	[0.32 to 0.34]
Total	4.6	[0.15 to 10.58]	4.4	[-0.24 to 10.48]
Net value per capita	4.6	[-0.47 to 10.16]	6.8	[1.57 to 12.73]
Incremental cost-effectiveness ratio	92.9	[7.07 to 180.76]	60.4	[-6.02 to 123.87]

*: Individuals follow 2009 USPSTF guidelines for primary prevention of heart disease and stroke until age 79 and use aspirin for secondary prevention at all ages; **: All individuals over age 50 are assigned to use aspirin daily. All amounts are in present value at age 51, computed with a 3% discount rate. Quality-adjusted life-years adjust length of life for quality based on a person's chronic conditions and functional status. 95% confidence intervals with regard to the uncertainty of the effectiveness of aspirin are presented in brackets.

Table G Sensitivity Analysis: Health Impact of *Guideline Adherence** scenario under Alternative Cancer Parameters

	Status Quo	Pessimistic cancer reduction (RR = 0.96)			Optimistic cancer reduction (RR = 0.91)		
	Mean	Mean	Difference with Status Quo [95% CI]		Mean	Difference with Status Quo [95% CI]	
Panel A. Cumulative disease incidence at age 79 (per thousand)**							
Cardiovascular disease	487	475	-11.5	[-23.2 to -0.7]	475	-11.3	[-23.2 to -0.7]
Stroke	235	233	-2.5	[-12 to 7.4]	233	-2.4	[-12 to 7.4]
Cancer	293	291	-2.0	[-7.3 to 3]	287	-6.6	[-13.7 to -0.1]
Gastrointestinal bleeding	67	83	16.0	[3.6 to 30]	83	16.0	[3.6 to 30]
Panel B. Expected outcomes at age 51***							
Life expectancy (years)	30.2	30.4	0.25	[0.01 to 0.49]	30.4	0.26	[0.02 to 0.52]
Disability-free life-years	22.8	22.9	0.11	[0 to 0.22]	22.9	0.12	[0 to 0.23]
Quality-adjusted life-years	24.8	25.0	0.18	[0.02 to 0.34]	25.0	0.19	[0.03 to 0.35]

*: Individuals follow 2009 USPSTF guidelines for primary prevention of heart diseases and stroke until age 79 and use aspirin for secondary prevention at all ages; ***: Total incident cases by age 79 for a thousand individuals without the disease at age 51; ***: Calculated from age 51 until death. Disability-free life expectancy refers to reporting no instrumental activity of daily living or activity of daily living limitations and not living in a nursing home. Quality-adjusted life-years adjust length of life for quality based on a person's chronic conditions and functional status. 95% confidence intervals with regard to the uncertainty of the effectiveness of aspirin are presented in brackets.

Table H Sensitivity Analysis: Costs and Benefits of of *Guideline Adherence** scenario under Alternative Cancer Parameters, $2015 Thousands

	Difference with Baseline<br>Pessimistic cancer reduction<br>(RR = 0.96)		Optimistic cancer reduction<br>(RR = 0.91)	
	Mean	95% CI	Mean	95% CI
Value of expected quality-adjusted life-years gained	12.5	[1.39 to 24.26]	13.4	[1.97 to 25.79]
Expected health-care and medication costs				
Health care excluding gastrointestinal bleeds	5.0	[-0.95 to 12.79]	5.0	[-1.32 to 12.66]
Gastrointestinal bleeds	0.1	[0.02 to 0.13]	0.1	[0.02 to 0.14]
Aspirin medication	0.1	[0.06 to 0.06]	0.1	[0.06 to 0.06]
Total	5.1	[-0.83 to 12.92]	5.2	[-1.21 to 12.81]
Net value per capita	7.4	[1.4 to 13.74]	8.2	[1.94 to 15.16]
Incremental cost-effectiveness ratio	62.5	[-19.88 to 114.85]	60.3	[-22.37 to 112.54]

*: Individuals follow 2009 USPSTF guidelines for primary prevention of heart disease and stroke until age 79 and use aspirin for secondary prevention at all ages. All amounts are in present value at age 51, computed with a 3% discount rate. Quality-adjusted life-years adjust length of life for quality based on a person's chronic conditions and functional status. 95% confidence intervals with regard to the uncertainty of the effectiveness of aspirin are presented in brackets.

REFERENCES

1. Seshasai SR, Wijesuriya S, Sivakumaran R, Nethercott S, Erqou S, Sattar N, et al. Effect of aspirin on vascular and nonvascular outcomes: meta-analysis of randomized controlled trials. Arch Intern Med. 2012;172(3):209-16. doi: 10.1001/archinternmed.2011.628. PubMed PMID: 22231610.

2. Berger JS, Lala A, Krantz MJ, Baker GS, Hiatt WR. Aspirin for the prevention of cardiovascular events in patients without clinical cardiovascular disease: A meta-analysis of randomized trials. American Heart Journal. 2011;162(1):115-U59. doi: 10.1016/j.ahj.2011.04.006. PubMed PMID: WOS:000292542400025.

3. Raju N, Sobieraj-Teague M, Hirsh J, O'Donnell M, Eikelboom J. Effect of aspirin on mortality in the primary prevention of cardiovascular disease. Am J Med. 2011;124(7):621-9. doi: 10.1016/j.amjmed.2011.01.018. PubMed PMID: 21592450.

4. Rothwell PM, Fowkes FGR, Belch JF, Ogawa H, Warlow CP, Meade TW. Effect of daily aspirin on long-term risk of death due to cancer: analysis of individual patient data from randomised trials. The Lancet. 2011;377(9759):31-41.

5. Rothwell PM, Wilson M, Elwin CE, Norrving B, Algra A, Warlow CP, et al. Long-term effect of aspirin on colorectal cancer incidence and mortality: 20-year follow-up of five randomised trials. Lancet. 2010;376(9754):1741-50. doi: 10.1016/S0140-6736(10)61543-7. PubMed PMID: 20970847.

6. Baigent C, Blackwell L, Collins R, Emberson J, Godwin J, Peto R, et al. Aspirin in the primary and secondary prevention of vascular disease: collaborative meta-analysis of individual participant data from randomised trials. Lancet. 2009;373(9678):1849-60. doi: 10.1016/S0140-6736(09)60503-1. PubMed PMID: 19482214; PubMed Central PMCID: PMCPMC2715005.

7. Berger JS, Brown DL, Becker RC. Low-dose aspirin in patients with stable cardiovascular disease: a meta-analysis. Am J Med. 2008;121(1):43-9. doi: 10.1016/j.amjmed.2007.10.002. PubMed PMID: 18187072.

8. Berger JS, Roncaglioni MC, Avanzini F, Pangrazzi I, Tognoni G, Brown DL. Aspirin for the primary prevention of cardiovascular events in women and men: a sex-specific meta-analysis of randomized controlled trials. JAMA. 2006;295(3):306-13. doi: 10.1001/jama.295.3.306. PubMed PMID: 16418466.

9. McQuaid KR, Laine L. Systematic review and meta-analysis of adverse events of low-dose aspirin and clopidogrel in randomized controlled trials. Am J Med. 2006;119(8):624-38. doi: 10.1016/j.amjmed.2005.10.039. PubMed PMID: 16887404.

10. Cuzick J, Thorat M, Bosetti C, Brown P, Burn J, Cook N, et al. Estimates of benefits and harms of prophylactic use of aspirin in the general population. Annals of Oncology. 2014:mdu225.
