# Supplementary material for: The Long-Term Benefits of Increased Aspirin Use by At-Risk Americans Aged 50 and Older
Source: PLoS One. 2016 Nov 30;11(11):e0166103. doi: 10.1371/journal.pone.0166103 (PMC5130201; doi:10.1371/journal.pone.0166103)
Supplement: S3 File — A. Sensitivity analysis: health impact and cost-effectiveness of increased aspirin use under pessimistic assumptions and B. Sensitivity analysis: health impact of increased aspirin use under alternative cancer parameters (PDF) [file pone.0166103.s003.pdf]

APPENDIX

A. SENSITIVITY ANALYSIS: HEALTH IMPACT AND COST-EFFECTIVENESS OF INCREASED ASPIRIN USE UNDER PESSIMISTIC ASSUMPTIONS

This article aims to develop and study a realistic scenario in which guidelines for aspirin that were in effect in 2011-2012 were fully followed. However, cancer risk reduction implemented in the baseline simulations has yet to be confirmed by large-scale, randomized clinical trials, and a recent Japanese study found no reduction in all-cause mortality in a primary care setting [1]. Similarly, in a population setting, some patients may need to take a larger dose of aspirin to reach cardiovascular risk reductions of the scale found in randomized clinical trials. Rather than study the implications of each assumption individually, we implemented "worst-case" Guideline Adherence and Universal Eligibility scenarios in which we consider more pessimistic alternative parameters. These scenarios depart from those presented in the article in three ways:

1. Aspirin is assumed to have no impact on cancer incidence.
2. Aspirin is assumed to have no impact on all-cause mortality in a primary prevention setting.
3. Individuals need to take a higher-dose daily tablet of aspirin to obtain its health benefits, and thus face higher gastrointestinal-bleed risk ratios and medication costs. The risk ratios implemented are drawn from a distribution with a point estimate of 2.26 (95% CI 1.24-4.14), which are consistent with medium-dose use of aspirin (162.5-325 mg daily) [2].
4. The direct costs of aspirin are those of brand-name drugs rather than generics. We consider an annual value of $23.71, based on a unit cost of $0.065 per 325 mg tablet. This unit cost corresponds to the 200-count package price for Bayer aspirin on the website drugstore.com (accessed Mar. 14, 2016).

The results from this analysis are presented in tables E and F in S2 File of the Supplemental Information. Compared to the results shown in Table 1, these pessimistic scenarios result in about twice as many gastrointestinal bleeds and lower life-expectancy gains. Despite this, Supporting Table F indicates that cardiovascular disease prevention would remain important enough for the interventions to carry a positive net value. This net value is significant at the 5% level in the Universal Eligibility scenario and at the 10% level in the Guideline Adherence scenario (90% CI 0.32-9.52).

B. SENSITIVITY ANALYSIS: HEALTH IMPACT OF INCREASED ASPIRIN USE UNDER ALTERNATIVE CANCER PARAMETERS

As mentioned in Section 2.2.4, we do not randomly draw estimates for the impact of aspirin on cancer prevention because our literature review did not identify a distribution for this

parameter. For that reason, we implemented in our simulations a parameter consistent with the estimates considered “conservative” by Cuzick *et al.* (2014)[3]. To test for the sensitivity of our results to this parameter, we consider two additional scenarios. First, we note that the clinical trial evidence regarding the preventive effect of aspirin is strongest for colorectal cancer, and the impact of aspirin for other sites is supported by case-control and cohort studies but has not been confirmed by randomized clinical trials[1]. We thus consider a “pessimistic” scenario under which aspirin only prevents gastrointestinal cancer, which represents 13% of all cancer incidence in the population aged over 50 (Table B in S2 File). Cuzick *et al.* estimates that aspirin conservatively reduces incidence of gastrointestinal cancer by 30%. In the “pessimistic” scenario, this means that aspirin would decrease overall cancer incidence by 30% * 13% = 3.9%, corresponding to a risk ratio of 0.96. The “optimistic” scenario which aims to produce an upper bound for the effectiveness of aspirin uses the “best estimates” of Cuzick *et al.* of Supporting Table B, which correspond to a risk ratio of 0.91.

The results of this analysis are presented in tables G and H in S2 F. As expected, these scenarios noticeably impact cancer incidence at age 79. Notably, the optimistic cancer reduction significantly reduces cancer estimates against the status quo. Overall, we find that these alternative parameters have very limited impact on other outcomes, such as life expectancy, expected QALYs, the net value per capita, and incremental cost-effectiveness ratio of increasing aspirin use.

REFERENCES

1. Ikeda Y, Shimada K, Teramoto T, Uchiyama S, Yamazaki T, Oikawa S, et al. Low-dose aspirin for primary prevention of cardiovascular events in Japanese patients 60 years or older with atherosclerotic risk factors: a randomized clinical trial. JAMA. 2014;312(23):2510-20. doi: 10.1001/jama.2014.15690. PubMed PMID: 25401325.

2. McQuaid KR, Laine L. Systematic review and meta-analysis of adverse events of low-dose aspirin and clopidogrel in randomized controlled trials. Am J Med. 2006;119(8):624-38. doi: 10.1016/j.amjmed.2005.10.039. PubMed PMID: 16887404.

3. Cuzick J, Thorat M, Bosetti C, Brown P, Burn J, Cook N, et al. Estimates of benefits and harms of prophylactic use of aspirin in the general population. Annals of Oncology. 2014:mdu225.

[1] In Table 1 of Cuzick et al. (2014), colorectal is the only cancer site for which the incidence impact of aspirin is supported by multiple clinical trials.
